# Metformin Restores Mitochondrial Function and Neurogenesis in *POLG* Patient‐Derived Brain Organoids

**DOI:** 10.1002/advs.202417721

**Published:** 2025-12-08

**Authors:** Zhuoyuan Zhang, Tsering Yangzom, Ning Lu, Shenglong Deng, Xianglu Xiao, Guang Yang, Kristina Xiao Liang

**Affiliations:** ^1^ Department of Biomedicine (IBM) University of Bergen Bergen 5009 Norway; ^2^ State Key Laboratory of Oral Diseases National Clinical Research Center for Oral Diseases West China School of Stomatology Sichuan University Chengdu 610041 China; ^3^ Department of Head and Neck Cancer Surgery West China Hospital of Stomatology Sichuan University Chengdu 610041 China; ^4^ NorVita Med AS Bergen 5161 Norway; ^5^ Bioengineering Department and Imperial‐X, Imperial College London, London W12 7SL, UK/6. National Heart and Lung Institute, Imperial College London, London SW7 2AZ, UK/7. Cardiovascular Research Centre, Royal Brompton Hospital, London SW3 6NP, UK/8. School of Biomedical Engineering & Imaging Sciences, King's College London, London WC2R 2LS, UK; ^6^ Bergen Center of Medical Stem Cell Research Institute of Medicine and Dentistry University of Bergen Bergen 5009 Norway

**Keywords:** cortical organoids, iPSCs, mitochondrial dysfunction, neurogenesis impairment, *POLG*‐related disorders

## Abstract

Mitochondrial dysfunction and impaired neurogenesis are central to mitochondrial DNA polymerase (*POLG*)‐related disorders, yet therapeutic options remain limited. Here, patient‐derived induced pluripotent stem cell (iPSC)‐based cortical organoids are used to model *POLG*‐associated neurodegeneration and assess the therapeutic potential of metformin. Single‐cell RNA‐seq reveals distinct vulnerabilities in dopaminergic, glutamatergic, and GABAergic neuronal subtypes, with dopaminergic neurons exhibiting the most severe loss and mitochondrial transcriptomic deficits. Metformin treatment (250 µm, 2 months) significantly restores neuronal identity, subtype‐specific gene expression, and mitochondrial function. Functional assays demonstrate improved mitochondrial membrane potential (TMRE), increased mitochondrial mass (MTG, MTDR), and reduced oxidative stress (MitoSOX, BAX/cleaved caspase 3). Notably, mitochondrial DNA (mtDNA) copy number and the expression of mitochondrial replisome proteins (*POLG*, *POLG*2) are upregulated, indicating enhanced mitochondrial genome maintenance. Calcium measurement confirms improved neuronal excitability. Untargeted metabolomics further reveals metformin‐induced metabolic reprogramming, including enrichment of the tricarboxylic acid (TCA) cycle, amino acid metabolism, and redox‐related pathways. Together, these findings demonstrate that metformin enhances mitochondrial integrity and neural function across multiple neuronal subtypes and offer mechanistic insights into its potential as a treatment for *POLG*‐related disorders.

## Graphical abstract

Patient‐derived *POLG*‐mutant cortical organoids reveal neuronal subtype‐specific mitochondrial and synaptic defects, with dopaminergic neurons most affected. Metformin treatment restores neuronal identity, mitochondrial function, and excitability, increased mtDNA maintenance, and reprogrammed metabolism via TCA and redox pathways. These results highlight metformin's potential to treat *POLG*‐related neurodegeneration through mitochondrial repair and functional restoration.

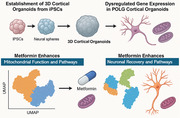

## 1 Introduction

DNA polymerase γ (Pol γ) is the main replicative polymerase for mitochondrial DNA (mtDNA) and also plays essential roles in multiple mtDNA repair pathways.^[^ ^1^ ^]^ The human *POLG* and *POLG2* genes are located on chromosomes 15q26.1 and 17q24.1, respectively, and encode the catalytic and accessory subunits of Pol γ. As a holoenzyme, Pol γ consists of the *POLG* catalytic subunit, which carries out DNA polymerization, and the *POLG*2 accessory subunit, which enhances processivity. This enzyme plays a central role in maintaining mitochondrial genome integrity and function. Pol γ is essential for the synthesis of mtDNA during mitochondrial biogenesis, and for correcting damage to mtDNA that accumulates due to metabolic activity and oxidative stress.^[^ ^1^ ^]^ Given that mitochondria house their own genome, accurate replication and repair are crucial for sustaining energy production and overall cellular homeostasis, particularly in energy‐demanding tissues such as the brain and muscles.^[^ ^1^, ^2^, ^3^ ^]^

Mitochondrial dysfunction is a critical factor in the pathogenesis of *POLG*‐related diseases, with profound effects on neuronal health. Mutations in the *POLG* gene disrupt mtDNA replication and repair, leading to impaired oxidative phosphorylation, reduced adenosine triphosphate (ATP) production, and increased oxidative stress.^[^ ^1^, ^2^ ^]^ These deficiencies are particularly harmful to neurons, which have high energy demands, resulting in progressive neuronal degeneration.^[^ ^3^ ^]^ This mitochondrial failure drives the neurodegenerative symptoms seen in *POLG*‐related disorders, such as spinocerebellar ataxia, myopathy, and peripheral neuropathy, presenting a significant challenge for treatment.^[^ ^4^, ^5^ ^]^

Recent advances in disease modeling, such as the development of induced pluripotent stem cell (iPSC)‐derived cortical organoid, offer new opportunities to study the complex mechanisms of neurodegenerative diseases in a controlled, 3D environment.^[^ ^6^, ^7^ ^]^ These organoids provide a valuable platform to investigate neuronal development, synaptic function, and mitochondrial dynamics in the context of *POLG* mutations.^[^ ^8^ ^]^ By modeling these processes, iPSC‐derived cortical organoids hold promise for advancing our understanding of disease mechanisms and for developing targeted therapies to address the mitochondrial impairments that drive neurodegeneration in *POLG*‐related disorders.^[^ ^9^ ^]^

Among the potential therapeutic agents, metformin—a well‐established treatment for type 2 diabetes—has gained considerable attention for its neuroprotective effects. In addition to targeting key pathways such as mitochondrial metabolism and insulin signaling, growing evidence suggests that metformin can counteract neurodegenerative diseases by promoting neurogenesis, synaptic plasticity, and mitochondrial health.^[^ ^10^, ^11^ ^]^ Mechanistically, metformin activates AMP‐activated protein kinase (AMPK), which subsequently influences downstream effectors including the mammalian target of rapamycin (mTOR) and sirtuin 3 (SIRT3) pathways, leading to enhanced mitochondrial biogenesis, improved oxidative phosphorylation efficiency, and reduced reactive oxygen species (ROS) production.^[^ ^12^, ^13^ ^]^ Furthermore, metformin has been shown to modulate mitochondrial dynamics by promoting fusion and mitophagy, thereby preserving mitochondrial quality and metabolic flexibility. These effects position metformin as a promising candidate for treating mitochondrial‐mediated diseases, yet the precise molecular pathways through which it influences neuronal mitochondrial metabolism remain underexplored in the context of *POLG*‐related disorders.

Building upon our previous work, which characterized the effects of *POLG* mutations in iPSC‐derived 3D cortical organoids,^[^ ^14^ ^]^ we investigated the cellular and molecular alterations in specific neuronal subpopulations and evaluated their responses to therapeutic intervention. *POLG*‐related disorders, caused by impaired mtDNA replication and repair, result in significant neuronal dysfunction characterized by reduced ATP production, increased oxidative stress, and disrupted synaptic signaling. Previous studies,^[^ ^15^, ^16^ ^]^ have demonstrated the molecular and functional diversity of neuronal subtypes by using single‐cell RNA sequencing (scRNA‐seq) approaches to identify subtle differences in gene expression that underlie their specific roles in synaptic transmission and plasticity. In this study, we extended our analysis to explore changes within distinct neuronal subpopulations in *POLG*‐mutant organoids and their responses to metformin treatment. Specifically, we focused on dopaminergic, GABAergic, and glutamatergic neurons, analyzing the effects of *POLG* mutations on their neuronal function, mitochondrial health, and synaptic signaling. Using scRNA‐seq we investigated the cluster‐specific alterations in these neuronal subtypes and evaluated how metformin treatment modulated these changes and metformin's impact on enriched pathways related to neurogenesis, synaptic transmission, and mitochondrial metabolism.

## 2 Results

### 2.1 Establishment of 3D Cortical Organoids Model from iPSCs

We generated iPSCs from skin fibroblasts of one patient with *POLG* mutations and two neurologically healthy individual controls, as described in our previous studies.^[^ ^14^, ^17^ ^]^ The patient carried compound heterozygous c.1399 *G* > *A*, p.A467T and c.2243 *G* > *C*, p.W748S (CP2A). The patient exhibited specific symptoms of *POLG*‐related disorders, including progressive spinocerebellar ataxia, extraocular myopathy, migraine‐like headaches, and peripheral neuropathy.^[^ ^18^ ^]^

**Figure 1:**
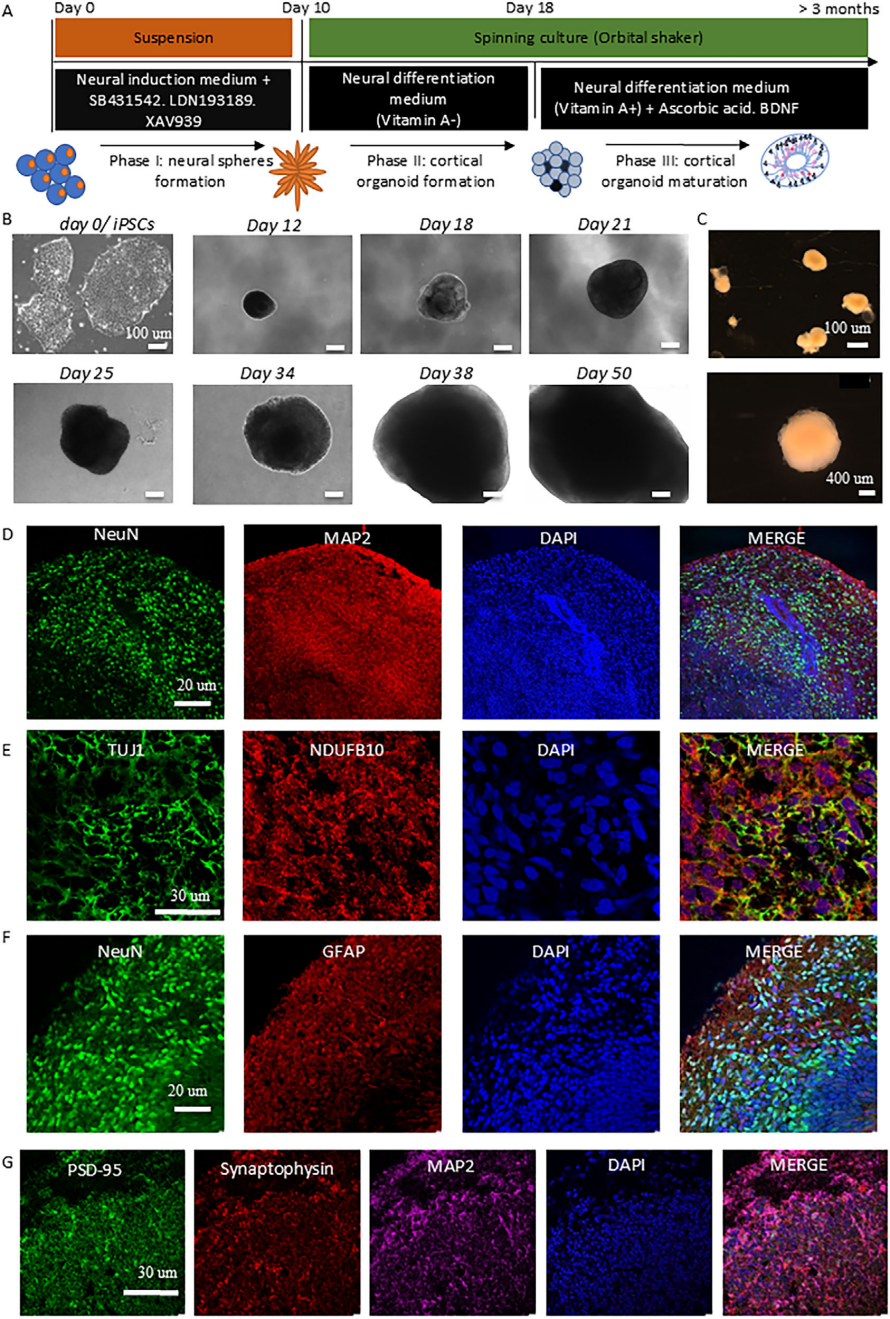
Generation of cortical organoids from iPSCs. A) The process of differentiation consists of three phases: Neural sphere formation (Phase I), a combination of the approach of dual SMAD inhibition and canonical Wnt inhibition, neural sphere was achieved by generating embryoid bodies in stationary suspension 3D culture. Cortical organoid formation (Phase II), transferring the cells with an orbital shaker and culturing them in neural differentiation medium without vitamin A to promote regionalization factors and cortical organization. Cortical organoid maturation (Phase III), maintaining the organoids in neural differentiation medium supplemented with vitamin A, BDNF, and ascorbic acid for long‐term neural maturation. B) Representative phase contrast images were captured at various time points during the differentiation process, including day 0, day 12, 18, 21, 25, 34, 38, and 50. The scale bar represents 100 µm. C) The morphology of organoids on day 50 of differentiation was examined. The scale bar represents 100 and 400 µm. D,E) Representative immunofluorescent imaging was performed on the cortical organoid on day 30 of differentiation from the control iPSCs. The staining revealed the presence of the neural marker TUJ1 and NeuN, mature neural marker MAP2, and mitochondrial complex I marker NDUFB10. Nuclei were stained with DAPI (blue). The scale bar represents 20 and 30 µm respectively. F) Representative immunofluorescent imaging was performed on the cortical organoid on day 90 of differentiation from the control iPSCs. The staining revealed the presence of the neural marker NeuN and astrocyte marker GFAP. Nuclei were stained with DAPI (blue). The scale bar represents 20 and 30 µm respectively. G) Representative immunofluorescent imaging was performed on the cortical organoid on day 90 of differentiation from the control iPSCs. The staining revealed the presence of the pre‐synaptic marker synaptophysin, the post‐synaptic marker PSD‐95, and the mature neural marker MAP2. Nuclei were stained with DAPI (blue). The scale bar represents 30 µm.

We further differentiated the *POLG* patient iPSCs and the control to 3D cortical organoids using our previously reported protocol,^[^ ^14^ ^]^ through three key stages: neural sphere formation, cortical organoid development, and maturation (**Figure** **1**A). The differentiation process of iPSC‐derived cortical organoids was monitored over time, demonstrating progressive morphological development and structural complexity (Figure 1B; Figure S2, Supporting Information). Initially, iPSC colonies exhibited compact and tightly clustered morphologies (Day 0). To monitor the morphogenesis of cortical organoids, we performed phase‐contrast imaging during the differentiation. The organoids showed a progressive increase in size and structural complexity over time, transitioning from compact neuroepithelial spheres to more organized and multilayered structures (Figure S1A, Supporting Information). By day 18–22, organoids began to exhibit clear edges and internal stratification. From day 37 onward, a distinct outer rim and dense core became apparent, indicative of continued neural maturation (Figure S1B, Supporting Information). Immunofluorescence staining at early stages confirmed robust expression of the neural progenitor marker NESTIN throughout the organoid structure (Figure S1C, Supporting Information), validating successful neural induction. Together, these results demonstrate the reproducibility and temporal progression of cortical organoid development, forming a reliable model system for downstream analysis. By day 50, we had generated large, complex organoids (Figure 1C).

At the early stage (day 30), we observed the expression of neural marker TUJ1, NeuN, and mature neuronal marker MAP2 mitochondrial complex I marker NADH oxidoreductase subunit B10 (NDUFB10) (Figure 1D,E). At the late stage (day 90), we observed the expression of neural marker NeuN, astrocyte marker GFAP, presynaptic marker synaptophysin, postsynaptic marker PSD‐95, and mature neural marker MAP2 (Figure 1D,E).

Overall, our results demonstrate that iPSC‐derived cortical organoids from *POLG* patients recapitulate key aspects of human neurodevelopment, including temporal progression, cellular diversity, and mitochondrial marker expression. This provides a reliable and physiologically relevant 3D model for investigating *POLG*‐associated neuropathology and mitochondrial dysfunction.

### 2.2 Metformin Restores Mitochondrial Function and Neuronal Integrity in *POLG* Brain Organoids

Building upon our recent findings that *POLG*‐mutant cortical organoids exhibit impaired neural differentiation, with expansion of stress‐associated progenitors, suppressed neurogenesis, and transcriptional signatures of oxidative stress and mitochondrial dysfunction, we previously demonstrated that metformin significantly improved mitochondrial membrane potential, ATP output, and mtDNA copy number. Mechanistically, metformin activated AMPK and SIRT3, inhibited mTOR, enhanced mitochondrial biogenesis and fusion (e.g., PGC‐1α, OPA1), and promoted mitophagy (LC3B, BNIP3).^[^ ^19^, ^20^ ^]^

**Figure 2:**
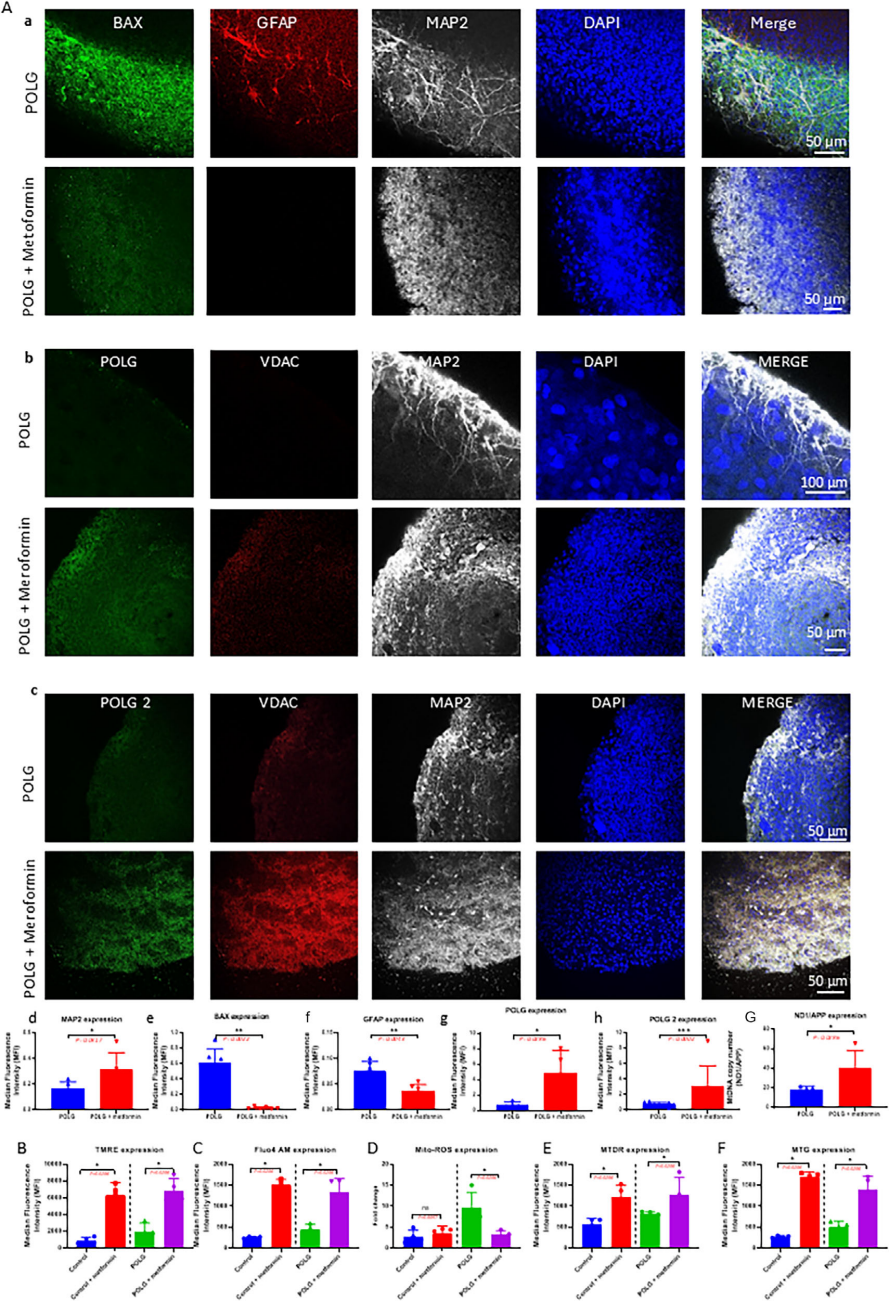
Mitochondrial function, marker expression, and mtDNA copy number measurement in cortical organoid derived from *POLG* patient iPSCs compared to untreated controls. A) Representative immunofluorescence images and quantitative analysis showing expression of mitochondrial markers and stress‐related proteins in cortical organoid derived from control and *POLG*‐mutant iPSCs. a) BAX (green), GFAP (red), MAP2 (white), and DAPI (blue) staining in control and POLG organoids, indicating increased apoptosis (BAX) and astrogliosis (GFAP) in *POLG* samples. b,c) POLG (green, b), POLG 2 (green, c), VDAC (red), MAP2 (white), and DAPI (blue) staining of cortical organoid from POLG patient lines and POLG‐treated metformin. Merge images show co‐localization and tissue architecture. d–h) Quantification of immunofluorescence signal intensity for MAP2 (d), BAX (e), GFAP (f), POLG (g), and POLG 2 (h) across different groups. Four areas were measured for each sample. Data are shown as mean ± SD. *p < 0.05; **p < 0.01; ***p < 0.001; statistical significance was determined using the Mann–Whitney *U*‐test. B–F) Flow cytometry analysis of mitochondrial function, including TMRE (B), Fluo‐4 AM (C), MitoSOX (D), MTDR (E), and MTG (F). Each condition was analyzed with four biological replicates. Data are presented as mean ± SD. *p < 0.05; statistical significance was determined using the Mann–Whitney *U*‐test. G) Relative mtDNA copy number by RT‐qPCR analysis using ND1 and APP‐Values are presented as Log2 of the ratio between the expression values of ND1 in relation to APP. Data are presented as mean ± SD. *p < 0.05; statistical significance was determined using the Mann–Whitney *U*‐test. Data are presented as mean ± SD from *n* = 4 biological replicates unless otherwise stated.

To evaluate mitochondrial function, neuronal integrity, and cellular stress in the *POLG*‐derived cortical organoid, we performed immunofluorescence staining using key markers, including BAX and cleaved caspase 3 (apoptosis), GFAP (astrocytes), MAP2 (neurons), *POLG*, and VDAC (a mitochondrial outer membrane protein). *POLG* organoids treated with metformin exhibited markedly decreased BAX, cleaved caspase 3, and GFAP signals compared to untreated *POLG* organoids (**Figure** 2A–e,f and Figure S3, Supporting Information), suggesting reduced apoptosis and reactive astrogliosis. Correspondingly, MAP2 expression was increased, indicating enhanced neuronal regeneration (Figure 2A‐a). Additionally, *POLG* protein levels were elevated in both *POLG* and *POLG*2 organoids following metformin treatment (Figure 2A‐b,c,g,h), and VDAC signal intensity was higher in treated samples compared to untreated ones (Figure 2A‐b,c). Quantitative analysis (Figure 2A–d,e,f,g; Figure S3, Supporting Information) confirmed these findings, demonstrating significantly reduced BAX, cleaved caspase 3, and GFAP expression, along with increased MAP2 levels in metformin‐treated organoids. These results suggest that metformin reduces cellular stress, and supports neuronal survival in the *POLG* mutant cortical organoid.

To further evaluate mitochondrial function, we performed flow cytometry on cortical organoids using a panel of mitochondrial‐specific fluorescent probes. Comparisons were made between metformin‐treated and untreated samples in both control iPSC‐derived organoids and *POLG* patient‐derived organoids. In *POLG* organoids, metformin treatment led to a significant increase in TMRE fluorescence intensity (Figure 2B), indicating restoration of mitochondrial membrane potential. Fluo‐4 AM staining revealed decreased intracellular calcium levels post‐treatment (Figure 2C), suggesting improved calcium homeostasis. Additionally, metformin significantly reduced mitochondrial ROS, as reflected by a lower MitoSOX/MTG fluorescence ratio (Figure 2D). MitoTracker Green (MTG) and MitoTracker Deep Red (MTDR) signals—markers of mitochondrial mass—remained stable or slightly increased following treatment (Figure 2E,F), indicating preservation or enhancement of mitochondrial content despite prior dysfunction. In control organoids, metformin similarly increased TMRE, Fluo‐4 AM, MTG, and MTDR signals, but had no significant effect on MitoSOX levels (Figure 2B–F). We further investigated the effects of metformin on mtDNA maintenance. Using qPCR analysis targeting the ND1/APP ratio, we found that metformin treatment resulted in a significant increase in mtDNA copy number in *POLG* organoids (Figure 2G).

Collectively, these results demonstrate that metformin alleviates mitochondrial dysfunction and restores mtDNA copy number, reduces oxidative and calcium‐related cellular stress, and supports mitochondrial maintenance and neuronal recovery in *POLG* patient‐derived cortical organoids.

### 2.3 Diversity in Human Distinctive Neuronal Cluster

Recent scRNA‐seq studies have uncovered surprising diversity among spinal motor neurons (MN), identifying at least ten distinct molecular subtypes in mouse models.^[^ ^21^ ^]^ In our previous work,^[^ ^14^ ^]^ we distinguished various neuron types in control and *POLG* patient iPSC‐derived cortical organoids, including dopaminergic, GABAergic, glutamatergic neurons, and neural progenitor cells. In this study, we utilized the same dataset but delved deeper into identifying specific neuronal subtypes within each major group to better understand their roles and functional diversity.

To investigate cell‐type‐specific alterations in cortical organoids, we performed scRNA‐seq on three groups: control, *POLG* patient‐derived, and metformin‐treated *POLG* cortical organoids. After stringent quality control filtering and normalization, we conducted cell type annotation and isolated neuronal populations for in‐depth subtype analysis (Figure S2, Supporting Information). Leiden clustering and dimensionality reduction identified 13 neuronal clusters (labeled 0–12), which were subsequently merged into 9 refined groups (a–i) based on shared marker gene expression. These clusters were categorized into four major neuronal types: dopaminergic neurons (clusters a, b, d, h), glutamatergic neurons (clusters c, e), GABAergic neurons (cluster i), and neural progenitor cells (clusters f, g).

**Figure 3:**
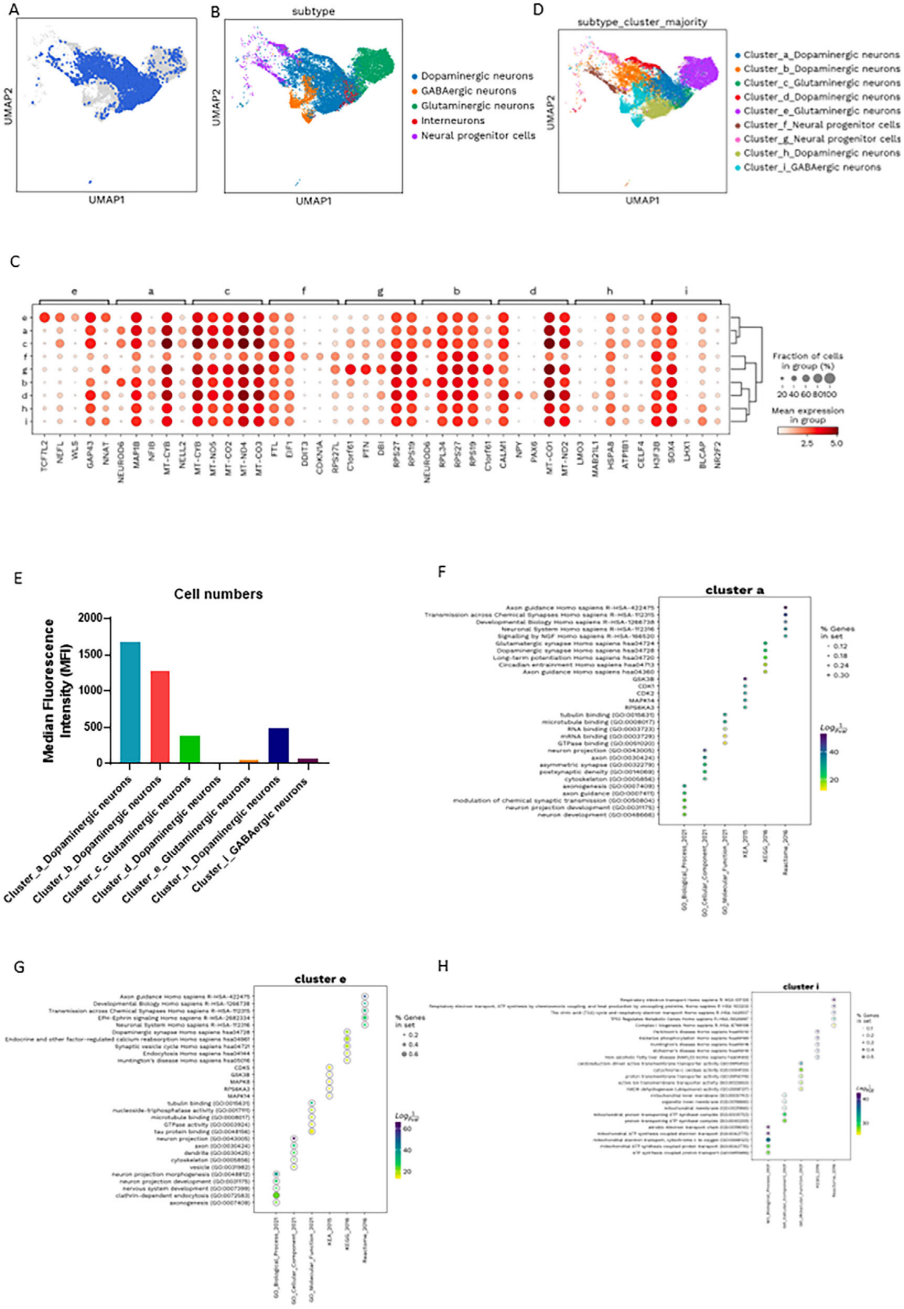
ScRNA‐seq reveals heterogeneity of cortical organoids‐derived neuronal cells. A) Neuronal cells in 3‐month‐old organoids derived from control iPSCs visualized using the UMAP algorithm. B) UMAP plot showing the distribution of single cells in 4 distinctive neuronal clusters (Dopaminergic neurons, blue, GABAergic neurons, yellow, Glutamatergic neurons, green, and interneurons, red) and special neural progenitor cells clusters (purple). C) Heatmap showing DEGs and manually selected markers in 9 neuron subclusters (a–i). Each dot represents a gene's expression profile within a specific neuronal cluster. Dot size corresponds to the percentage of cells expressing the gene (“in group %”), while color intensity indicates the average expression level (normalized expression). For visual clarity, only the top five DEGs per cluster are displayed, selected based on statistical significance (adjusted *p*‐value ≤ 0.05) and effect size (|log_2_ fold change| ≥ 1). Cluster a showed elevated expression of *NEUROD6, MAP1B, NFIB*, and *MT‐CYB1*, genes associated with neuronal differentiation, axonal development, and mitochondrial respiration. Cluster b was enriched for ribosomal genes (*RPL34, RPS27*, and *RPS19*) and calcium signaling regulator CALM1, indicating increased protein synthesis and signaling activity. Cluster c exhibited high expressions of mitochondrial genes (*MT‐CYB, MT‐ND5, MT‐CO2, MT‐ND4, MT‐CO3*, and *MT‐ATP6*), suggesting active oxidative phosphorylation and mitochondrial metabolism. Cluster d expressed *CALM1, MT‐CO1, MT‐ND2, NPY*, and *PAX6*, which are involved in mitochondrial function, neuropeptide signaling, and early neuronal patterning. Cluster e showed increased levels of *TCF7L2, NEFL, WLS, GAP43*, and *NNAT*, supporting roles in neuronal maturation, axon growth, and synaptic plasticity. Clusters f and g were enriched for *FTL, EIF1, DDIT3, CDKN1A*, and *RPS27L*, reflecting stress responses, cell cycle regulation, and translational activity during early neurodevelopment. Cluster i was characterized by high expression of *H3F3B, SOX4, LHX1, BLCAP*, and *NR2F2*, genes linked to transcriptional regulation, interneuron identity, and neuronal fate specification. D) UMAP plot showing neuronal subclusters after reintegration and clustering (Cluster_a_Dopaminergic a neurons, Cluster_b_Dopaminergic b neurons, Cluster_c_Glutamatergic c neurons, Cluster_d_Dopaminergic d neurons, Cluster_e_Glutamatergic e neurons, Cluster_f_Neural progenitor f cells, Cluster_g_Neural progenitor g cells, Cluster_h_Dopaminergic h neurons, Cluster_i_GABAergic I neurons). E) Number of cells in each subcluster in the cortical organoid. F–H)The enrichment analysis of GO, KEGG pathways, and Reactome pathways for DEGs in neurons from clusters a (G), e (H), and i (I). Data are presented as mean ± SD from *n* = 3 biological replicates unless otherwise stated. Statistical significance was determined using Mann–Whitney *U*‐test] with *α* = 0.05.

To investigate neuronal heterogeneity in the cortical organoids, we isolated the neuronal compartment, comprising 14606 cells (**Figure** **3**A). Within this population, we identified dopaminergic, GABAergic, glutamatergic neurons, and neural progenitor cells (Figure 3B). Using Harmony integration, we further analyzed the data to uncover distinct gene enrichment patterns and identified nine neuronal subclusters (clusters a–i) (Figure 3C). Subsequently, these nine subclusters were mapped onto the broader categories of dopaminergic, GABAergic, glutamatergic neurons, and neural progenitor cells (Figure 3D). Among them, clusters a, b, d, and h were aligned with dopaminergic neurons, while cluster i corresponded to GABAergic neurons. Clusters c and e were identified as glutamatergic neurons, and clusters f and g were associated with neural progenitor cells (Figure S7, Supporting Information).

The gene expression analysis (Figure 3C) identified distinct molecular profiles across neuronal clusters. Cluster a (dopaminergic neurons) exhibited high expressions of *NEUROD6*, *MAP1B*, *NFIB*, and *MT‐CYB*, while Cluster b (dopaminergic neurons) was enriched for *RPL34, RPS27, RPS19, C1orf61, and CALM1*. Cluster c (glutamatergic neurons) showed elevated expression of mitochondrial genes, including *MT‐CYB, MT‐ND5, MT‐CO2, MT‐ND4, MT‐CO3, and MT‐ATP6*. In Cluster d (dopaminergic neurons), *CALM1, MT‐CO1, MT‐ND2, NPY, and PAX6* were highly expressed, while Cluster e (glutamatergic neurons) demonstrated upregulation of *TCF7L2, NEFL*, *WLS*, *GAP43*, and *NNAT*. Neural progenitor cell clusters f and g showed enriched expressions of *FTL, EIF1, DDIT3, CDKN1A, RPS27L, C1orf61, PTN, DBI, RPS27*, and *RPS19*. Last, cluster i (GABAergic neurons) exhibited high levels of *H3F3B, SOX4, LHX1, BLCAP*, and *NR2F2*. These findings highlight the distinct transcriptional signatures within each neuronal subpopulation.

Next, we explored the molecular diversity of the 7 neuronal subclusters (excluding neuro progenitors) through Gene Ontology (GO), KEGG pathway analysis, Kinase Enrichment Analysis (KEA), and REACTOME pathways. Cluster a (Dopaminergic a neurons, Figure 3F) was enriched in genes related to axon guidance, transmission across chemical synapses, developmental biology, and pathways specific to glutamatergic and dopaminergic synapses, highlighting its roles in neural connectivity and signaling. Cluster b (Dopaminergic b neurons, Figure S8, Supporting Information) was enriched in transcriptional regulation (e.g., mRNA degradation, RNA/DNA binding, transcription factor activity), protein synthesis (e.g., ribosome biogenesis, rRNA processing, translation), and metabolism (e.g., glycolysis, pyruvate metabolism, amino acid biosynthesis). Key signaling pathways included p53, mTOR, HIF‐1, and FoxO, associated with apoptosis, neuronal differentiation, and hypoxia response. Disease‐related pathways (e.g., Alzheimer's, Parkinson's, Huntington's, cancer) and protein transport/processing (e.g., ER protein folding) were also prominent. Cluster c (glutamatergic neurons, Figure S9, Supporting Information) was enriched in mitochondrial function (e.g., ATP synthesis, electron transport chain, respiratory chain complex I assembly), signaling pathways (e.g., non‐canonical Wnt, Fc receptor, G2/M transition, hypoxia response), and immune response (e.g., antigen processing, neutrophil degranulation). Key neuronal functions included neuron projection, axon transport, and synapse structure, with major KEGG pathways involving oxidative phosphorylation, glycolysis, carbon metabolism, HIF‐1, FoxO, and mTOR signaling. Cluster d (Dopaminergic neurons, Figure S8, Supporting Information) was enriched in translation and ribosome activity (e.g., ribosome biogenesis, translation, rRNA processing, mRNA splicing), mitochondrial function (e.g., ATP synthesis, electron transport chain, mitochondrial membrane organization), and cellular metabolism (e.g., macromolecule biosynthesis, mRNA stability). Molecular functions included RNA binding, rRNA and mRNA binding, NADH dehydrogenase activity, and oxidoreductase activity. Cellular components spanned ribosomes, mitochondria, the ER, the nucleus, and the lysosome. Cluster e (glutamatergic neurons, Figure 3G) was enriched in neuronal development and differentiation (e.g., axonogenesis, neuron projection development), synaptic function (e.g., neurotransmitter secretion, synapse assembly), calcium signaling and homeostasis, and membrane/cytoskeleton organization, emphasizing roles in neural growth and connectivity. Cluster h (Dopaminergic neurons, Figure S8, Supporting Information) was enriched in protein synthesis and processing (e.g., translation, SRP‐dependent targeting, ribosome biogenesis), RNA processing (e.g., mRNA splicing, RNA degradation, stability regulation), and mitochondrial function (e.g., electron transport chain, ATP synthesis, membrane organization). Stress responses, DNA replication and repair, signal transduction (e.g., Toll‐like receptor, NF‐kappaB), and cell cycle/apoptosis regulation were significant. Neural functions included inhibitory synapse assembly, GABAergic transmission, and synaptic vesicle cycling. Cluster i (GABAergic neurons, Figure 3H) was enriched in metabolism (e.g., ATP synthesis, mitochondrial function), protein synthesis (e.g., translation, ER protein targeting), neuronal development (e.g., axon guidance, neuron differentiation), synaptic transmission (e.g., neurotransmitter secretion), immune responses (e.g., neuroinflammation), and physiological regulation (e.g., insulin secretion, muscle contraction). This comprehensive analysis highlights the functional diversity across neuronal subclusters, emphasizing unique roles in neural development, signaling, metabolism, and homeostasis.

**Figure 4:**
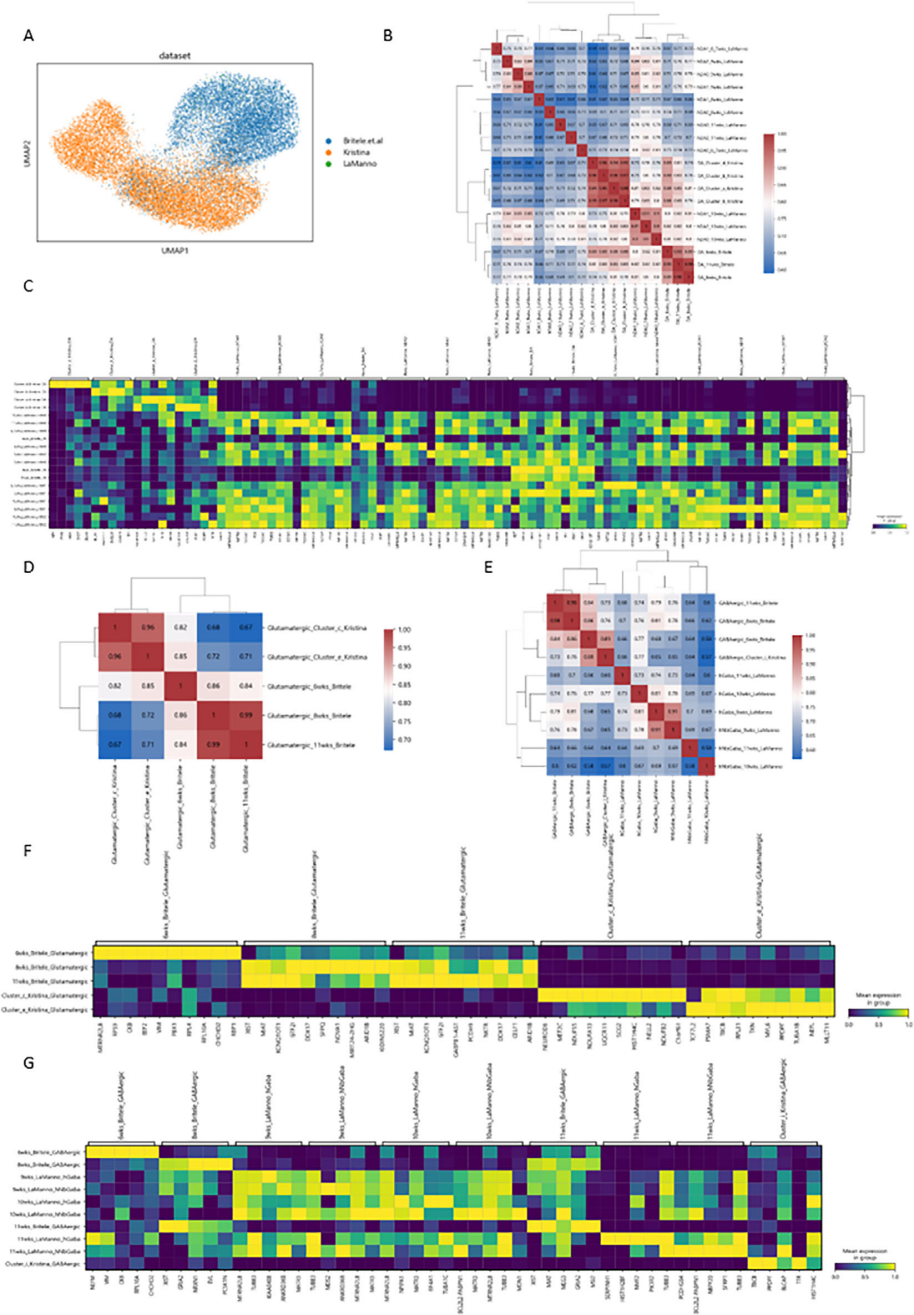
Single‐cell transcriptomic profiles of cortical organoids from the current dataset with those reported in the studies by La Manno et al. and Birtele et al. A) Cell type annotation of the current dataset, integrating reference annotations derived from the studies by La Manno et al. and Birtele et al. B,C) The heatmap of the correlation analysis (B) and gene expression (C) of dopaminergic neurons based on the mean expression levels of their genes. D,E) The heatmap of the correlation analysis (D) and gene expression (E) of glutamatergic neurons based on the mean expression levels of their genes. F,G) The heatmap of the correlation analysis (F) and gene expression (G) of GABAergic neurons based on the mean expression levels of their genes. Data are presented as mean ± SD from *n* = 3 biological replicates unless otherwise stated. Statistical significance was determined using Mann–Whitney *U*‐test with *α* = 0.05.

To evaluate the molecular identity of distinct neurons in cortical organoids compared to established human neuronal populations, we utilized two previously reported scRNA‐seq datasets of 6 to 11‐week post‐conception human embryos and their cultures from Birtele et al.^[^ ^15^ ^]^ and another from etal ventral midbrain (VM) DA populations from La Manno et al.^[^ ^16^ ^]^ Fetal and hPSC‐derived datasets were integrated, normalized, and clustered to generate a comprehensive gene expression matrix. Commonalities were visualized using UMAP (**Figure** **4**A), and correlation analysis was performed using the mean expression levels of genes. We observed a positive correlation for dopaminergic neurons across different developmental timepoints. Specifically, DA neurons (Figure 4B,C, Table S1, Supporting Information) derived from organoids displayed a strong correlation with DA neurons at 6 weeks from Birtele et al. and 10 weeks from La Manno et al., indicating that our organoid‐derived DA neurons share a similar molecular identity with established human midbrain DA neurons. For glutamatergic neurons (Figure 4D,E, Table S1, Supporting Information), organoid‐derived populations showed a high correlation with neurons at 6 weeks from Birtele et al.; however, no glutamatergic neurons were identified in the dataset from La Manno et al. Similarly, GABAergic neurons (Figure 4F,G, Table S1, Supporting Information) derived from organoids exhibited a strong correlation with GABAergic neurons at 6 weeks from Birtele et al. These findings demonstrate that the molecular profiles of neurons derived from our organoids closely resemble those of corresponding human fetal neuronal populations, validating the fidelity of organoid‐derived neurons to in vivo counterparts.

Analysis of mitochondrial gene expression across different neuronal subtypes in cortical organoids revealed distinct, cell‐type‐specific regulatory patterns (Figure S5, Supporting Information). Genes involved in mitochondrial fusion and fission dynamics (e.g., *MFN1/2, OPA1, DNM1L*) and protein import machinery (TOM/TIM complexes) displayed variable expression levels, reflecting differences in mitochondrial remodeling and import requirements among neuronal populations. Similarly, respiratory chain complex–associated genes (including assembly and auxiliary factors) and mtDNA replication/homeostasis regulators exhibited divergent expression patterns, with certain neuronal subtypes showing upregulation of key components (e.g., assembly factors for complex I and IV), while others demonstrated relative downregulation.

These data identified nine distinct neuronal subclusters in *POLG* iPSC‐derived cortical organoids, each characterized by unique transcriptional signatures and functional enrichments that closely mirror human fetal neuronal populations, thereby validating the organoids as a relevant model of neuronal diversity and disease. Moreover, the findings indicate that neuronal subtypes within cortical organoids harbor distinct mitochondrial transcriptional programs, likely reflecting differences in their energetic requirements, mitochondrial dynamics, and susceptibility to dysfunction.

### 2.4 Dysregulated Neuronal and Mitochondrial Gene Expression in Dopaminergic Neurons of *POLG* Cortical Organoid

**Figure 5:**
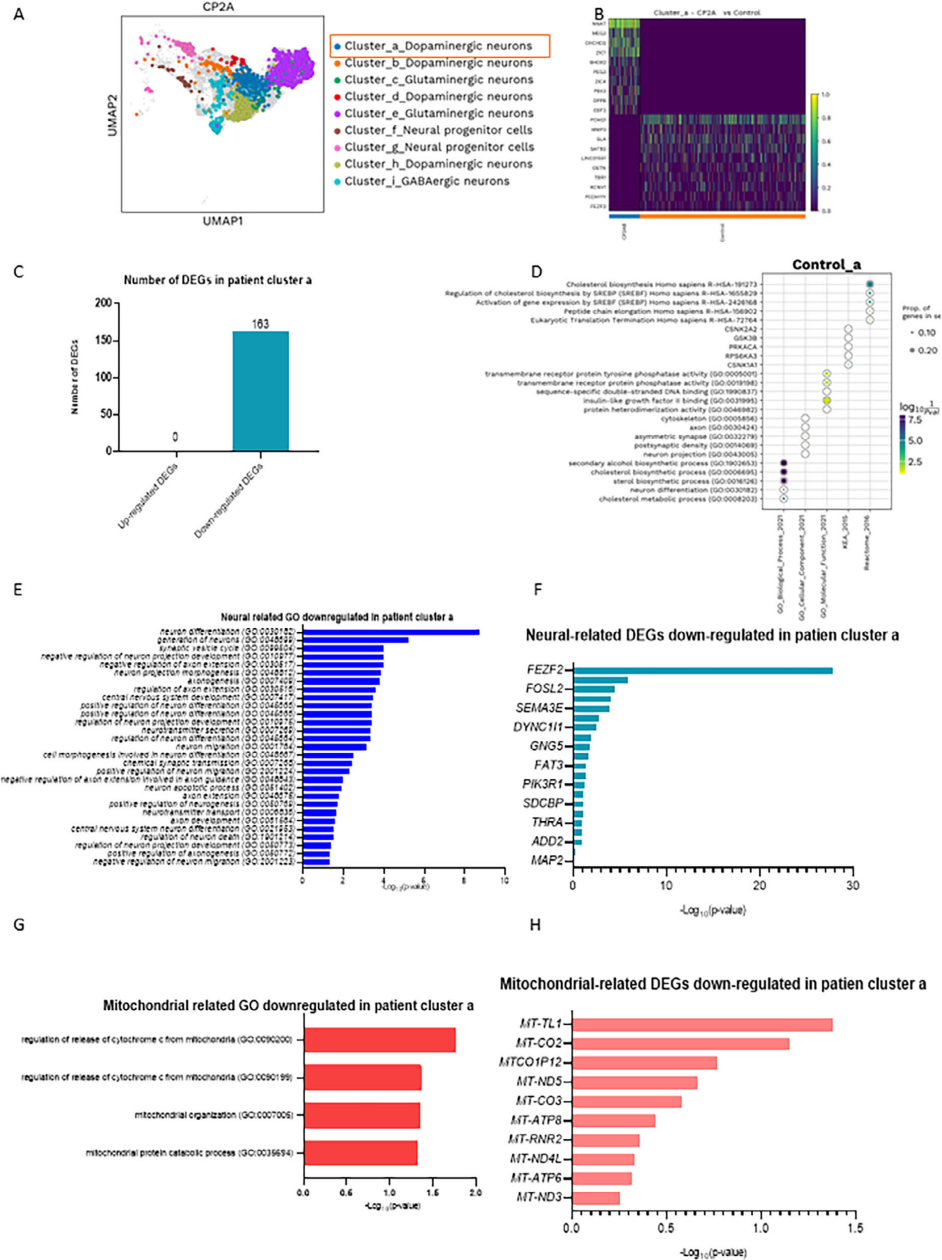
Single‐cell transcriptomic profiles of dopaminergic a neurons in the cortical organoid from *POLG* patients compared to controls. A) Cell clusters in 3‐month‐old organoids derived from patient iPSCs visualized using the UMAP algorithm. B) Heatmap illustrating the DEGs in dopaminergic a neurons of *POLG* patient CP2A cortical organoids compared to control cortical organoids. The heatmap displays the top DEGs (rows) across individual samples (columns), with yellow indicating higher expression and purple indicating lower expression. In the CP2A patient sample, dopaminergic a neurons, upregulated genes include *NNAT, MEG3, CHCHD2, ZIC1, SHOX2, PEG3, ZIC4, PBX3, DPP6*, and *EBF3*. These genes are associated with neuronal differentiation (*ZIC1, ZIC4, PBX3*, and *EBF3*), synaptic signaling (*DPP6*), and mitochondrial function (*CHCHD2*), as well as transcriptional regulation relevant to dopaminergic identity (*SHOX2, PEG3*). Downregulated genes include *FOXG1, MMP3, SLA, SATB2, LINC01551, OSTN, TBR1, KCNV1, PCDH11Y*, and *FEZF2*, many of which are essential for cortical development (*FOXG1, SATB2, TBR1*, and *FEZF2*), axonal projection (*PCDH11Y*), and synaptic plasticity (*KCNV1, OSTN*). C) The number of DEGs that are upregulated and downregulated in dopaminergic a neurons of patient CP2A cortical organoids compared to control cortical organoids. D) The enrichment analysis of GO pathways for downregulated DEGs in dopaminergic a neurons. E,F) Neural‐related GO pathways (E) and DEGs (F) for downregulated DEGs in dopaminergic a neurons. G,H) Mitochondrial‐related GO pathways (E) and DEGs (F) for downregulated DEGs in dopaminergic a neurons. Data are presented as mean ± SD from *n *= 3 biological replicates unless otherwise stated. Statistical significance was determined using Mann–Whitney *U*‐test with *α* = 0.05.

Our analysis identified seven distinct neuronal subtypes and two unique neural progenitor subclusters within the *POLG* cortical organoid (**Figure** **5**A). Comparisons between *POLG* patient‐derived organoids and controls revealed significant alterations in both cell proportions (Figure S10A, Table S2, Supporting Information) and cell numbers (Figure S10, Table S3, Supporting Information). Dopaminergic a neurons exhibited a dramatic decrease, dropping from 1679 cells (41.24%) in controls to 312 cells (15.02%) in the CP2A patient sample (Table S4, Supporting Information), highlighting their heightened vulnerability to *POLG* mutations. Similarly, dopaminergic b neurons showed a significant reduction, decreasing from 1267 cells (31.12%) in controls to 78 cells (3.76%) in the patient sample. In contrast, dopaminergic d neurons showed a slight increase, rising from 15 cells (0.37%) in controls to 23 cells (1.11%) in the patient sample, suggesting lower sensitivity to *POLG*‐induced dysfunction. Dopaminergic h neurons demonstrated increased resilience, with their proportion rising from 494 cells (12.13%) in controls to 379 cells (18.26%) in CP2A. Glutamatergic c neurons experienced a significant decline, dropping from 375 cells (9.21%) in controls to 40 cells (1.93%) in the patient sample, whereas glutamatergic e neurons showed a marked expansion, increasing from 55 cells (1.35%) in controls to 799 cells (38.49%) in CP2A, possibly due to compensatory differentiation mechanisms. GABAergic i neurons also expanded significantly, increasing from 71 cells (1.74%) in controls to 222 cells (8.96%) in the patient sample, suggesting developmental biases in the *POLG* mutation context. Among neural progenitors, progenitor f cells decreased from 98 cells in controls to 37 cells in the CP2A patient sample, while progenitor g cells increased markedly, rising from 17 cells in controls to 186 cells in the CP2A patient sample.

Further differential expression analysis across neuronal subtypes identified three key populations—dopaminergic a neurons, Glutamatergic e neurons, and GABAergic i neurons—that exhibited significant enrichment of DEGs related to neuronal function and mitochondrial pathways. Examination of the dopaminergic a neuron subcluster in *POLG* cortical organoid revealed substantial downregulation of key genes compared to control (Figure 5A,B). A total of 163 DEGs were identified in these neurons, all of which were downregulated (Figure 5C). The analysis of downregulated DEGs in dopaminergic a neuron subclusters of *POLG* cortical organoid compared to controls highlighted significant impairments in critical biological processes. GO enrichment (Figure 5D) revealed disruptions in mitochondrial pathways, including oxidative phosphorylation and the electron transport chain, indicating compromised energy metabolism. Additionally, pathways related to neuronal development, synaptic transmission, and axonogenesis were significantly downregulated, reflecting impaired neuronal integrity and function. Genes associated with cellular stress responses and dopamine metabolism are also suppressed, suggesting reduced capacity to manage oxidative stress and neurotransmitter activity. These findings emphasize the profound vulnerability of dopaminergic a neurons in *POLG* organoids, driven by a combination of mitochondrial dysfunction and impaired neuronal processes.

The GO analysis showed significant downregulation of neuronal processes critical for proper function and development in the *POLG* cortical organoid compared to controls (Figure 5E, Table S5, Supporting Information). Key pathways affected include synaptic signaling, neurotransmitter transport, and regulation of neuronal differentiation, highlighting disruptions in neuronal communication and network formation. Processes such as axon guidance and synaptic vesicle cycle were also markedly downregulated, suggesting deficits in neuronal connectivity and vesicle trafficking essential for neurotransmission. Furthermore, genes involved in neuronal migration, dendrite morphogenesis, and neuron projection development exhibited reduced expression, indicating impairments in structural maturation and neuronal architecture. These findings underscore the detrimental impact of *POLG* mutations on genes necessary for neuronal integrity, connectivity, and signaling, contributing to the observed dysfunction in the *POLG* cortical organoid.

We also found significant downregulation of key neuronal genes in patient cluster a, as shown in the data. Among these, *FEZF2*, a critical transcription factor involved in neuronal differentiation, exhibited the most pronounced reduction. Other significantly downregulated genes include *FOSL2*, associated with synaptic plasticity, and *SEMA3E*, which plays a role in axon guidance. Genes such as *DYNCI1*, involved in intracellular transport, and *MAP2*, a key marker of neuronal structure and dendritic stability, also showed reduced expression. Additionally, *PIK3R1*, linked to neurodevelopmental signaling, and *THRA*, associated with neuronal metabolism, were downregulated (Figure 5F, Table S6, Supporting Information).

Furthermore, the GO analysis of downregulated genes in the *POLG* cortical organoid further revealed disruptions in molecular functions related to mitochondrial activity and energy metabolism (Figure 5G, Table S7, Supporting Information). Specifically, there was a significant decrease in processes such as the regulation of the release of cytochrome c from mitochondria (GO:00 90200, GO:00 90199), indicating impairments in mitochondrial‐mediated apoptotic signaling. Additionally, pathways involved in mitochondrial organization (GO:0 007005) and the mitochondrial protein catabolic process (GO:00 34556) were downregulated, reflecting compromised mitochondrial structure, function, and protein turnover. These findings highlight the critical role of mitochondrial dysfunction in the pathophysiology of the *POLG* cortical organoid.

Additionally, we found significant downregulations in mitochondrial‐related genes in patient dopaminergic cluster a neurons (Figure 5H, Table S8, Supporting Information). Among these, *MT‐TL1*, a key gene involved in mitochondrial tRNA synthesis, exhibited the most substantial downregulation. Other critical genes significantly reduced include *MT‐CO2* and *MT‐CO3*, which are essential components of the mitochondrial electron transport chain complex IV, as well as *MT‐ND5* and *MT‐ND3*, which are integral subunits of complex I. Additionally, *MT‐ATP6* and *MT‐ATP8*, involved in ATP synthesis through complex V, showed notable downregulation. Furthermore, *MT‐RNR2* and *MTCO1P12*, important for mitochondrial ribosomal RNA and protein coding, were also significantly reduced. These findings underscore widespread mitochondrial dysfunction in the *POLG* cortical organoid, affecting critical pathways of energy production and mitochondrial integrity.

These findings highlight the profound impact of *POLG* mutations on neuronal subtypes and mitochondrial function. Dopaminergic a neurons demonstrated the greatest vulnerability, with widespread mitochondrial dysfunction and neuronal impairments.

### 2.5 Dysregulated Neuronal and Mitochondrial Gene Expression in Glutamatergic E Neurons of *POLG* Cortical Organoid

**Figure 6:**
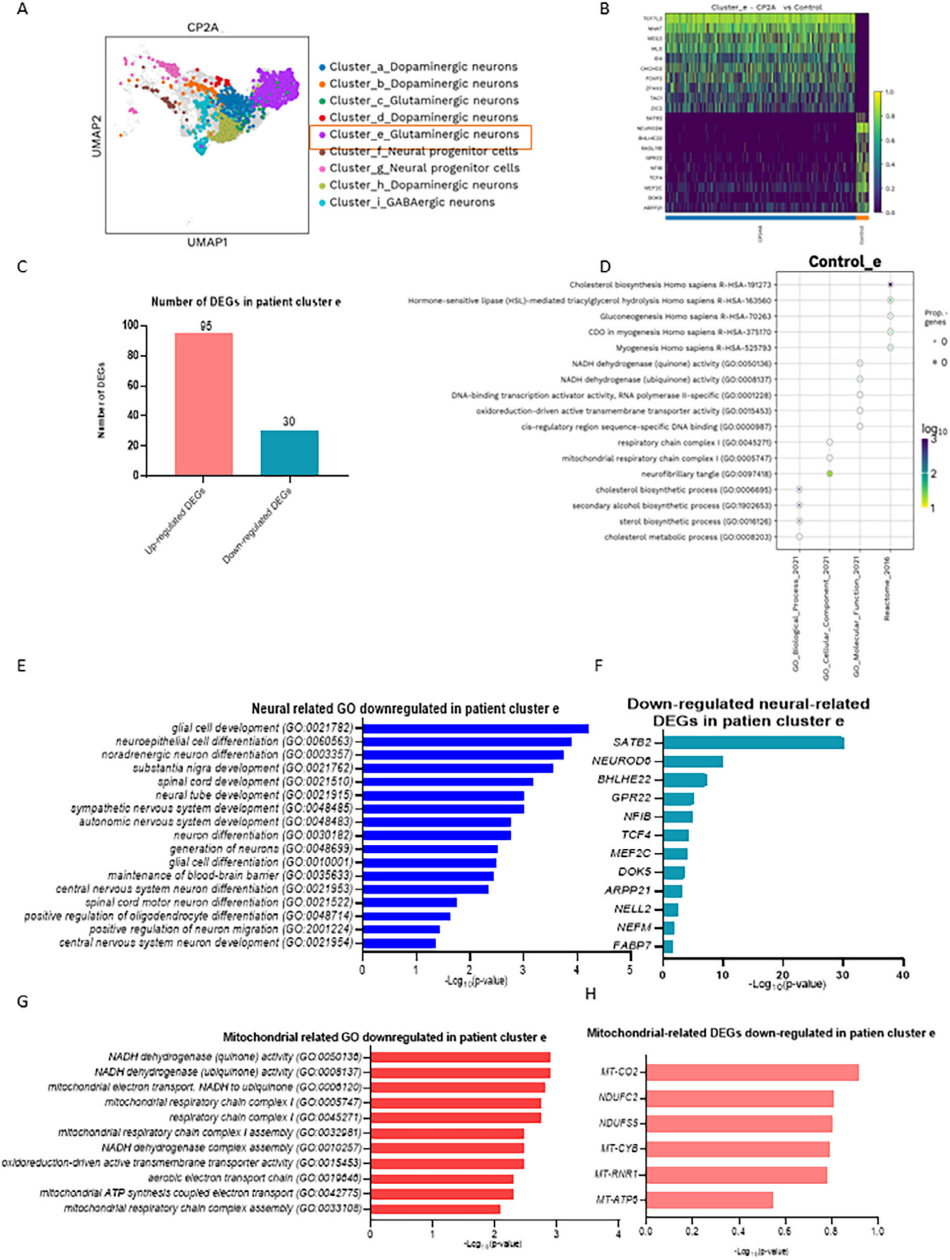
Single‐cell transcriptomic profiles of glutamatergic e neurons in the cortical organoid from *POLG* patients compared to control. A) Cell clusters in 3‐month‐old organoids derived from patient iPSCs visualized using the UMAP algorithm. B) Heatmap illustrates the DEGs in glutamatergic e neurons of *POLG* patient cortical organoids compared to control cortical organoids. The heatmap depicts the top DEGs (rows) across biological replicates (columns), with yellow indicating higher expression and purple indicating lower expression. Key upregulated genes in POLG organoids include *TCF7L2, NNAT, MEG3, WLS, ID4, CHCHD2, FOXP2, ZFHX3, TAC1*, and *ZIC2*. These genes are associated with transcriptional regulation of neuronal development (*TCF7L2, FOXP2, ZFHX3*, and *ZIC2*), synaptic signaling (*TAC1*), and mitochondrial homeostasis (*CHCHD2*). WLS participates in Wnt signaling, influencing both mitochondrial biogenesis and neuronal differentiation. Key downregulated genes include *SATB2, NEUROD6, BHLHE22, RASL11B, GPR22, NFIB, TCF4, MEF2C, DOK5*, and *ARPP21*. These genes are involved in cortical neuron maturation (*SATB2, NEUROD6*, and *MEF2C*), axonal guidance (*DOK5*), and activity‐dependent synaptic plasticity (*ARPP21*), with several (*TCF4, MEF2C*) also implicated in neuronal survival and mitochondrial regulation. C) The number of DEGs that are upregulated and downregulated in glutamatergic e neurons of patient CP2A cortical organoids compared to control cortical organoids. D) The enrichment analysis of GO pathways for downregulated DEGs in glutamatergic e neurons. E,F) Neural‐related GO pathways (E) and DEGs (F) for downregulated DEGs in glutamatergic e neurons. G,H) Mitochondrial related GO pathways (E) and DEGs (F) for downregulated DEGs in glutamatergic e neurons. Data are presented as mean ± SD from *n* = 3 biological replicates unless otherwise stated. Statistical significance was determined using Mann–Whitney *U*‐test with *α* = 0.05.

In the analysis of Glutamatergic e neurons (**Figure** **6**A) in *POLG* cortical organoid, a dramatic increase in their proportion was observed, rising from 1.35% in controls to 38.49% in patient organoids (Figure 6B, Figure S10, Supporting Information). Differential gene expression analysis identified a total of 125 DEGs, of which 95 genes were upregulated and 30 were downregulated in the *POLG* cortical organoid compared to controls (Figure 6C).

The enrichment analysis of downregulated DEGs revealed significant disruptions in key biological processes (Figure 6D). These included impairments in synaptic signaling and plasticity, which are critical for synapse organization and signal transmission, as well as deficiencies in neuronal development processes such as dendrite morphogenesis and neuron projection development, indicating compromised neuronal architecture and maturation. Downregulated DEGs were also enriched in pathways related to mitochondrial function, including mitochondrial organization and oxidative metabolism, highlighting the role of mitochondrial dysfunction in the *POLG* cortical organoid. Furthermore, pathways involved in neurotransmitter regulation, such as neurotransmitter release and the synaptic vesicle cycle, were significantly disrupted. These findings collectively emphasize the profound impact of downregulated DEGs on both neuronal and mitochondrial processes, contributing to the functional deficits observed in the *POLG* cortical organoid.

The analysis of downregulated neural‐related GO terms in patient cluster e revealed widespread impairments across critical neural development pathways (Figure 6E, Table S9, Supporting Information). Key processes such as glial cell development and neuroepithelial cell differentiation were significantly downregulated, suggesting disrupted development of support cells essential for neural function. Pathways like noradrenergic neuron differentiation, substantia nigra development, and spinal cord development pointed to deficits in neuronal differentiation and regional brain development. Additionally, processes such as sympathetic and autonomic nervous system development, neural tube development, and neuron migration were affected, indicating broad disruptions in early nervous system formation. Further downregulation was observed in pathways related to the maintenance of the blood–brain barrier, oligodendrocyte differentiation, and myelination, reflecting deficits in neural integrity, cellular organization, and synaptic connectivity.

Key neural‐related DEGs were significantly downregulated in patient cluster e (Figure 6F, Table S10, Supporting Information). Among these, *SATB2*, a transcription factor critical for cortical development and neuronal differentiation, showed the most substantial reduction. Other important downregulated genes included *NEUROD6*, which supports neuronal survival and differentiation, and *BHLHE22*, a regulator of neurogenesis. Additional downregulated genes, such as *GPR22*, involved in neural signaling, and *NFIB*, a transcription factor essential for neurodevelopment, further highlighted disruptions in neuronal pathways. Genes linked to synaptic plasticity (*TCF4*), synaptic maintenance (*MEF2C*), and neuronal signaling (*DOK5*) were also reduced, alongside structural markers like *NELL2*, *NEFM*, and *FABP7*, which are essential for neuronal architecture and function.

The analysis of downregulated mitochondrial‐related GO terms revealed significant disruptions in pathways essential for mitochondrial function and energy production (Figure 6G, Table S11, Supporting Information). Critical processes, such as NADH dehydrogenase (quinone) activity and mitochondrial electron transport from NADH to ubiquinone, were significantly downregulated, indicating compromised mitochondrial respiration and oxidative phosphorylation. Pathways related to the assembly of mitochondrial respiratory chain complexes, such as complex I and complex III, were also affected, reflecting structural and functional defects in the respiratory chain. Additional impairments were noted in pathways such as aerobic electron transport chain, oxidoreduction‐driven active transmembrane transport, and mitochondrial ATP synthesis coupled electron transport, all of which are critical for maintaining ATP production and cellular energy metabolism.

The mitochondrial‐related DEGs showed significant downregulation in patient cluster e (Figure 6H, Table S12, Supporting Information). Among these, *MT‐CO2*, a key component of mitochondrial complex IV in the electron transport chain, exhibited a notable reduction. Other critical downregulated genes included *NDUFC2* and *NDUFS5*, essential subunits of complex I, indicating deficits in NADH dehydrogenase activity. Additionally, *MT‐CYB*, a core component of complex III, and *MT‐RNR1*, which encodes mitochondrial ribosomal RNA, were significantly downregulated, highlighting disruptions in mitochondrial protein synthesis and respiratory function. Genes such as *MT‐ATP6*, involved in ATP production via complex V, also showed reduced expression, further underscoring deficits in mitochondrial energy metabolism.

These findings collectively highlight severe impairments in neuronal differentiation, connectivity, and mitochondrial function in Glutamatergic e neurons of the *POLG* cortical organoid. The observed disruptions in synaptic signaling, neuronal architecture, and energy production underscore the extensive impact of *POLG* mutations on neural and mitochondrial processes.

### 2.6 Altered Neuronal and Mitochondrial Regulation in GABAergic I Neurons of *POLG* Cortical Organoid

**Figure 7:**
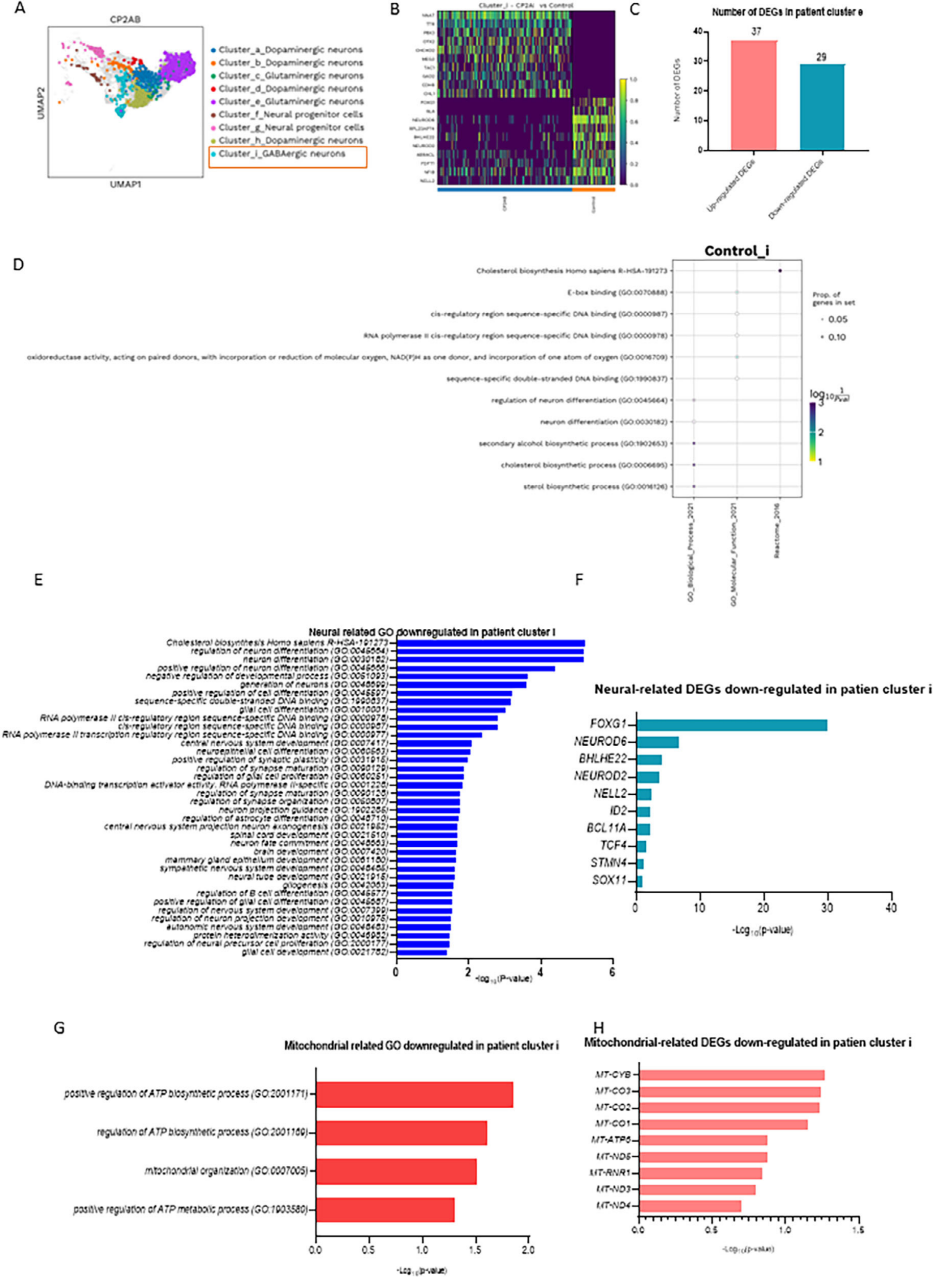
Single‐cell transcriptomic profiles of GABAergic i neurons in the cortical organoid from *POLG* patients compared to control. A) Cell clusters in 3‐month‐old organoids derived from patient iPSCs visualized using the UMAP algorithm. B) Heatmap illustrating the DEGs in GABAergic i neurons of POLG patient CP2A cortical organoids compared to control cortical organoids. The heatmap shows the top DEGs (rows) across samples (columns), with yellow representing higher expression and purple representing lower expression. In CP2A GABAergic neurons, upregulated genes include *NNAT, TTR, PBX3, OTX2, CHCHD2, MEG3, TAC1, GAD2, CDH8*, and *CHL1*. These genes are linked to GABAergic identity and neurotransmission (*GAD2, TAC1*), neuronal development and axonal guidance (*PBX3, OTX2, CHL1*, and *CDH8*), and mitochondrial function (*CHCHD2*). Downregulated genes include *FOXG1, SLA, NEUROD6, RPL23AP74, BHLHE22, NEUROD2, ABRACL, FDFT1, NFIB*, and *NELL2*, many of which are essential for cortical neuron differentiation (*FOXG1, NEUROD6*, and *NEUROD2*), transcriptional regulation (*BHLHE22, NFIB*), and synaptic maturation (*NELL2*). C) The number of DEGs that are upregulated and downregulated in GABAergic i neurons of patient CP2A cortical organoids compared to control cortical organoids. D) The enrichment analysis of GO pathways for downregulated DEGs in GABAergic i neurons. E,F) Neural‐related GO pathways (E) and DEGs (F) for downregulated DEGs in GABAergic i neurons. G,H) Mitochondrial‐related GO pathways (E) and DEGs (F) for downregulated DEGs in GABAergic i neurons. Data are presented as mean ± SD from *n* = 3 biological replicates unless otherwise stated. Statistical significance was determined using Mann–Whitney *U‐*test with *α* = 0.05.

In the GABAergic i neurons of the *POLG* cortical organoid (**Figure** **7**A), a heatmap comparison with controls (Figure 7B) revealed significant downregulation of key genes. Gene expression analysis identified 65 DEGs in patient‐derived organoids, with 37 upregulated and 29 downregulated genes (Figure 7C). Enrichment analysis of the downregulated DEGs uncovered disruptions in critical pathways related to synaptic signaling, neurotransmitter transport, and axonogenesis, which are essential for proper GABAergic neuronal function (Figure 7D). Moreover, processes such as dendrite morphogenesis and neuron projection development were significantly downregulated, indicating structural and connectivity deficits in these neurons.

The analysis of downregulated neural‐related GO terms in patient cluster i highlighted severe impairments across key biological processes (Figure 7E, Tables S13 and S14, Supporting Information). One of the most notable disruptions was in cholesterol biosynthesis, a pathway critical for maintaining neuronal membrane integrity and synaptic function. Other affected processes included the regulation of neuron differentiation, negative regulation of developmental processes, and sequence‐specific DNA binding, which are vital for neuronal development, gene expression, and functional specialization. Broader disruptions were observed in pathways such as positive regulation of cell differentiation, RNA polymerase II transcription regulatory activity, and central nervous system development, reflecting deficiencies in transcriptional activity and neuronal formation. Downregulation of processes associated with axonogenesis, synaptic signaling, and glial cell development further underscored significant deficits in structural and functional connectivity within neural networks.

Among the downregulated neural‐related DEGs in patient cluster i (Figure 7F, Table S15, Supporting Information), *FOXG1*, a key transcription factor essential for cortical development and neuronal differentiation, showed the most significant downregulation. Other critical genes, such as *NEUROD6* (involved in neuronal survival and differentiation) and *BHLHE22* (a neurogenesis regulator), were also significantly reduced. Genes linked to synaptic plasticity and axonal guidance, such as *NEUROD2* and *NELL2*, were notably downregulated, along with *ID2* (a regulator of neuronal differentiation) and *BCL11A* (crucial for neuronal development). Additional reductions in genes like *TCF4* (neuronal transcription regulation), *STMN4* (cytoskeletal organization), and *SOX11* (neurogenesis regulation) further highlighted impairments in neuronal development, differentiation, and synaptic function in the *POLG* cortical organoid.

The analysis of mitochondrial‐related GO terms downregulated in patient cluster i revealed significant impairments in pathways essential for energy production and mitochondrial function (Figure 7G, Table S16, Supporting Information). Key processes, such as positive regulation of ATP biosynthesis (GO:2 001 171) and mitochondrial organization (GO:0 007005), were significantly downregulated, reflecting disruptions in ATP production and mitochondrial structural integrity. Pathways like positive regulation of ATP metabolic processes (GO:1 903 580) were also affected, indicating reduced efficiency in ATP utilization and cellular metabolic activity. These findings underscore the profound impact of *POLG* mutations on mitochondrial energy regulation and organization, contributing to the observed cellular dysfunction in the *POLG* cortical organoid.

At the gene level, mitochondrial‐related DEGs further highlighted impairments in mitochondrial respiratory function (Figure 7H, Table S17, Supporting Information). Downregulated genes included *MT‐CYB*, a core component of complex III, and *MT‐CO1*, *MT‐CO2*, and *MT‐CO3*, which are critical for complex IV activity in the electron transport chain. Genes integral to ATP synthesis, such as *MT‐ATP6* and *MT‐ATP8* in complex V, were significantly reduced, pointing to severe deficits in mitochondrial energy production. Additionally, genes like *MT‐ND5*, *MT‐ND4*, and *MT‐RNR1*, essential for complex I activity and mitochondrial ribosomal RNA synthesis, were also downregulated.

Together, these findings illustrate widespread mitochondrial dysfunction, particularly in oxidative phosphorylation and ATP generation, contributing to the energy deficits and impaired cellular function observed in the GABAergic i neurons of the *POLG* cortical organoid.

### 2.7 Metformin Enhanced Neuronal Recovery and Mitochondrial Function in *POLG* Cortical Organoid in Cluster_I_Gabaergic E Neurons and Cluster_E_Glutamatergic E Neurons

In addition to primarily targeting pathways such as mitochondrial metabolism and insulin signaling, growing evidence has demonstrated metformin's key role in counteracting neurodegenerative diseases. ^22^We further investigated the therapeutic potential of Metformin using our established *POLG* cortical organoid model. Organoids were treated with 250 µm Metformin continuously for two months and compared to untreated controls. The two‐month treatment duration was selected to allow sufficient time for capturing cumulative metabolic, structural, and transcriptomic changes, particularly in light of the slow maturation dynamics inherent to 3D cortical organoid systems.

**Figure 8:**
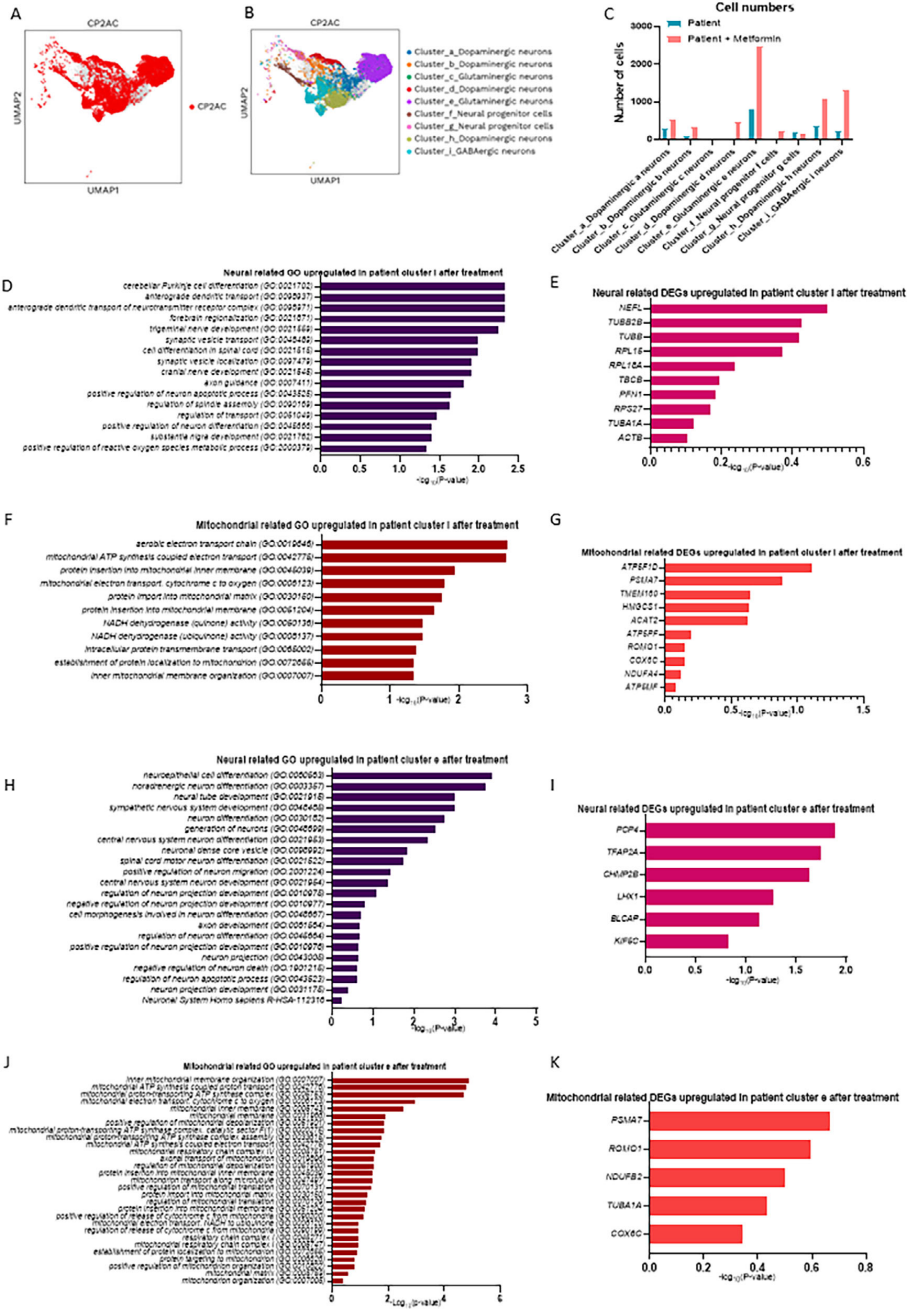
Single‐cell transcriptomic analysis of cortical organoid derived from *POLG* patients, comparing the profiles of organoids treated with metformin to those of untreated patient‐derived organoids. A) Neuron cells in 3‐month‐old organoids derived from patient iPSCs treated with Metformin visualized using the UMAP algorithm. B) Cell clusters in 3‐month‐old organoids derived from patient iPSCs treated with Metformin visualized using the UMAP algorithm. C) The number of neuronal clusters in CP2A patient cortical organoids derived from patient iPSCs, comparing those treated with Metformin to untreated controls. D,E) Neural‐related GO pathways (D) and DEGs (E) for upregulated DEGs after the Metformin treatment in GABAergic i neurons F,G) Mitochondrial‐related GO pathways (F) and DEGs (G) for downregulated DEGs in GABAergic i neurons. H,I) Neural‐related GO pathways (D) and DEGs (E) for upregulated DEGs after the Metformin treatment in Glutamatergic e neurons. J,K) Mitochondrial‐related GO pathways (F) and DEGs (G) for downregulated DEGs in Glutamatergic e neurons. Data are presented as mean ± SD from *n* = 3 biological replicates unless otherwise stated. Statistical significance was determined using Mann–Whitney *U*‐test with *α* = 0.05.

Following 2 months of treatment, different cell populations exhibited varying trends (**Figure** **8**A,B). Cluster_a_Dopaminergic a neurons, Cluster_b_Dopaminergic b neurons, Cluster_d_Dopaminergic d neurons, Cluster_e_Glutamatergic e neurons, Cluster_h_Dopaminergic h neurons, and Cluster_i_GABAergic i neurons all showed an increase in cell numbers (Figure 8C, Table S16, Supporting Information), suggesting that these populations responded positively to treatment and may play key roles in neuronal function recovery, particularly in synapse formation and mitochondrial restoration. In contrast, Cluster_c_Glutamatergic c neurons exhibited a decrease in cell numbers, indicating that these populations might have differentiated into other types or been less responsive to treatment. We further analyzed the neural and mitochondrial‐related GO terms and found that two subclusters, Cluster_e_Glutamatergic e neurons and Cluster_i_GABAergic i neurons, showed enriched neural and mitochondrial GO terms in the upregulated DEGs in treated versus untreated samples.

After Metformin treatment, significant upregulation of neural‐related GO terms was observed in Cluster i GABAergic i neurons, reflecting enhanced neural development, differentiation, and connectivity (Figure 8D, Table S18, Supporting Information). Key pathways included cerebellar Purkinje cell differentiation and forebrain regionalization, which indicate improved neuronal patterning and regional organization. Additionally, processes such as trigeminal nerve development, motor neuron axon guidance, and synaptic vesicle transport highlighted improvements in neuronal connectivity, circuit formation, and synaptic transmission. Enhanced processes like axon guidance and centrosome cycle regulation suggest better neuronal growth and structural organization, further supporting the notion that Metformin positively influences neuronal development and function in this cluster.

At the gene level, neural‐related DEGs upregulated in GABAergic i neurons after Metformin treatment showed significant enhancements in genes associated with neuronal structure and function (Figure 8E, Table S19, Supporting Information). Among these, *NEFL* exhibited the highest upregulation, reflecting improved axonal stability and signaling. Genes like *TUBB2B* and *TUBB3*, essential for microtubule organization and neuronal development, were significantly upregulated, indicating better cytoskeletal integrity. Additionally, *RPL16* and *RPL10A*, involved in ribosomal biogenesis and protein synthesis, showed increased expression, suggesting enhanced translational capacity. Other key genes, such as *TBCB* (microtubule dynamics regulator) and *PFN1* (actin polymerization), were elevated, further supporting neuronal growth and maintenance.

Metformin treatment also had a profound impact on mitochondrial function in GABAergic i neurons (Cluster i), as reflected by the upregulation of mitochondrial‐related GO terms (Figure 8F, Table S20, Supporting Information). Pathways such as aerobic electron transport chain, mitochondrial ATP synthesis coupled electron transport, and cytochrome c to oxygen transport indicated significant improvements in oxidative phosphorylation and energy metabolism. Processes like protein import into the mitochondrial matrix and inner mitochondrial membrane organization were also enhanced, highlighting better mitochondrial structural and functional organization. Additionally, pathways such as NADH dehydrogenase activity and positive regulation of mitochondrial membrane potential suggested improved mitochondrial efficiency and dynamics.

At the gene level, mitochondrial‐related DEGs in GABAergic i neurons showed notable upregulation (Figure 8G, Table S21, Supporting Information). Key genes included *ATP5F1D*, a critical component of ATP synthase in complex V, reflecting enhanced ATP production. Genes such as *PSMA7* (involved in protein turnover) and *TMEM70* (essential for ATP synthase assembly) were also prominently elevated. Other upregulated genes included *HIGD1A* (mitochondrial respiratory chain activity), *COX6C* (complex IV subunit), and *NDUFA4* (complex I subunit), all of which support enhanced electron transport chain function. Together, these findings underscore improved mitochondrial performance and energy metabolism following Metformin treatment.

In Cluster e Glutamatergic e neurons, metformin treatment led to significant enrichment of neural‐related GO terms associated with neuronal differentiation and development (Figure 8H, Table S22, Supporting Information). Key pathways included neuroepithelial cell differentiation, sympathetic nervous system development, and central nervous system neuron differentiation, reflecting enhanced neural progenitor activity and neuronal subtype specification. Processes such as axonogenesis, generation of neurons, and positive regulation of neurogenesis were also upregulated, suggesting improved neuronal connectivity, outgrowth, and structural maturation.

The analysis of neural‐related DEGs upregulated in patient Glutamatergic e neurons after treatment highlighted significant changes in gene expression associated with neuronal development and function (Figure 8I, Table S23, Supporting Information). Among the upregulated genes, *PCP4* exhibits the highest expression, indicating its pivotal role in neural recovery and connectivity. Similarly, *TFAP2A* and *CHL1/FBP2* are strongly upregulated, reflecting their involvement in neuronal differentiation and synaptic organization. Moderate upregulation of genes such as *LHX1* and *BLCAP* further supports enhanced neuronal stability and growth, while *KIF5C*, though less pronounced, underscores its importance in axonal transport.

Mitochondrial‐related GO terms in Glutamatergic e neurons also showed significant improvements after Metformin treatment (Figure 8J, Table S24, Supporting Information). Pathways such as inner mitochondrial membrane organization, mitochondrial ATP synthesis coupled electron transport, and protein import into the mitochondrial matrix were enriched, indicating enhanced mitochondrial structural integrity and functionality. Upregulated processes like positive regulation of mitochondrial membrane potential and establishment of protein localization to mitochondria further highlighted improved mitochondrial dynamics and energy efficiency.

At the gene level, mitochondrial DEGs in Glutamatergic e neurons exhibited notable upregulation after metformin treatment (Figure 8K, Table S25, Supporting Information). Genes like *PSMA7* and *ROMO1*, which are critical for mitochondrial quality control and indirectly influence complex I activity, showed significant increases. Other key genes, such as *NDUFB2* (complex I subunit) and *COX6C* (complex IV subunit), were also elevated, reflecting enhanced oxidative phosphorylation efficiency and energy production. In addition, *TUBA1A*, a major cytoskeletal gene essential for neuronal differentiation and axonal outgrowth, was significantly upregulated, further supporting improved neuronal development and connectivity. Collectively, these findings demonstrate that metformin treatment enhances mitochondrial function, neuronal differentiation, and connectivity across both GABAergic and glutamatergic neurons.

### 2.8 Validation of Neuronal Subtypes and *POLG*‐Associated Transcriptomic Alterations

To validate neuronal subtype annotations, a two‐stage K‐means clustering analysis was performed. In the first round, non‐neuronal populations—astrocytes, radial glial cells, melanocytes, ependymal cells, tanycytes, and fibroblast‐enriched clusters (clusters 0, 6, 9)—were identified and excluded (Figure S9, Supporting Information). The second round focused on neuronal populations and revealed five neuronal clusters based on marker gene expression (Figure S10D, Supporting Information): Cluster 0 (glutamatergic neurons), Cluster 1 and 2 (other neurons), Cluster 3 (GABAergic neurons), and Cluster 4 (dopaminergic neurons). Differential gene expression (DGE) analysis confirmed distinct molecular identities. *NEUROD6* was elevated in dopaminergic neurons, NR2F2 in GABAergic neurons, and *TCF7L2*, *NEFL*, and *WLS* in glutamatergic neurons (Figure S10D–F, Supporting Information). These signatures were consistent with scMRMA‐derived clusters (dopaminergic cluster a, GABAergic cluster i, and glutamatergic cluster e), providing cross‐method validation.

GO enrichment revealed functional distinctions: dopaminergic neurons were enriched in axon guidance and synaptic transmission (Figure S10H, Supporting Information), glutamatergic neurons in neurodevelopment, synaptic function, and energy metabolism (Figure S10I, Supporting Information), and GABAergic neurons in oxidative phosphorylation, protein synthesis, and inhibitory synaptic signaling (Figure S10J, Supporting Information). In the *POLG* condition, GABAergic neurons displayed downregulation of 99 genes, with suppressed ATP synthesis and nucleotide biosynthesis pathways (Figure S10G, Supporting Information), aligning with mitochondrial dysfunction identified in scMRMA cluster i. Following metformin treatment, 98 genes were upregulated in GABAergic neurons, associated with neuroepithelial cell differentiation and midbrain development (Figure S10H, Supporting Information). Similarly, glutamatergic neurons showed 103 upregulated and 499 downregulated genes, with enrichment in synaptic function and oxidative phosphorylation pathways (Figure S10I, Supporting Information), closely mirroring findings from scMRMA cluster e. Together, both approaches demonstrated consistency in identifying *POLG*‐induced dysfunction and metformin‐induced recovery at the transcriptional level.

### 2.9 Pseudotime Analysis Reveals Metformin‐Driven Rescue of Neuronal Differentiation

To explore dynamic lineage relationships, pseudotime trajectory analysis using scVelo was conducted across nine neuronal clusters, including dopaminergic neurons (a, b, d, h), glutamatergic neurons (c, e), neural progenitors (f, g), and GABAergic neurons (i) (Figure S11, Supporting Information). Latent time metrics reconstructed developmental trajectories, initiating from neural progenitor cluster f and progressing through cluster g, which exhibited a higher proportion of spliced transcripts, indicative of advanced progenitor status (Figure S11B–E, Supporting Information). Partition‐based graph abstraction (PAGA) confirmed coherent connectivity between cell states, mapping differentiation paths among neuronal subtypes (Figure S11B, Supporting Information).

In *POLG* patient‐derived organoids, cells accumulated at early pseudotime stages, particularly within GABAergic neurons (cluster i), suggesting impaired maturation (Figure S11D, Supporting Information). Metformin treatment reversed this developmental arrest, facilitating cellular progression toward more mature glutamatergic neurons (cluster e) and increasing their representation at the pseudotime trajectory endpoint (Figure S11E, Supporting Information). Unspliced transcript levels were consistently elevated in neural progenitor clusters f, GABAergic cluster i, and dopaminergic cluster h (Figure S11F–I, Supporting Information), indicating active transcription and transitional states. These findings suggest that metformin alleviates the developmental blockade imposed by *POLG*‐associated mitochondrial dysfunction, promoting neuronal differentiation from GABAergic to glutamatergic fates.

### 2.10 Metabolic Changes in *POLG* Patient‐Derived Cortical Organoids with Metformin Treatment

To assess the metabolic impact of metformin treatment in *POLG* patient‐derived cortical organoids, we performed untargeted metabolomic profiling. Principal component analysis (PCA) revealed a clear separation between untreated *POLG* and metformin‐treated *POLG* samples, indicating distinct metabolic signatures between the two conditions (Figure S12A, Supporting Information). Notably, metformin‐treated samples clustered tightly and were distinctly segregated from the *POLG* group along both PC1 and PC2 axes, reflecting treatment‐induced metabolic reprogramming. Hierarchical clustering and heatmap visualization further confirmed global metabolic shifts following metformin treatment (Figure S12B, Supporting Information). Differentially expressed metabolites were enriched in the metformin group (G2), with several clusters showing increased or decreased intensity relative to untreated *POLG* samples (G1). These results suggest that metformin treatment restores specific metabolic pathways disrupted by *POLG* mutations, potentially contributing to its observed neuroprotective effects.

To further explore the biological pathways impacted by metformin in *POLG* cortical organoids, we performed metabolite enrichment analysis. The enriched pathways (Figure S13A, Supporting Information) were predominantly related to amino acid metabolism and transport, including glycine, serine, threonine, and methionine metabolism, as well as general amino acid derivatives processing. Several transport‐related pathways were also significantly enriched, such as SLC‐mediated transmembrane transport, Na⁺/Cl^−^‐dependent neurotransmitter transport, and the transport of inorganic cations, bile salts, and organic acids. In addition, pathways associated with inborn errors of metabolism—such as Hartnup disorder (defective SLC6A19), 3‐phosphoglycerate dehydrogenase deficiency, and non‐ketotic hyperglycinemia—were upregulated, indicating restoration of disrupted metabolic processes. Transcriptional and translational pathways, as well as xenobiotic and small‐molecule transport, were also enhanced.

These findings suggest a strong effect of metformin on amino acid utilization and neuronal signaling. Further refinement of the top 25 enriched metabolic pathways (Figure S13B, Supporting Information) revealed that metformin treatment significantly influenced a broad spectrum of interconnected processes, including glycine and serine metabolism, methionine metabolism, phenylalanine and tyrosine metabolism, and homocysteine degradation—all of which are central to amino acid synthesis and neurotransmitter production. Additional enriched pathways included cysteine metabolism, ammonia recycling, alanine metabolism, propanoate metabolism, and catecholamine biosynthesis, reflecting enhanced nitrogen handling and neurotransmitter regulation. Metformin also modulated redox‐related and mitochondrial pathways such as glutathione metabolism, glutamine metabolism, betaine metabolism, arginine and proline metabolism, the urea cycle, and the citric acid cycle, suggesting improved antioxidant defense and mitochondrial energy production. Other enriched pathways included aspartate metabolism, pyruvate/aldehyde degradation, taurine and hypotaurine metabolism, ketone body metabolism, phosphatidylethanolamine biosynthesis, vitamin K metabolism, the glucose–alanine cycle, thyroid hormone synthesis, and the Warburg effect, highlighting a systemic metabolic reprogramming.

To elucidate the metabolic mechanisms underlying metformin's therapeutic effects, we performed untargeted metabolomics analysis comparing untreated and metformin‐treated *POLG* cortical organoids. A total of 154 metabolites were significantly upregulated and 178 downregulated following metformin treatment (Figure S14A, Supporting Information). Metformin‐treated organoids showed prominent enrichment of metabolites involved in energy metabolism (Figure S14B,C, Table S26, Supporting Information). Among these, 3‐deoxy‐D‐arabino‐heptulosonate‐7‐phosphate (DAHP), a precursor in the shikimate pathway, was elevated, suggesting enhanced biosynthesis of aromatic amino acids and neurotransmitter precursors. FADH, a key cofactor in mitochondrial oxidative phosphorylation, was also increased, indicating improved mitochondrial redox activity. Additionally, 2‐oxo‐4‐methylthiobutanoic acid, an intermediate in methionine metabolism, and alpha‐*D*‐glucose 6‐phosphate, a central glycolytic and pentose phosphate pathway metabolite, were elevated, reflecting enhanced energy production and NADPH‐mediated antioxidant defense.

Beyond energy metabolism, metformin treatment also significantly altered amino acid and nucleotide metabolism (Figure S15A,B, Table S27, Supporting Information). Upregulated amino acid–related metabolites included L‐methionine sulfoxide, a marker of oxidative stress, and O‐acetylserine, an intermediate in serine and cysteine biosynthesis. Increases in L‐arginine, N‐α‐acetyl‐L‐arginine, and L‐tyrosine suggested enhanced nitrogen metabolism and neurotransmitter biosynthesis. β‐alanine, involved in pyrimidine degradation and carnosine synthesis, was also elevated.

Metformin also upregulated several neuroprotective metabolites, supporting its role in neuronal resilience and recovery (Figure S16A,B, Table S29, Supporting Information). Notably, cholecalciferol (vitamin D_3_), nicotinamide (a precursor of NAD⁺), sparstolonin B (a plant‐derived antioxidant and anti‐inflammatory compound), and all‐trans‐retinoic acid (a key regulator of neuronal differentiation and survival) were all significantly increased in metformin‐treated organoids.

Furthermore, metformin treatment enhanced redox homeostasis & mitochondrial protection metabolites in POLG cortical organoids (Figure S17A,B, Supporting Information). Notably, cholecalciferol (vitamin D_3_), nicotinamide (a precursor of NAD⁺), sparstolonin B (a plant‐derived antioxidant and anti‐inflammatory compound), and all‐trans‐retinoic acid (a key regulator of neuronal differentiation and survival) were all significantly increased in metformin‐treated organoids.

In addition, metabolites associated with xenobiotic metabolism and detoxification were enriched following metformin treatment (Figure S18A,B, Table S30, Supporting Information). Elevated levels of N‐nitrosomethylethylamine (NMEA) and S‐diclofenac, substrates of cytochrome P450 and glutathione conjugation pathways, indicated activation of metabolic clearance processes. Similarly, increased abundance of coumarin and Mdz‐glucuronide (a glucuronidated metabolite of midazolam) pointed to enhanced phase I and phase II detoxification activities.

Overall, untargeted metabolomic profiling revealed that metformin treatment induced broad metabolic reprogramming in *POLG* patient‐derived cortical organoids, restoring disrupted pathways related to energy metabolism, amino acid utilization, and redox balance. These metabolic changes support a mechanistic basis for metformin's neuroprotective effects, highlighting its potential to modulate mitochondrial function and enhance neuronal resilience in *POLG*‐related disorders.

## 3 Discussion

In this study (Table S31, Supporting Information), we used patient‐derived iPSC‐based 3D cortical organoids to explore how *POLG* mutations impact neuronal development and mitochondrial function. Single‐cell analysis revealed distinct vulnerabilities among dopaminergic, glutamatergic, and GABAergic neurons, with dopaminergic neurons showing the most severe mitochondrial and synaptic deficits. Metformin treatment led to subtype‐specific recovery, partially restoring gene expression related to neurodevelopment, synaptic function, and mitochondrial activity. Complementary assays confirmed these transcriptomic changes: Metformin improved mitochondrial membrane potential (TMRE), mass (MTG, MTDR), reduced oxidative stress (MitoSOX, BAX/cleaved caspase 3), and increased mtDNA copy number and POLG/2 expression. Calcium measurement also showed enhanced neuronal activity. Metabolomic profiling further demonstrated that Metformin reprogrammed key pathways involved in energy metabolism, oxidative phosphorylation, and neuroprotection. Together, these findings highlight Metformin's potential to rescue mitochondrial and neuronal deficits in *POLG*‐related neurodegeneration. Although Metformin improved mitochondrial and metabolic readouts in both control and *POLG* organoids, the relative recovery was more pronounced in *POLG* neuronal subtypes, consistent with their greater baseline deficits. These findings suggest that Metformin exerts a general protective effect on neuronal mitochondria, with potential therapeutic implications for *POLG*‐related disorders where mitochondrial vulnerability is particularly severe.

Our work builds on previous studies demonstrating the capacity of iPSC‐derived cortical organoids to model human brain development^14^. Notably, Lancaster et al. (2013)^[^ ^6^ ^]^ were among the first to describe the generation of cerebral organoids with structural organization resembling human brain tissue. Similarly, Pasça et al. (2015)^[^ ^23^ ^]^ developed cerebral organoid systems to study early cortical patterning and neural network formation. While these foundational studies focused on normative development, our approach applies this technology to disease modeling, using patient‐specific iPSCs with *POLG* mutations. This builds upon the observations of previous studies,^[^ ^14^, ^24^ ^]^ who reported mitochondrial abnormalities in POLG‐derived neurons, by providing a 3D model that captures both the molecular and spatial complexity of brain tissue.

Mitochondrial function was significantly improved in Metformin‐treated *POLG* cortical organoids, as demonstrated by multiple complementary assays. We observed increased mitochondrial membrane potential and mass, indicated by elevated TMRE, MTG, and MTDR signals, suggesting that Metformin enhances mitochondrial bioenergetic capacity and biogenesis. In parallel, reduced MitoSOX staining, BAX, and cleaved caspase 3 expression indicate lowered oxidative stress and apoptotic burden, further supporting improved mitochondrial homeostasis. At the genomic level, metformin treatment led to a marked increase in mtDNA copy number, as measured by ND1/APP qPCR, reflecting restored mitochondrial genome integrity. This effect was accompanied by elevated expression of key mitochondrial replisome proteins, including POLG and POLG2, which are essential for mtDNA replication and maintenance. These data strongly support the notion that metformin enhances both the structural and functional aspects of mitochondrial biology in *POLG*‐mutant neural cells. Furthermore, calcium detection using Fluo‐4 AM revealed increased spontaneous calcium transients in metformin‐treated organoids, indicating improved neuronal excitability and synaptic function. Together, these findings provide a comprehensive view of how metformin restores mitochondrial fitness and neural activity in a patient‐derived disease model and highlight its potential as a therapeutic agent targeting mitochondrial dysfunction in *POLG*‐related neurodegeneration.

Using scRNA‐seq, we identified nine distinct neuronal subtypes within the organoids, mirroring fetal neuronal diversity. These included dopaminergic, glutamatergic, and GABAergic neurons, each displaying unique transcriptional signatures and mitochondrial gene expression profiles. The alignment of these subtypes with fetal brain cell identities, as shown by La Manno et al. (2016)^[^ ^16^ ^]^ and Birtele et al. (2020),^[^ ^15^ ^]^ confirms the fidelity of our organoid model. Moreover, the enrichment of mitochondrial genes such as *MT‐CYB*, *MT‐ND5*, and *MT‐CO2* in specific clusters provides new insights into how mitochondrial dysfunction affects neuronal subtype specification.

Pathway enrichment analysis further demonstrated that *POLG* mutations lead to significant impairments in mitochondrial energy metabolism, synaptic signaling, and neuronal development. For example, dopaminergic neuron subclusters exhibited downregulation of axon guidance and mTOR signaling pathways, consistent with findings by Lipton et al. (2014)^[^ ^25^ ^]^ linking these pathways to neurodevelopmental plasticity. Glutamatergic neurons displayed elevated glycolysis and oxidative phosphorylation signatures, in line with the metabolic demands described by Nicholls and Budd (2000),^[^ ^26^ ^]^ yet these were insufficient to compensate for synaptic and structural gene downregulation.

Our data indicates that *POLG* mutations induce subtype‐specific vulnerabilities, most notably in Dopaminergic a, Glutamatergic e, and GABAergic i neurons. Dopaminergic a neurons showed the most severe mitochondrial and developmental impairments, including reduced expression of *MT‐CO2*, *MT‐CO3*, and *MT‐ND5*, and downregulation of structural markers like *MAP2* and *FEZF*2. These findings are consistent with reports by Stenton and Prokisch (2020)^[^ ^27^ ^]^ and Tzoulis et al. (2006),^[^ ^18^ ^]^ highlighting the sensitivity of dopaminergic neurons to mitochondrial deficits.

Interestingly, Glutamatergic e neurons increased in proportion, possibly reflecting a compensatory expansion. However, this population also exhibited downregulation of genes linked to dendritic morphogenesis and synaptic function, suggesting limited functional benefits. Similarly, GABAergic i neurons retained their proportional representation but showed declines in key regulatory genes such as *FOXG1* and *NEUROD6*, as well as mitochondrial components like *MT‐CYB* and *MT‐ATP6*, aligning with results from Mariani et al. (2016).^[^ ^28^ ^]^

Glutamatergic e neurons showed increased proportions in *POLG* patient organoids, possibly reflecting compensatory mechanisms. However, downregulation of genes involved in synaptic signaling, dendritic morphogenesis, and mitochondrial organization suggests limited functional resilience, consistent with Lipton et al. (2014).^[^ ^25^ ^]^ GABAergic i neurons maintained their proportion but exhibited decreased expression of axonogenesis‐ and mitochondria‐related genes, including *FOXG1, NEUROD6, MT‐CYB*, and *MT‐ATP6*, echoing findings by et al. (2016). GO and pathway analyses revealed broad impairments in oxidative phosphorylation, ATP synthesis, neuronal differentiation, and synaptic function, aligning with Pham et al. (2018).^[^ ^29^ ^]^ Although some upregulated genes in Glutamatergic e and GABAergic i neurons may represent compensatory responses, they are unlikely to overcome the widespread deficits. This interplay between cellular compensation and vulnerability mirrors patterns described by La Manno et al. (2018).^[^ ^16^ ^]^

In our analysis, dopaminergic a neurons emerged as one of the most vulnerable subtypes in *POLG*‐derived cortical organoid, exhibiting transcriptomic signatures consistent with mitochondrial dysfunction, synaptic impairment, and structural degeneration. These findings are in line with our previous study,^[^ ^17^ ^]^ which demonstrated that *POLG* mutations lead to progressive mtDNA) depletion, resulting in impaired oxidative phosphorylation (OXPHOS) and ATP production, particularly in dopaminergic neurons. Given their extensive axonal arborization, high basal firing rate, and reliance on efficient energy metabolism, dopaminergic neurons are particularly susceptible to bioenergetic failure. In the current dataset, dopaminergic a neurons showed downregulation of genes associated with mitochondrial respiratory chain components (e.g., *MT‐CO1*, *MT‐ND2*, and *MT‐CYB*), as well as reduced expression of pathways involved in complex I assembly, electron transport, and ATP synthesis. These changes parallel the biochemical deficits previously reported in *POLG* patient brains and iPSC‐derived models.^[^ ^17^ ^]^ The impaired mitochondrial output likely contributes to downstream deficits in axon maintenance, synaptic vesicle cycling, and dopaminergic neurotransmission, which are hallmarks of motor dysfunction and fatigue commonly seen in *POLG*‐associated neurodegenerative syndromes. These results not only support the cell‐type–specific vulnerability of dopaminergic neurons in *POLG* disease but also reinforce the value of our organoid platform in modeling clinically relevant phenotypes and identifying mitochondrial targets for therapeutic intervention.

Metformin treatment led to substantial recovery across several neuronal populations, including marked increases in cell numbers within Dopaminergic a, Dopaminergic b, Dopaminergic d, Glutamatergic e, and GABAergic i neurons. While prior studies have reported metformin's general neuroprotective effects through mitochondrial and insulin signaling pathways,^[^ ^30^, ^31^ ^]^ our study offers a novel, high‐resolution view of its differential impact across specific neuronal subtypes within a patient‐derived cortical organoid. By integrating scRNA‐seq analysis, we identify for the first time that metformin selectively restores transcriptomic profiles and mitochondrial gene expression in the most vulnerable neuronal clusters (e.g., GABAergic i and Glutamatergic e), including upregulation of key pathways involved in oxidative phosphorylation, axonal development, and synaptic transmission. These data provide mechanistic insights into cell‐type–specific responses, revealing that metformin's restorative effects are not uniformly distributed but are instead tailored to distinct neuronal vulnerabilities associated with *POLG* mutations.

Metformin treatment significantly improved neuronal and mitochondrial function in the *POLG* patient‐derived cortical organoid. In particular, GABAergic i and Glutamatergic e neurons showed enhanced differentiation, synaptic connectivity, and resilience to oxidative stress. GABAergic i neurons exhibited upregulation of pathways related to synaptic vesicle transport, axon guidance, and oxidative phosphorylation, supported by increased expression of genes such as *NEFL*, *TUBB2B*, and *PFN*1. Similarly, Glutamatergic e neurons showed enrichment in neurogenesis, axonogenesis, and mitochondrial organization pathways, with elevated expression of *NDUFB2* and *COX6C*, indicating improved mitochondrial bioenergetics and structural maturation.

At the mitochondrial level, key pathways such as NADH dehydrogenase activity, ATP synthesis, and protein import into the mitochondrial matrix were upregulated across multiple neuronal subtypes. These findings align with prior study emphasizing the importance of mitochondrial energy metabolism in neuronal function and recovery.^[^ ^32^, ^33^ ^]^

Compared to mitochondrial‐targeted agents like Coenzyme Q10, nicotinamide riboside, and idebenone—which mainly act as antioxidants or electron transport cofactors. Metformin exhibits broader metabolic effects. It activates AMPK, enhances mitophagy, improves mitochondrial membrane potential, and reduces ROS.^19^, ^20^ ^]^ Our single‐cell transcriptomic data further reveal that Metformin reprograms mitochondrial and neurodevelopmental pathways in a cell‐type–specific manner.

Metformin treatment significantly enhanced neuronal differentiation, connectivity, and mitochondrial function in Glutamatergic e neurons within the *POLG* patient‐derived cortical organoid. Enrichment of neural GO terms such as neuroepithelial cell differentiation, axonogenesis, and neurogenesis indicates improved neural progenitor activity and structural maturation. Concurrently, upregulation of mitochondrial pathways, including ATP synthesis, coupled electron transport, and inner mitochondrial membrane organization—suggests enhanced energy metabolism and mitochondrial integrity. Elevated expression of genes such as *NDUFB2* and *COX6C* further supports increased oxidative phosphorylation and mitochondrial efficiency, reinforcing metformin's role in restoring neuronal bioenergetics and function in this population.

Compared to other mitochondrial‐targeted compounds—such as Coenzyme Q10, nicotinamide riboside, and idebenone—metformin exerts broader and more integrative effects. While the former primarily function as antioxidants or cofactors in the electron transport chain,^[^ ^34^, ^35^ ^]^ metformin modulates upstream pathways by activating AMP‐activated protein kinase (AMPK), promoting mitophagy, enhancing mitochondrial membrane potential, and reducing ROS production.^[^ ^36^ ^]^ Our scRNA‐seq–based analysis further reveals that Metformin reprograms both mitochondrial bioenergetics and neurodevelopmental pathways in a cell‐type–specific manner, supporting its role as a systems‐level modulator of neuronal recovery. These findings underscore metformin's strong therapeutic potential for *POLG*‐related neurodegeneration—a condition that currently lacks disease‐modifying treatments. By restoring mitochondrial energy metabolism and promoting neurogenesis in vulnerable neuronal subtypes such as dopaminergic a and GABAergic i neurons, Metformin demonstrates both efficacy and clinical translatability. Its well‐established safety profile in both pediatric and adult populations further supports its rapid repurpose.

Importantly, several of the DEGs identified in this study are directly relevant to the neurological symptoms commonly observed in *POLG* mutation carriers. For example, the downregulation of *NEFL, MAP1B*, and *GAP43*—genes essential for axonal stability, cytoskeletal organization, and synaptic plasticity—may underlie the progressive cognitive decline and neurodevelopmental delay frequently reported in *POLG*‐related encephalopathies. Similarly, decreased expression of mitochondrial genes such as *MT‐CO1, MT‐CYB*, and *MT‐ATP6* in dopaminergic subclusters points to impaired oxidative phosphorylation, which could contribute to parkinsonism, ataxia, and other movement disorders arising from energy failure in motor circuitry.

Moreover, the altered expression of transcription factors such as *PAX6, SOX4*, and *LHX1*, involved in neuronal fate determination and interneuron specification, suggests disrupted cortical inhibitory network development. This may contribute to seizure susceptibility, a hallmark clinical feature in many *POLG*‐related syndromes, including Alpers–Huttenlocher syndrome and progressive myoclonus epilepsy. The enrichment of stress‐ and apoptosis‐related genes in neural progenitor clusters (e.g., *DDIT3, CDKN1A*) further implies developmental instability that may contribute to cortical atrophy and neurodegeneration.

In support of the observed transcriptomic and functional rescue, untargeted metabolomic profiling further demonstrated that metformin treatment induced a broad metabolic reprogramming in *POLG* patient‐derived cortical organoids. Principal component and hierarchical clustering analyses revealed a clear shift in global metabolite signatures following treatment, suggesting that metformin effectively restores disrupted metabolic networks. Notably, metabolite set enrichment analysis identified significant upregulation of pathways related to amino acid metabolism (e.g., glycine, serine, methionine, and threonine), neurotransmitter transport, and mitochondrial processes including the TCA cycle and ketone body metabolism. These findings align with improved mitochondrial gene expression and neuronal differentiation at the single‐cell level. In particular, metformin treatment increased metabolites such as FADH (a key cofactor in oxidative phosphorylation), alpha‐*D*‐glucose 6‐phosphate (linked to glycolysis and NADPH production), and DAHP (a shikimate pathway intermediate involved in neurotransmitter precursor biosynthesis), indicating enhanced energy production and redox capacity. The elevation of neuroprotective metabolites, including nicotinamide, vitamin D_3_, and all‐trans‐retinoic acid, further supports metformin's capacity to promote neuronal resilience and maturation. Moreover, enrichment of detoxification‐related metabolites such as Mdz‐glucuronide and NMEA suggests activation of xenobiotic clearance pathways, potentially reducing oxidative damage and stress burden. Together, these metabolomic signatures provide mechanistic evidence that metformin not only improves mitochondrial function but also enhances metabolic flexibility and neuronal homeostasis in POLG‐deficient neural systems.

Together, these findings position metformin as a promising candidate for targeting both mitochondrial dysfunction and neurodevelopmental impairments in *POLG*‐related disorders. The use of patient‐derived cortical organoids in this study not only provides mechanistic insight into disease pathology but also offers a scalable, human‐relevant platform for stratified therapeutic testing. In addition to transcriptomic and phenotypic recovery, untargeted metabolomic profiling revealed that metformin treatment induced broad metabolic reprogramming in *POLG* organoids. Enrichment of pathways related to amino acid metabolism, the TCA cycle, redox regulation, and mitochondrial energy production strongly supports a systems‐level effect of metformin on cellular metabolism. Notably, elevated levels of neuroprotective and bioenergetically relevant metabolites—such as FADH, alpha‐*D*‐glucose 6‐phosphate, nicotinamide, and all‐trans‐retinoic acid—further reinforce its role in restoring mitochondrial efficiency and neuronal homeostasis.

### 3.1 Limitations and Future Directions

Despite the promising findings, several limitations of our study warrant further investigation. First, while our 3D cortical organoid model provides valuable insights into the cellular and molecular effects of metformin, it does not fully recapitulate the complexity of the human brain. Future studies using in vivo models are needed to confirm the therapeutic potential of metformin in *POLG*‐related diseases and to explore its long‐term efficacy and safety. Additionally, the exact molecular mechanisms through which metformin modulates mitochondrial function and neurogenesis in neurons remain unclear. Further research should aim to elucidate these mechanisms to optimize metformin‐based therapies for neurodegenerative conditions.

Furthermore, although metformin demonstrated positive effects in key neuronal populations, certain subpopulations, such as glutamatergic c neurons, showed a reduction in cell numbers after treatment, and dopaminergic a populations did not exhibit significant recovery in neural or mitochondrial function. Identifying the underlying mechanisms driving these differential responses is essential for optimizing treatments to enhance therapeutic benefits while minimizing potential adverse outcomes. Moreover, while our study employed Ca^2^⁺ imaging to assess neuronal activity, direct electrophysiological recordings would provide complementary functional evidence and further strengthen the characterization of metformin's effects on neuronal function.

## 4 Conclusion

This study demonstrates that metformin significantly enhances neuronal recovery and mitochondrial function in *POLG* patient‐derived cortical organoids. Through the promotion of neurogenesis, synaptic connectivity, and mitochondrial bioenergetics, metformin effectively counteracts key pathological features associated with *POLG*‐related neurodegeneration. Importantly, untargeted metabolomic profiling revealed that metformin induces widespread metabolic reprogramming, including restoration of energy metabolism, redox balance, amino acid utilization, and detoxification pathways. These metabolite‐level changes complement the transcriptomic and phenotypic improvements observed across vulnerable neuronal subtypes, providing strong mechanistic support for metformin's therapeutic effects. Together, these findings highlight the potential of metformin as a repurposed treatment for mitochondrial disorders and establish a robust human organoid platform for further therapeutic discovery. This work lays a critical foundation for future preclinical validation and clinical translation of metformin in precision medicine strategies targeting mitochondrial diseases.

## 5 Experimental Section

### Cortical Organoid Generation

Cortical organoids were generated following previously established protocols.^[^ ^14^ ^]^ Feeder‐free iPSCs were maintained in E8 medium for at least 7 days prior to differentiation. Once the iPSCs exhibited optimal morphology and reached ≈70% confluency, they were dissociated using Accutase (Sigma‐Aldrich, A6964‐100ML) in PBS for 10 min at 37 °C, followed by centrifugation at 300 × g for 3 min. A total of 9000 viable cells were seeded into 96‐well ultra‐low attachment tissue culture plates in 150 µL of neural induction medium (NIM) supplemented with 50 µm ROCK inhibitor (Millipore, SCM075). Directed differentiation was achieved using dual SMAD inhibitors and a canonical WNT inhibitor. After 24 h, embryoid bodies (EBs) were formed, and on Day 2, the medium was replaced with NIM containing 50 µm ROCK inhibitor. On Days 4, 6, and 8, 100 µL of the medium was replaced with 150 µL of fresh neural induction medium, this time without ROCK inhibitor. By Day 10, the organoids were transferred into 6‐well ultra‐low attachment tissue culture plates containing neural differentiation medium without vitamin A. The cultures were placed on an orbital shaker to promote organoid development and cortical organization. From Day 18 onward, the neural differentiation medium (NDM) was supplemented with B27 with vitamin A supplement (Invitrogen, 17 504 044) and BDNF (R&D Systems, 248‐BD.) to support long‐term maturation. The medium was subsequently refreshed every 3–4 days to maintain optimal growth conditions.

### Immunofluorescence Staining of Cortical Organoids

The organoids were transferred from the culture onto a Superfrost adhesion slide (Thermo Fisher Scientific, J1800AMNZ) using a 1 mL pipette with a cutting tip to facilitate intact transfer without disruption. The slide was then allowed to dry completely after excess medium was removed. The organoids were subsequently fixed with 4% EM grade paraformaldehyde (PFA, Thermo Fisher Scientific, 28 908) in 1× PBS for 30 min at room temperature. The PFA solution was aspirated, and the slide was washed twice with PBS. To prevent non‐specific antibody binding and enable cell permeabilization, a blocking buffer (Sigma‐Aldrich, G9023) and 0.1% (v/v) Triton X100 (Sigma‐Aldrich, 9036‐19‐5), containing 10% normal goat serum (Sigma‐Aldrich, 50197Z) and 0.1% Triton X100 in 1× PBS was added to the slide and incubated at room temperature for 2 h. Primary antibodies were prepared in blocking buffer at the appropriate concentrations and added to completely cover the samples on the slide. The samples were then incubated in the dark for 24 h at 4 °C. After incubation, the primary antibodies were aspirated, and the samples were washed with PBS through repeated rinsing for 2 h. Secondary antibodies, along with Hoechst 33 342 nuclear counterstain, prepared in blocking buffer solution, were added to the samples and incubated for 48 h in the dark at 4 °C.

The excess liquid was aspirated, and the samples were rinsed repeatedly with PBS. The samples were then kept in PBS containing 0.01% sodium azide and incubated overnight at 4 °C in the dark to prevent contamination. The following day, the solution was removed, and the samples were mounted by adding 20 µL of Fluoromount‐G mounting medium (Southern Biotech, 0100‐20), onto each sample and covering them with a 1.5 mm coverslip. The mounting medium was allowed to polymerize at room temperature in the dark for 24 h and then stored at −20 °C until imaging. Images were analyzed using the Leica TCS SP8 STED 3× (Leica Microsystems). The following primary antibodies were used for immunostaining: MAP2 (chicken,1:1000; Abcam, ab5392), β‐tubulin III (TUJ1) (mouse, 1:1000; Abcam, ab78078), NeuN (rabbit, 1:500, Cell Signaling Technology, 24 307), BAX (mouse, 1:500; Abcam, ab216494), Cleaved Caspase 3 (mouse, 1:200; Cell Signaling Technology, 9661), POLG (rabbit, 1:500, Abcam, ab128899), POLG 2 (rabbit, 1:500, Abcam, ab227902), GFAP (chicken, 1:200; Abcam, ab4674), VDAC1 (mouse, 1:500; Abcam, ab186321), NDUFB10 (rabbit, 1:1000, Abcam, ab196019), NESTIN (10c2) (mouse, 1:50 Santa Cruz Biotechnology, sc23927), PSD95(mouse, 1:1500, Abcam, ab2723), Synaptophysin (rabbit, 1:500, Abcam, ab32127). Alexa Fluor Dyes (Invitrogen) were used at a 1:800 dilution as secondary antibodies.

### Metformin Treatment of Cortical Organoids

For phenotype rescue experiments, cortical organoids were treated with 250 µm metformin (Sigma‐Aldrich, 317 240) starting on day 6 of cortical organoid differentiation to rescue the phenotypes. The culture medium was replaced every two days, and the treatment was continued for a total duration of two months. Untreated organoids were cultured under identical conditions.

### Measurement of Mitochondrial Membrane Potential using TMRE

To assess mitochondrial membrane potential (*ΔΨm*), cells were incubated with tetramethylrhodamine ethyl ester (TMRE, Thermo Fisher Scientific, T669) at a final concentration of 100 nm in culture medium at 37 °C for 20 min, protected from light. Carbonyl cyanide 4‐(trifluoromethoxy) phenylhydrazone (FCCP, 10 µm) was used as a positive control to dissipate membrane potential. After incubation, cells were washed with pre‐warmed PBS and resuspended in FACS buffer (PBS containing 0.2% BSA). Samples were immediately analyzed using a BD Accuri C6 flow cytometer. TMRE fluorescence was detected in the PE or PE‐Texas Red channel (excitation: 549 nm, emission: 575–610 nm), and data were processed using BD Accuri C6 software.

### Mitochondrial Mass Measurement using MitoTracker Green (MTG) and MitoTracker Deep Red (MTDR)

Mitochondrial content was assessed using two potential‐independent dyes: MitoTracker Green FM (MTG, Thermo Fisher Scientific, M7514) and MitoTracker Deep Red FM (MTDR, Thermo Fisher Scientific, M22426). Cells were incubated with either 100 nm MTG or 100 nm MTDR in complete culture medium at 37 °C for 30 min. For MTDR staining, incubation was performed in the dark to prevent photobleaching. After staining, cells were washed with PBS and resuspended in FACS buffer (PBS with 0.2% BSA) for flow cytometry analysis. MTG fluorescence was detected on the FITC channel (excitation: 490 nm, emission: 516 nm), while MTDR fluorescence was detected on the APC channel (excitation: 644 nm, emission: 665 nm). Both dyes accumulated in mitochondria independent of membrane potential, making them suitable for quantifying mitochondrial mass and for normalization of functional mitochondrial markers.

### Detection of Mitochondrial Superoxide Using MitoSOX Red

Mitochondrial superoxide production was detected using MitoSOX Red (Thermo Fisher Scientific, M36008). Cells were incubated with 5 µm MitoSOX in Hank's Balanced Salt Solution (HBSS) at 37 °C for 10 min, protected from light. After staining, cells were washed twice with HBSS and resuspended in FACS buffer. MitoSOX fluorescence was analyzed in the PE channel (excitation: 510 nm, emission: 580 nm). Increased fluorescence indicated elevated mitochondrial ROS. MitoSOX Red signal was used to normalize other mitochondrial markers to mitochondrial mass.

### Measurement of Cytosolic Calcium Using Fluo‐4 AM

Intracellular calcium levels were measured using Fluo‐4 AM (Thermo Fisher Scientific, F14201). Cells were loaded with 5 µm Fluo‐4 AM in HBSS at 37 °C for 30 min, protected from light. Following incubation, cells were washed and incubated for an additional 10–15 min at 37 °C to allow complete de‐esterification. After washing, cells were resuspended in FACS buffer and analyzed immediately. Fluo‐4 fluorescence was detected in the FITC channel (excitation: 494 nm, emission: 516 nm).

### MtDNA Quantification Assessment

Genomic DNA was extracted using the DNeasy Blood and Tissue Kit (QIAGEN, 69 504) following the manufacturer's instructions. MtDNA copy number and depletion were assessed by quantitative real‐time PCR (RT‐qPCR), using *ND1* as the mitochondrial target and APP as the nuclear reference gene, as previously described.^[^ ^17^ ^]^

### ScRNA‐Seq And Data Analysis—Organoid Dissociation and Single Cell Isolation

The organoids were harvested by removing them from the culture medium and rinsing with 1× PBS (Invitrogen, 10010‐23). Ophthalmic scissors were used to cut the organoids into 1–2 mm fragments. The tissue pieces were then digested in 2 mL of Cell Live Tissue Dissociation Solution (Singleron Biotechnologies, 1 190 062) at 37 °C for 15 min, with continuous agitation on a thermal shaker within a 15‐mL centrifuge tube (Sarstedt, 62.5544.003). The progress of dissociation was periodically monitored under a light microscope. Following digestion, the cell suspension was filtered using a 40‐µm sterile cell strainer (Greiner, 542 040). The filtered cells were centrifuged at 350 × g for 5 min at 4 °C, and the resulting pellets were resuspended in 1 mL of PBS. Cell viability and concentration were assessed by staining the cells with 0.4% w/v Trypan Blue (Gibco, 15250‐061) and counting them using a hemocytometer under a light microscope.

### ScRNA‐Seq Library Preparation and Sequencing

ScRNA‐seq libraries were constructed using the GEXSCOPE single cell RNA‐seq Library Kit (Singleron Biotechnologies, 4 161 031) as per the manufacturer's instructions. The cell suspension was diluted to a concentration of 3 × 10⁵ cells mL^−1^ in PBS and loaded onto a microfluidic chip capable of capturing 6000 cells. Paramagnetic beads conjugated with oligo probes containing unique molecular identifiers and barcodes were then added to the cell suspension. Following cell lysis, the polyadenylated mRNA was bound to the beads, reverse‐transcribed into cDNA, and amplified via PCR. The resulting cDNA was fragmented, and Illumina‐indexed adapters were ligated to the fragments. Finally, the library was analyzed for fragment size distribution using an Agilent Fragment Analyzer.

### Library Sequencing

The library concentrations were measured using the Qubit 4.0 fluorometer, and the libraries were then combined in equimolar proportions. The sequencing was conducted on an Illumina NovaSeq 6000 platform, employing a paired‐end 2 × 150 bp strategy, which yielded a final depth of 90 GB per library. The demultiplexing process was performed using the multiplexing index on Illumina's Base Cloud platform.

### Transcriptome Data Pre‐Processing

The scRNA‐seq raw data were processed using the CeleScope software (v.1.3.0; www.github.com/singleron‐RD/CeleScope; Singleron Biotechnologies GmbH). Low‐quality reads were removed, and the remaining reads were aligned to the human reference genome using the STAR aligner (https://github.com/alexdobin/STAR). Gene annotations were derived from Ensembl 92, and the read‐to‐gene assignments were carried out using featureCounts (https://subread.sourceforge.net/), resulting in a UMI‐based gene count matrix for individual cells.

### Quality Control and Filtering

The gene count matrix was processed using the Python‐based scanpy package. Various quality control metrics were calculated, including the number of detected genes per cell (nFeature_RNA) and the percentage of mitochondrial unique molecular identifiers. Cells with a mitochondrial UMI (percent_mt) content exceeding 20% were considered non‐viable and excluded from further analysis. Additionally, potential doublets with more than 5000 detected genes and cell debris with fewer than 200 detected genes were also removed from the dataset.

### Sample Integration, Dimensionality Reduction, and Clustering

The three datasets were combined using the concatenate function from the data package in Python. Cell counts were normalized to 10 000 per cell, and highly variable genes were identified using dispersion‐based methods with thresholds of mean expression between 0.1 and 8 and dispersion above 0.5. Principal component analysis was conducted, retaining the top 17 principal components based on explained variance. Scanpy's neighbors function was used to compute a neighborhood graph, with parameters set to 20 neighbors and 17 PCs. The UMAP algorithm was employed to calculate reduced dimensions, using a minimum distance of 0.5, a spread scale of 1, and 200 iterations. Cell clusters were identified using the Leiden algorithm^[^ ^37^ ^]^ with a resolution of 0.5.

### Cell Type Annotation

Dimensionality reduction and unsupervised clustering were further refined using the scMRMA (https://github.com/JiaLiVUMC/scMRMA) tool to perform multi‐resolution marker‐based annotation. Cell type marker genes were identified by querying the PanglaoDB database^[^ ^38^ ^]^ (https://panglaodb.se/), and significance was assessed through Fisher's enrichment analysis based on the 20 nearest neighbors.

### Differential Gene Expression (DEG) Analysis

DEGs were identified using Scanpy's rank_genes_groups function, applying the Wilcoxon rank‐sum test with Bonferroni correction to control for multiple testing. Genes with adjusted *p* < 0.05 were considered significant. Only genes with consistent expression across clusters and log2 fold‐change > 0.25 were retained for further interpretation.

### Gene Enrichment Analysis

Pathway and process annotations were derived using the gseapy package in Python, which interrogated databases such as GO_Biological_Process_2021, GO_Molecular_Function_2021, GO_Cellular_Component_2021, Reactome_2016, KEGG_2016, and KEA_2015. Enrichment significance was determined using Benjamini–Hochberg false discovery rate (FDR) correction, and only terms with FDR‐adjusted *p*‐values < 0.05 were considered enriched.

### Batch Effect Correction and Dataset Integration

To compare the dataset with publicly available human fetal ventral midbrain scRNA‐seq data, raw counts were integrated using Harmony for batch correction. PCA embeddings from the top 2000 HVGs were computed and adjusted using Harmony to remove technical variability across datasets.

Cluster annotations for the Birtele dataset were inferred using Seurat (LogNormalize, RunPCA, FindClusters), with marker genes for known cell types including radial glia, neuroblasts, dopaminergic, GABAergic, and glutamatergic neurons. Subsequent integration enabled cross‐study correlation analysis, using Pearson correlation coefficients of average expression profiles stratified by cell type and time point.

### Neuronal Cell Subsetting and Clustering

Neuronal cells were subsetted from the full scRNA‐seq dataset for focused analysis. Clustering was performed on this neuronal population using a graph‐based clustering algorithm (Leiden, resolution = 0.7), resulting in the identification of 13 distinct neuronal clusters. Dimensionality reduction techniques, including PCA and Uniform Manifold Approximation and Projection (UMAP), were applied to visualize the clustering results.

To enhance biological interpretability, the 13 clusters were further consolidated into nine major neuronal subtypes (labeled as clusters a–i) based on the expression of uniquely enriched, statistically significant marker genes identified through DEG analysis.

The overall scRNA‐seq analysis workflow is outlined in Figure S1 (Supporting Information). After initial quality control (removal of low‐quality cells, normalization, and identification of highly variable genes), cell type annotation was performed using the scMRMA algorithm, referencing known marker genes to classify broad cell types such as neurons, astrocytes, and glial cells. Neurons were then isolated for in‐depth subtype analysis, followed by clustering and DEG‐based annotation into nine biologically meaningful neuronal categories.

### Comparative Analysis of Birtele et al. and La Manno et al.’s Papers

To gain exploratory insight into the dopaminergic features of the cortical organoid‐derived DA‐like cells, the single‐cell RNA sequencing data were compared with previously published datasets focusing on the human fetal ventral midbrain from Birtele et al.,^[^ ^15^ ^]^ and human midbrain data from La Manno et al.^[^ ^16^ ^]^ Although the cortical and midbrain regions were anatomically and developmentally distinct, this comparison was intended solely to assess whether the DA‐like cells exhibited partial transcriptional similarities to canonical midbrain dopaminergic neurons. Raw count data were obtained from GEO under accession numbers GSE192405 and GSE76381, respectively. Since the dataset from Birtele et al. did not include cell type annotations, the own annotation was performed as detailed below.

Regarding the cell type annotation for Birtele et al., the authors focused on data obtained from timepoints between 6 to 11 weeks. Data was normalized by applying log‐transformation (using the “LogNormalize” method in Seurat), and the top 2000 highly variable genes were identified. To visualize the data and identify clusters, principal component analysis (RunPCA) was conducted using the top highly variable genes. The top 20 precomputed principal components were utilized to calculate manifolds using UMAP methods (RunUMAP), and clusters were identified using the shared nearest neighbor algorithm (FindClusters). Subsequently, clusters were annotated by using cell type specific markers such as Radial glial cells (*FABP7 and SOX2*), Neuroblast (*NHLH2*), dopaminergic neurons (*TH, PBX1*), floor plate progenitors (*HMGB2, MKI67*), Serotonergic neurons (SYT1), glutamatergic neurons (*GRIA2, MEG3*), GABAergic neurons (*FOXD2, ONECUT2*), microglia (*CD74, TYROBP*), oculomotor/trochlear nucleus neurons (*OTN*,*NEFL, NEFM*), pericytes/endothelial cells (*RGS5, CLDN5*), and RBCs (*HMBS, SOD1*).

Subsequently, the organoid data were integrated with the 2 published datasets. The top 2000 highly variable genes were identified and utilized to calculate PCA embeddings. To account for batch effect and remove technical variation, the Harmony algorithm^[^ ^39^ ^]^ was applied to perform batch correction which generated the batch corrected PCA embeddings. These were used to compute manifolds for visualizations.

To characterize the correlation patterns, the analysis was stratified by cell type. For dopaminergic neurons, cells annotated as DA, and hDA were included as they were annotated in each dataset. For GABAergic, cells annotated as GABAergic, hGaba, and hNbGaba were included. For neuronal progenitor/neuroblast cells annotated as hNProg, hNbM, hNbML, neural progenitors, and neuroblasts were included. Correlation analysis was conducted between time points of each neuron subtype across different datasets. To account for variation due to the different number of cells in each group, the mean count of the log‐normalized expression values for each cell type within each dataset was calculated. Subsequently, Pearson correlation between the groups was estimated using the mean expression values applying the Euclidean distance and ward's linkage method. Seurat (v4.3.0.1), Scanpy (v1.9.1),^[^ ^40^ ^]^ R (v4.2.2), and Python (v3.9.9) were used for performing the bioinformatics analysis.

### K‐Means Clustering Guided scRNA‐Seq Cell Type Annotation

ScRNA‐seq data in H5AD format were processed using the Scanpy package.^[^ ^40^ ^]^ Quality control filters were applied to retain cells with 200–5000 detected genes and less than 20% mitochondrial gene expression. Genes were filtered using sc.pp.filter_genes(adata, min_cells = 3) and sc.pp.filter_genes(adata, min_counts = 20). Gene counts were normalized to 10 000 per cell and log‐transformed to stabilize variance. Highly variable genes were selected based on mean expression (0.1–8) and minimum dispersion of 0.5. PCA was performed using the ARPACK solver, and principal components were standardized with StandardScaler to ensure equal contribution during clustering.

A two‐stage *K*‐means clustering approach was implemented using scikit‐learn.^[^ ^41^ ^]^ In the first stage, broad cell populations were identified by testing *K*‐values from 2 to 30, with silhouette scores used to determine the optimal cluster number. Multiple random initializations were performed to ensure robustness. Cell type annotation was conducted by matching known marker genes to each cluster^[^ ^42^ ^]^ with visualization through dot plots. Non‐neuronal cell types (astrocytes, radial glial cells, melanocytes, ependymal cells, tanycytes, and fibroblasts) were identified and excluded from subsequent analysis. In the second clustering stage, remaining cells presumed to represent neuronal subtypes underwent focused K‐means clustering to identify GABAergic, dopaminergic, and glutamatergic neurons. Neurotransmitter‐specific markers were used to validate subtype identities through dot plot visualization.

Differential gene expression analysis was performed between dopaminergic, GABAergic,^[^ ^1^ ^]^ and glutamatergic cell types, as well as between experimental groups for each cell type using the Wilcoxon rank‐sum test (scanpy.tl.rank_genes_groups) with Bonferroni correction for multiple testing. DEGs were identified based on adjusted *p*‐value ≤ 0.05.

Volcano plots were generated to visualize differential gene expression patterns using R with ggplot2.^[^ ^43^ ^]^ For each comparison, the top 10 upregulated and top 10 downregulated genes were annotated based on a composite score of absolute log fold change and statistical significance. Significance thresholds were indicated by dashed lines at log fold change = ±1 and adjusted *p*‐value = 0.05.

Gene Ontology enrichment analysis was conducted using the clusterProfiler package in R^[^ ^44^ ^]^ based on significantly DEGs meeting the established criteria (adjusted *p*‐value ≤ 0.05 and absolute fold change ≥ 1).

### Pseudotime Analysis using scVelo

Neuron pseudotime trajectory analysis was performed using scVelo (https://github.com/theislab/scvelo) to investigate cellular differentiation dynamics across different experimental conditions. The analysis incorporated both spliced and unspliced mRNA expression data to infer RNA velocity and reconstruct developmental trajectories.

Latent time was computed from dynamic models to determine trajectory timing alongside cellular lineages. The latent time metric ranges from 0 to 1 and represented each cell's internal developmental state based on transcriptional dynamics.

Trajectory visualization and connectivity analysis were implemented using PAGA (Partition‐based Graph Abstraction).^[^ ^45^ ^]^ In the PAGA representation, nodes correspond to cell clustered with edge width indicating connectivity strength between partitions. PAGA connectivity was calculated as the ratio of observed inter‐cluster edges to expected edges under random assignment. Edge directions represented pseudotime progression from earlier to later developmental stages.

Individual cell pseudotime was initially calculated and subsequently corrected using latent time estimations. Steady‐state equilibrium modeling was performed using linear regression in pseudotime with the assumption of common splicing rates across cell types. Splicing kinetics were integrated using a likelihood‐based dynamical model to capture transcriptional regulatory dynamics.

### Metabolomic Analysis

Comprehensive metabolomic profiling was performed using UPLC‐MS/MS analysis at BGI Hong Kong Limited. Cortical organoid samples were homogenized in methanol: acetonitrile: water (2:2:1) containing internal standards (d3‐Leucine, 13C9‐Phenylalanine, d5‐Tryptophan, 13C3‐Progesterone) using a cryogenic grinder, followed by vortex mixing and centrifugation at 12 000 × g at 4 °C. The supernatants were concentrated using a refrigerated vacuum concentrator prior to analysis. Chromatographic separation was achieved on a HSS T3 column (2.1 × 100 mm, 1.8 µm) with 0.1% formic acid in water and acetonitrile as mobile phases. Mass spectrometry was performed in both positive and negative ESI modes (m/z 70‐1050) using data‐dependent acquisition. Raw data were processed with Compound Discoverer 3.3 and annotated against the BGI Metabolome Database, mzCloud, and ChemSpider. Data preprocessing included Probabilistic Quotient Normalization, QC‐based LOESS signal correction for batch effects, and removal of metabolites with >30% CV in QC samples. Quality control was maintained throughout using pooled QC samples to monitor analytical variability.

### Statistical Analysis

The data were presented as mean ± standard deviation for a sample size of at least three. All statistical analyses were conducted using GraphPad Prism 8.0.2. For non‐normally distributed datasets, the Mann–Whitney *U*‐test was applied. Normality was assessed using the Shapiro–Wilk test, and outliers were identified using the ROUT method. All data were presented as mean ± standard deviation, and a *p‐*value ≤ 0.05 was considered statistically significant. The sample sizes and corresponding *p‐*values were listed in Table S32 (Supporting Information).

### Ethics Approval

The project was approved by the Western Norway Committee for Ethics in Health Research (REK nr. 2012/919). Tissues were acquired with written informed consent from all patients, and the experiments conformed to the principles set out in the WMA Declaration of Helsinki and the Department of Health and Human Services Belmont Report.

## Conflict of Interest

K.X.L. and N.L. are shareholders of NorVita Med AS, which holds no IP or rights to the model or methods described and received no payment or incentive for this work. Shareholders have never received payment or dividends. All other authors declare no competing interests.

## Author Contributions

K.L. contributed to the conceptualization. K.X.L. and Z.Z. contribute to the methodology. K.X.L., Z.Z., T.Y., N.L., S.D., L.X. contribute to the investigation. K.X.L., Z.Z., and T.Y. contributed to writing the original draft. All authors contribute to writing the review and editing. K.X.L. and G.Y. contribute to the funding acquisition and to the resources. K.X.L. contributes to the supervision. All authors agree to the authorship.

## Supporting information



Supporting Information

## Data Availability

The datasets generated and analyzed during the study are included with the Supporting Information. The RNA sequencing analysis read count data can be accessed in the NCBI Gene Expression Omnibus (GEO) data deposit system with an accession number GEO GSE234659. All other data is available from the corresponding author upon request.
