# Supplementary material for: Metformin Restores Mitochondrial Function and Neurogenesis in POLG Patient‐Derived Brain Organoids
Source: Adv Sci (Weinh). 2025 Dec 8;13(4):e17721. doi: 10.1002/advs.202417721 (PMC12822396; doi:10.1002/advs.202417721)
Supplement: Supplementary file 1 — Supporting Information [file ADVS-13-e17721-s001.pdf]

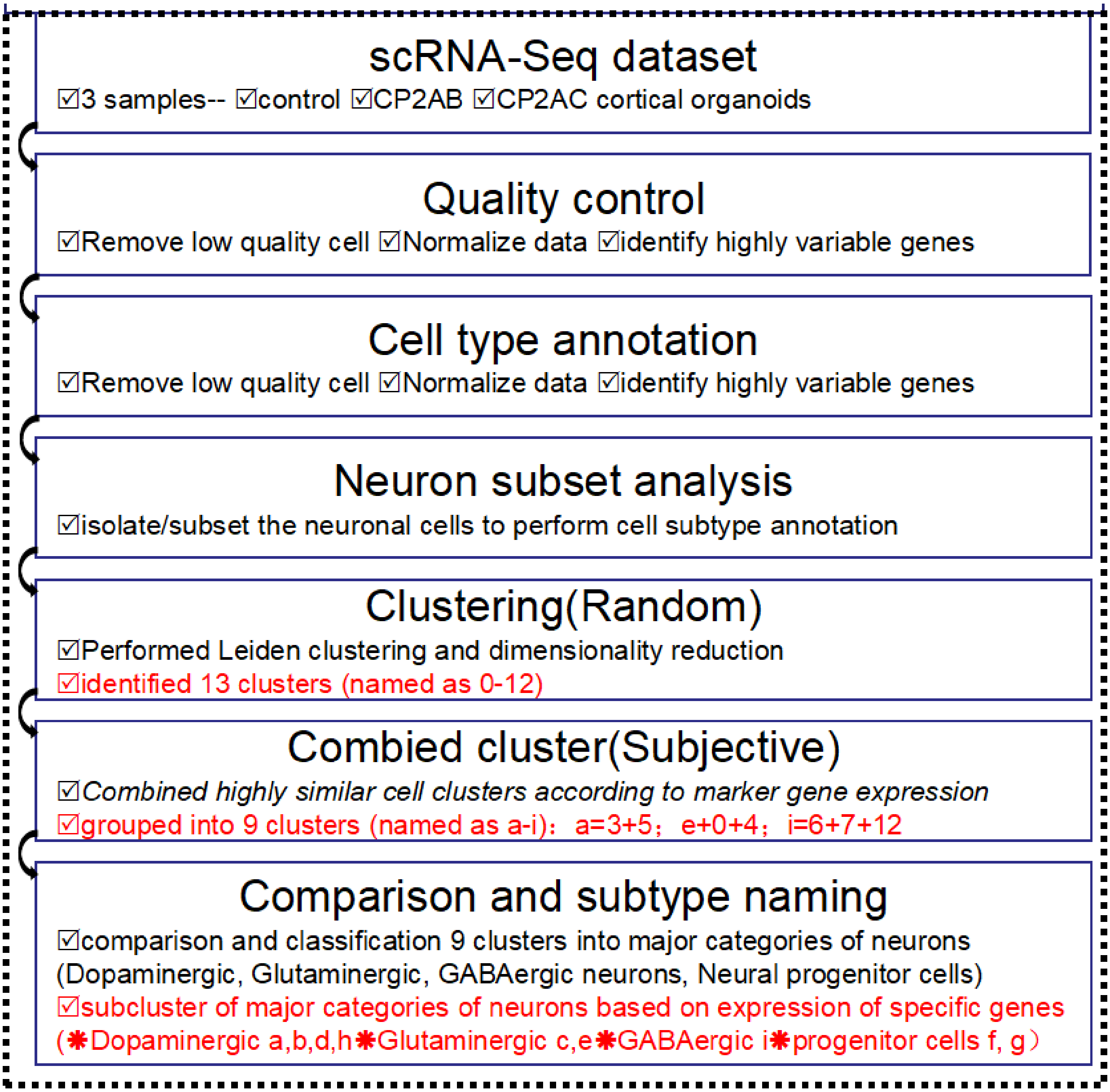

Figure S1. Workflow of scRNA-seq analysis of cortical organoids. The dataset includes three sample groups: control, POLG patient, and POLG patient treated with metformin. After initial quality control steps—including filtering low-quality cells and identifying highly variable genes—cell type annotation was performed. Neuronal cells were isolated for subset analysis and further annotated. Clustering was carried out using Leiden algorithm and dimensionality reduction, resulting in 13 initial neuronal clusters (labeled 0–12). Based on similarity in gene expression, these clusters were manually combined into 9 refined groups (a–i), such as a = 3+5, e = 0+4, and i = 6+7+12. These 9 clusters were then mapped to broader neuronal subtypes, including dopaminergic neurons (clusters a, b, d, h), glutamatergic neurons (clusters e, f, g), GABAergic neurons (clusters b, c, d), and neural progenitor cells (clusters f, g).

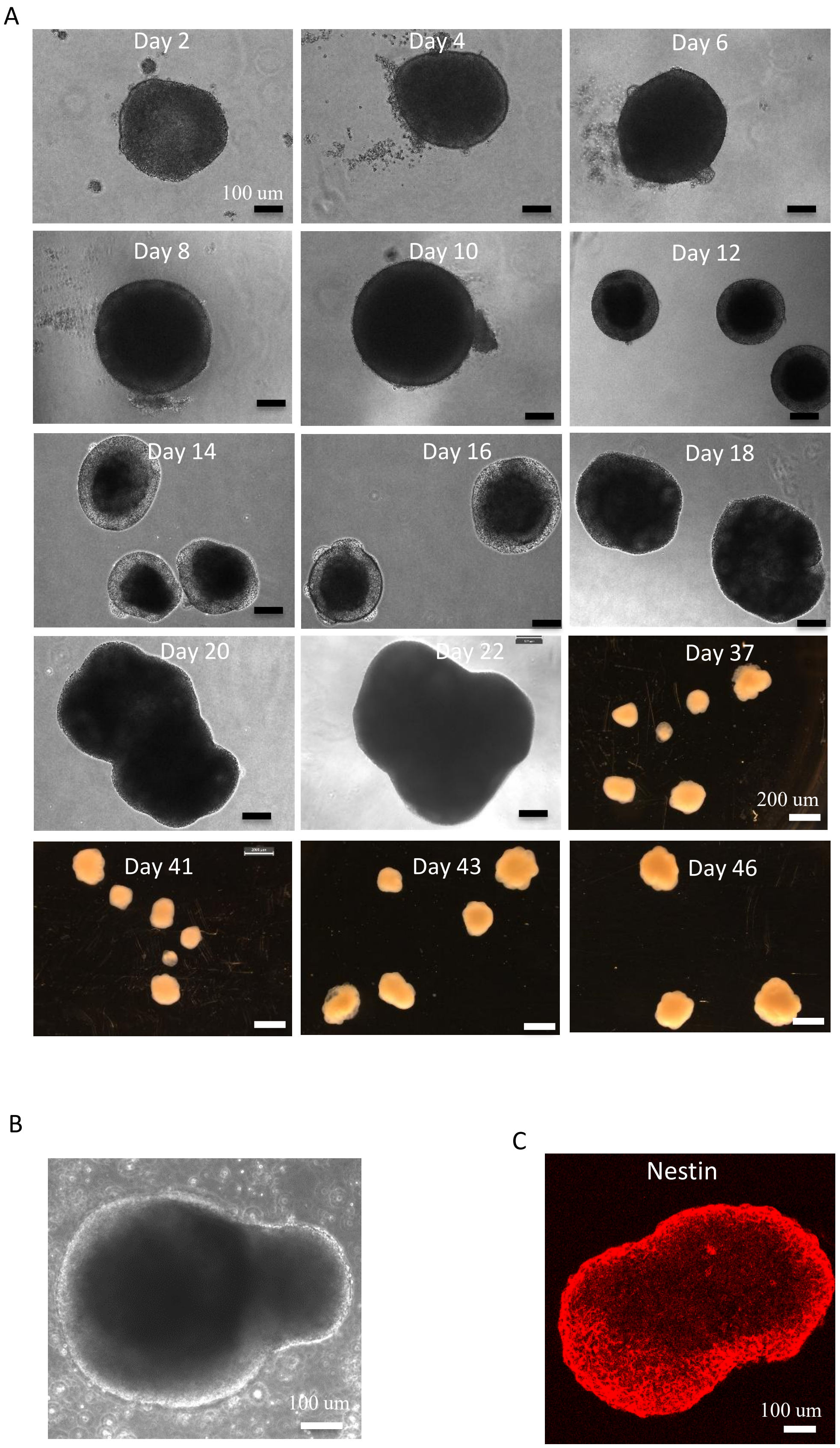

Figure S2. Morphological and molecular characterization of cortical organoid differentiation from day 2 to day 46. (A) Phase-contrast time course showing progressive developmental stages of neural differentiation. (B) High-magnification views of representative neural sphere morphology at key time points. (C) Immunofluorescence validation of Nestin expression (red) in neural progenitors, with DAPI nuclear counterstain (blue). Scale bars: 100 μm or 200 μm.

A

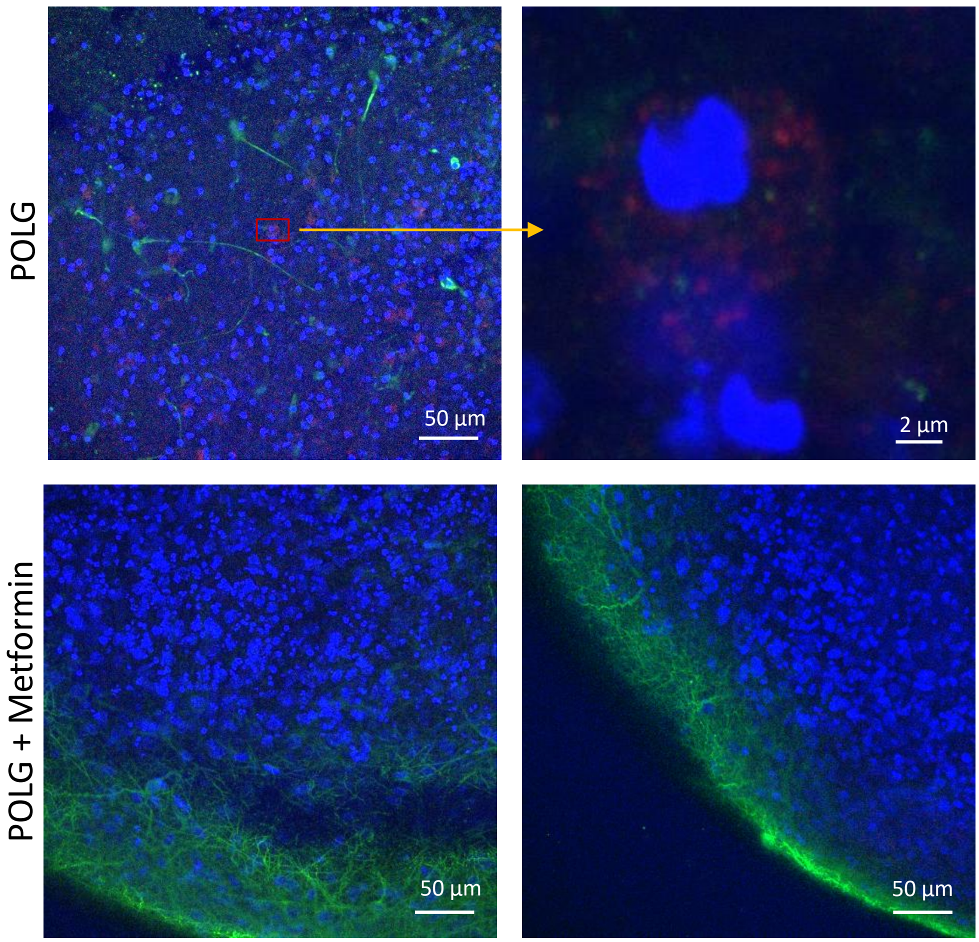

B

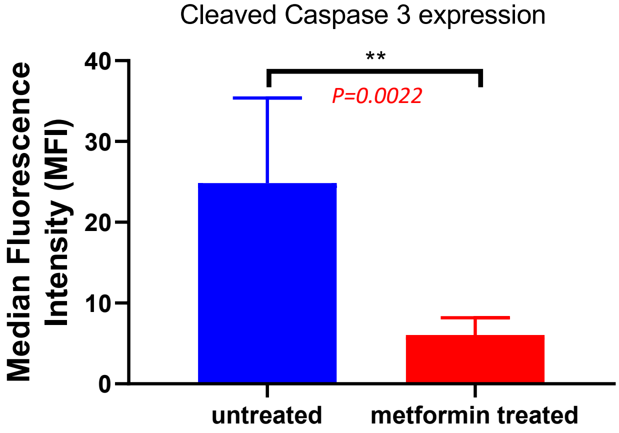

Figure S3. Immunofluorescence validation (A) and quantification (B) of the cleaved caspase 3 (red) and MAP2 (green) in *POLG* cortical organoid before and after metformin treatment, with DAPI nuclear counterstain (blue). Scale bars: 2 µm or 50 µm.

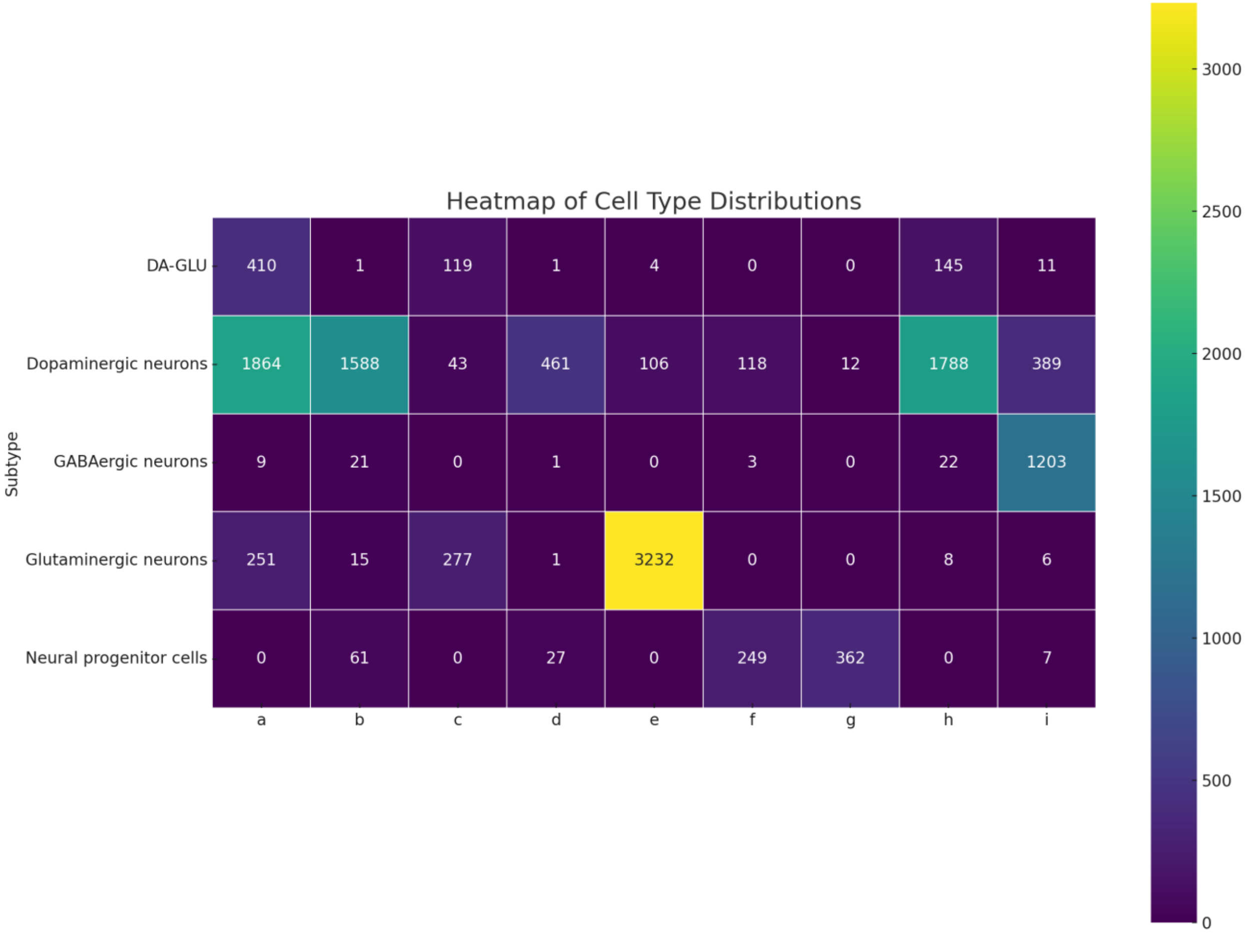

Figure S4. The heatmap illustrateing the distribution of cell types within each subcluster of cortical organoids derived from patient iPSCs.

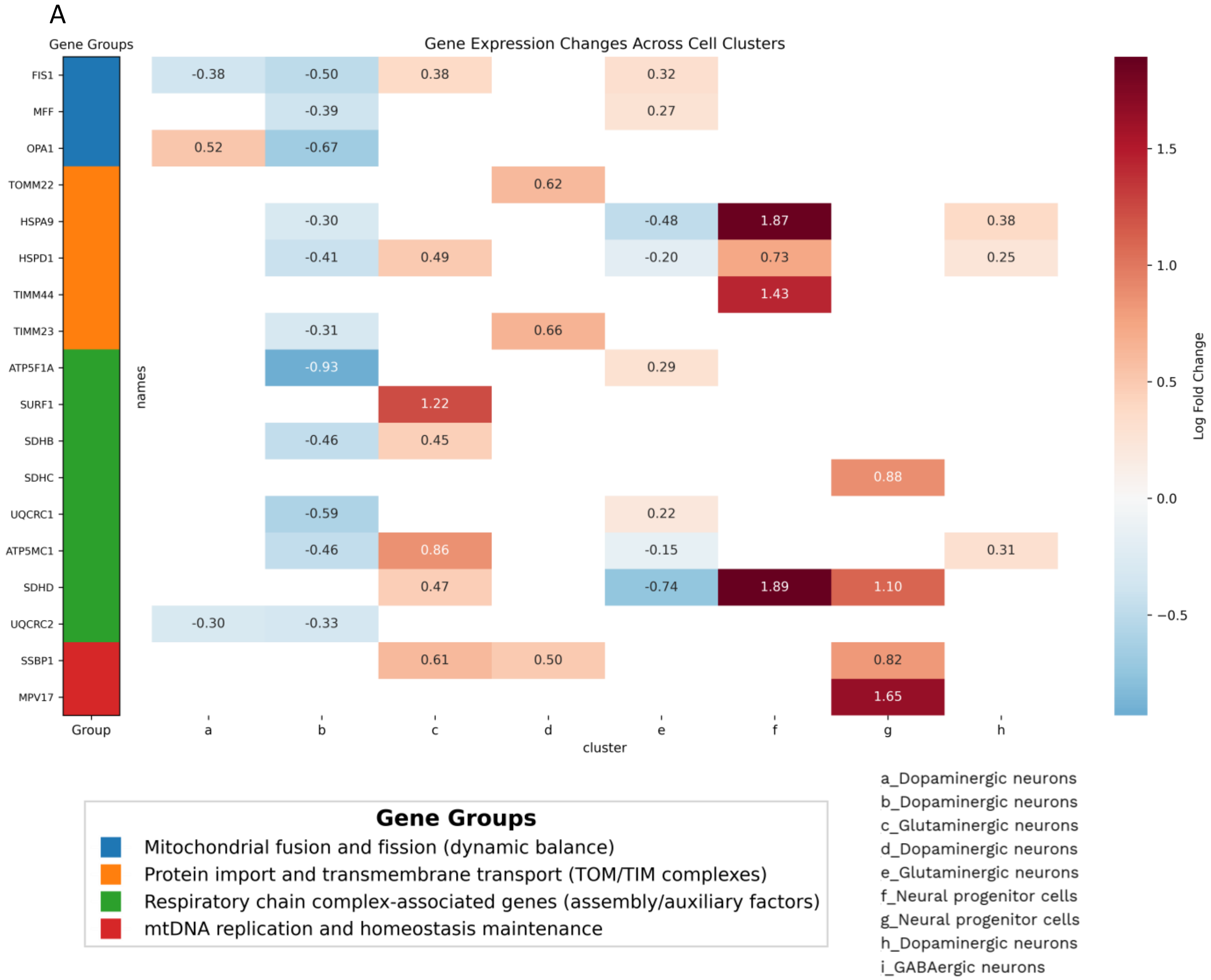

Figure S5. Differential gene expression patterns across cell subgroups. Heatmap displaying **log fold changes of nuclear-encoded mitochondrial function-related genes** across nine cell clusters (a-i). Each cell represents the log2 fold change of gene expression relative to the reference condition, with red indicating upregulation and blue indicating downregulation. Only genes present in the predefined gene set were included in the analysis. Numbers in each cell show the exact log fold change values. Cell clusters showed varying numbers of differentially expressed genes (ranging from 0 to 11 genes per cluster), reflecting distinct transcriptional profiles across neuronal subpopulations. Missing values (white cells) indicate genes that were not significantly differentially expressed in those particular clusters.

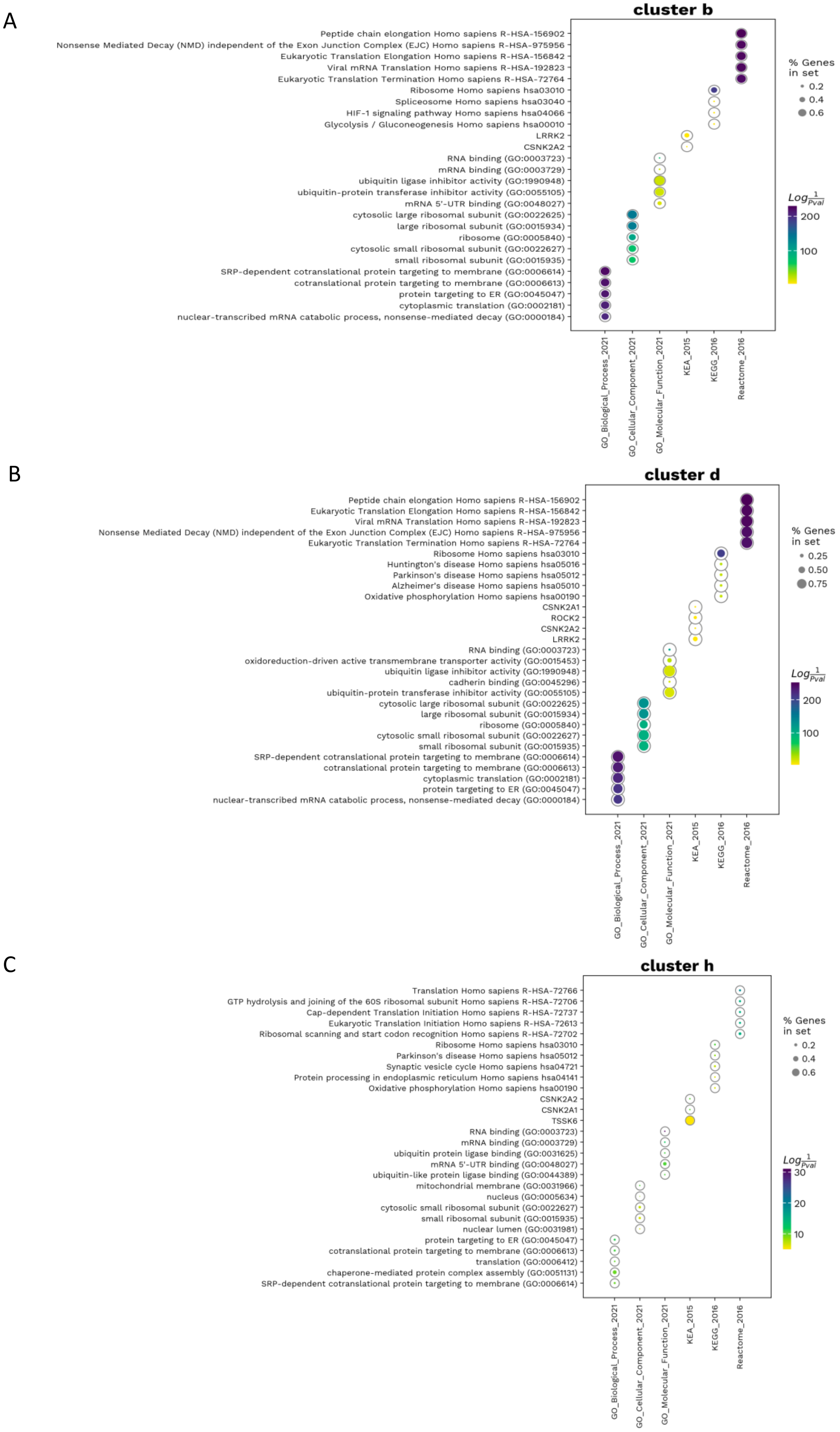

Figure S6. The enrichment analysis of GO, KEGG pathways, and Reactome pathways for DEGs in neurons from clusters b (A), d (B), and h (C).

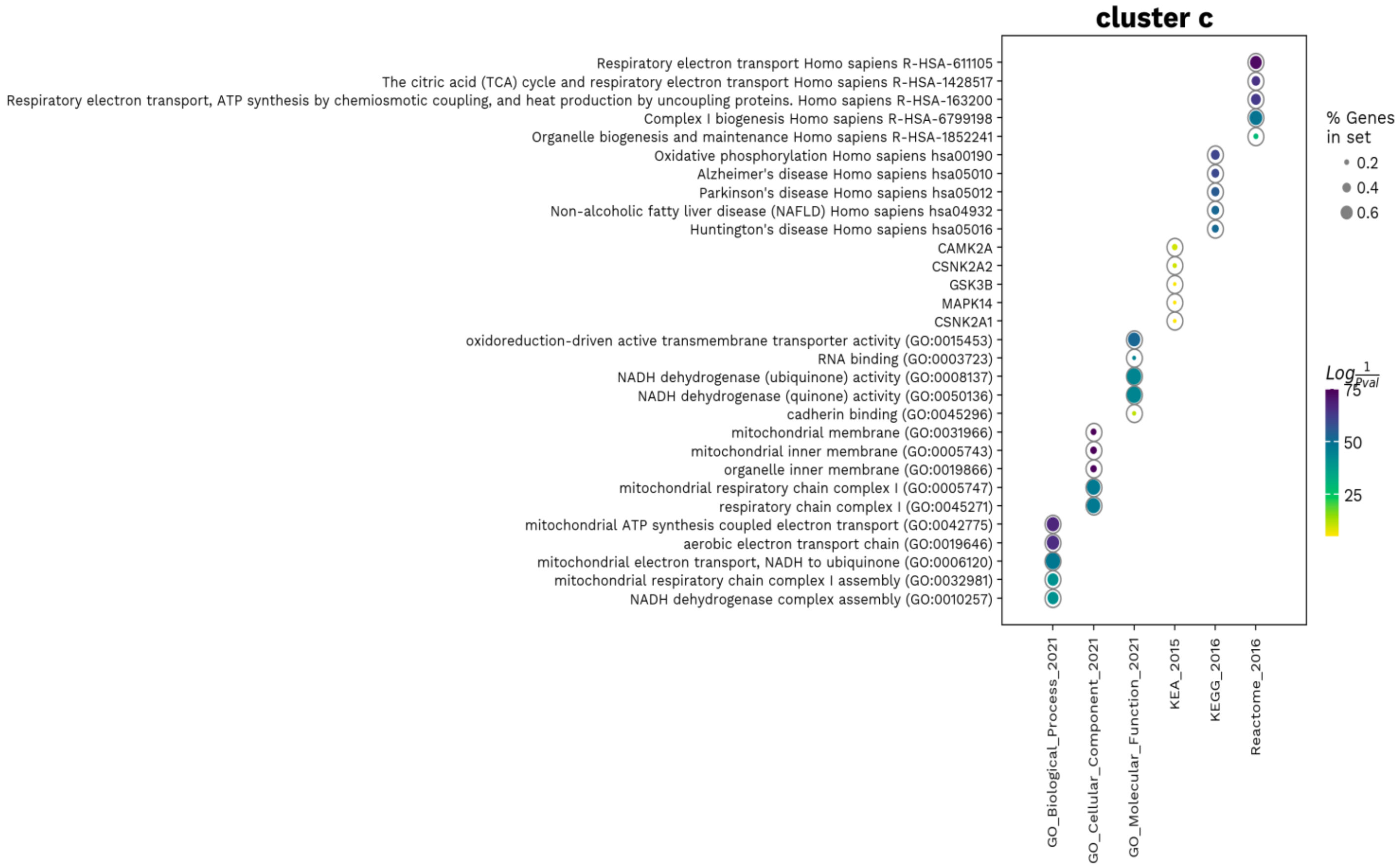

Figure S7. The enrichment analysis of GO, KEGG pathways, and Reactome pathways for DEGs in neurons from clusters c.

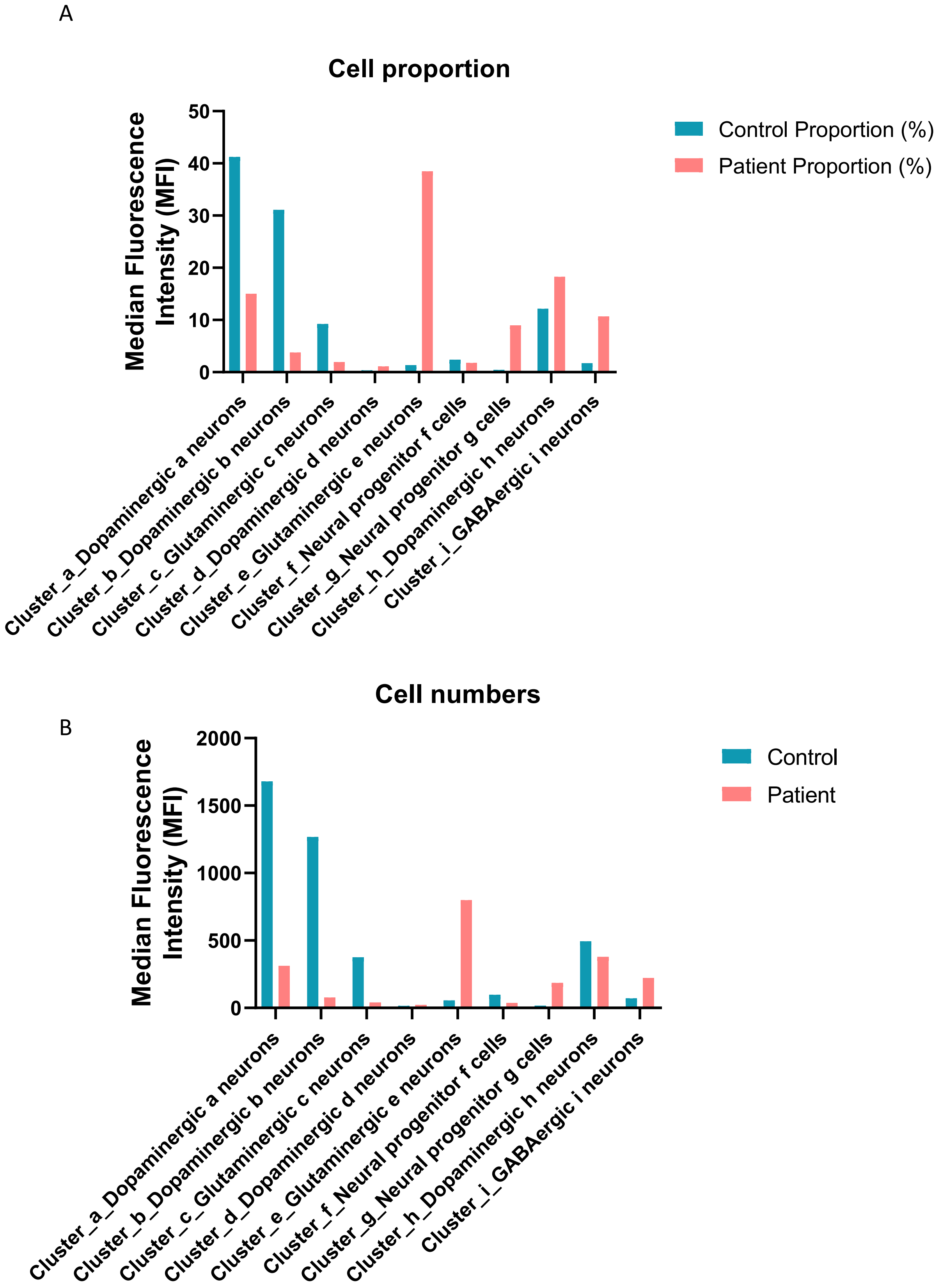

Figure S8. Cell proportions and counts of neuronal clusters in cortical organoids derived from patient iPSCs compared to controls. (A). Proportion of cells in neuronal clusters within CP2A cortical organoids derived from patient iPSCs, compared to control organoids. (B). Total number of cells in each neuronal cluster, highlighting differences between CP2A cortical organoids and controls.

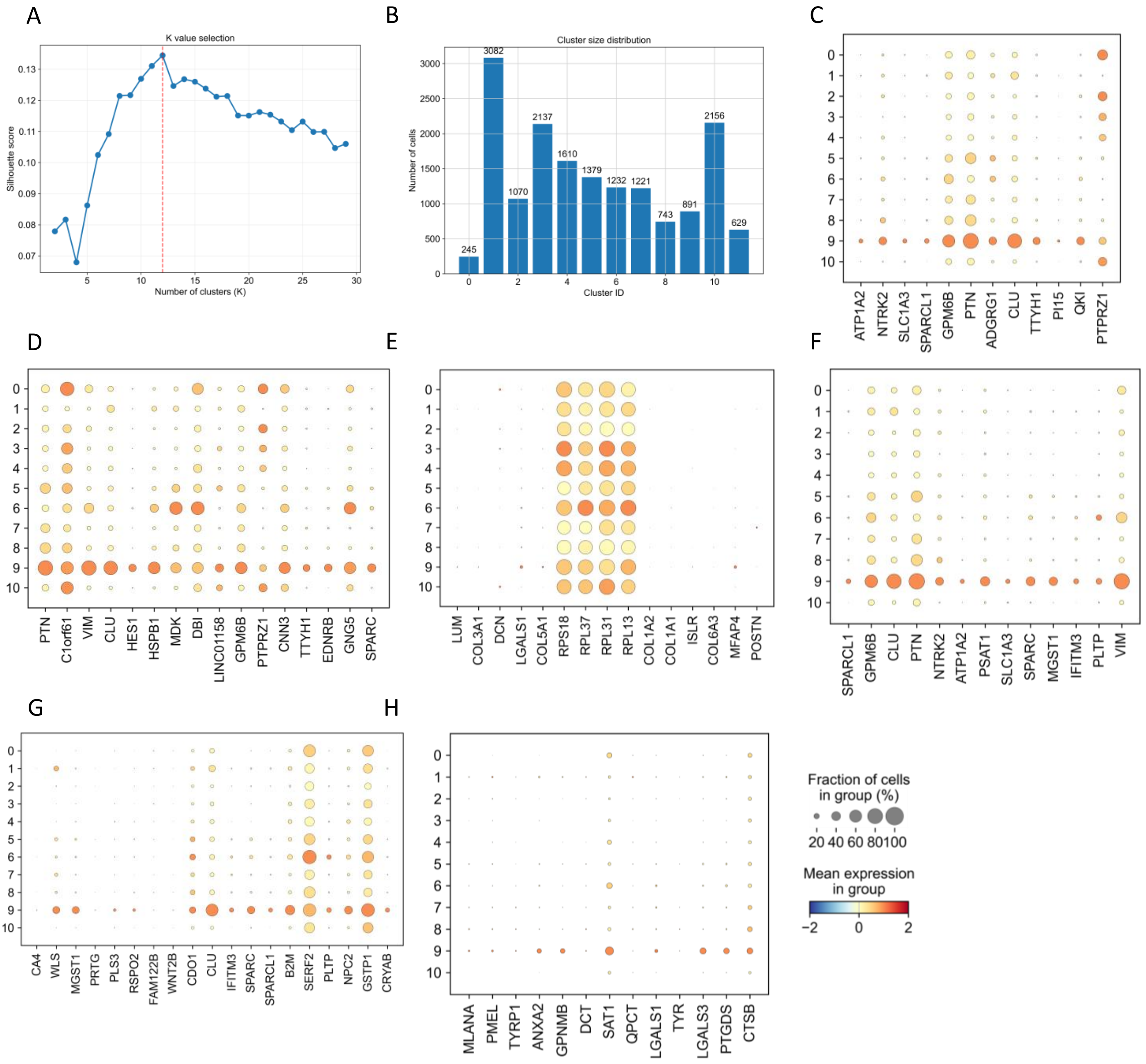

Figure S9. k-means clustering identifies non-neuronal cell populations in single-cell transcriptomic data. (A) Silhouette score analysis used to determine the optimal number of clusters. The plot shows silhouette scores (y-axis) across k-values ranging from 2 to 30 (x-axis), with the highest score observed at k = 11. (B) Bar plot showing the distribution of cell numbers across the 11 identified clusters. (C–H) Dot plots illustrating expression of cell type–specific marker genes for non-neuronal populations: (C) astrocytes, (D) radial glial cells, (E) fibroblasts, (F) tanycytes, (G) ependymal cells, and (H) melanocytes. Dot size indicates the proportion of cells within each cluster expressing the given gene, while color intensity represents z-scaled expression levels (blue-white-red gradient).

.

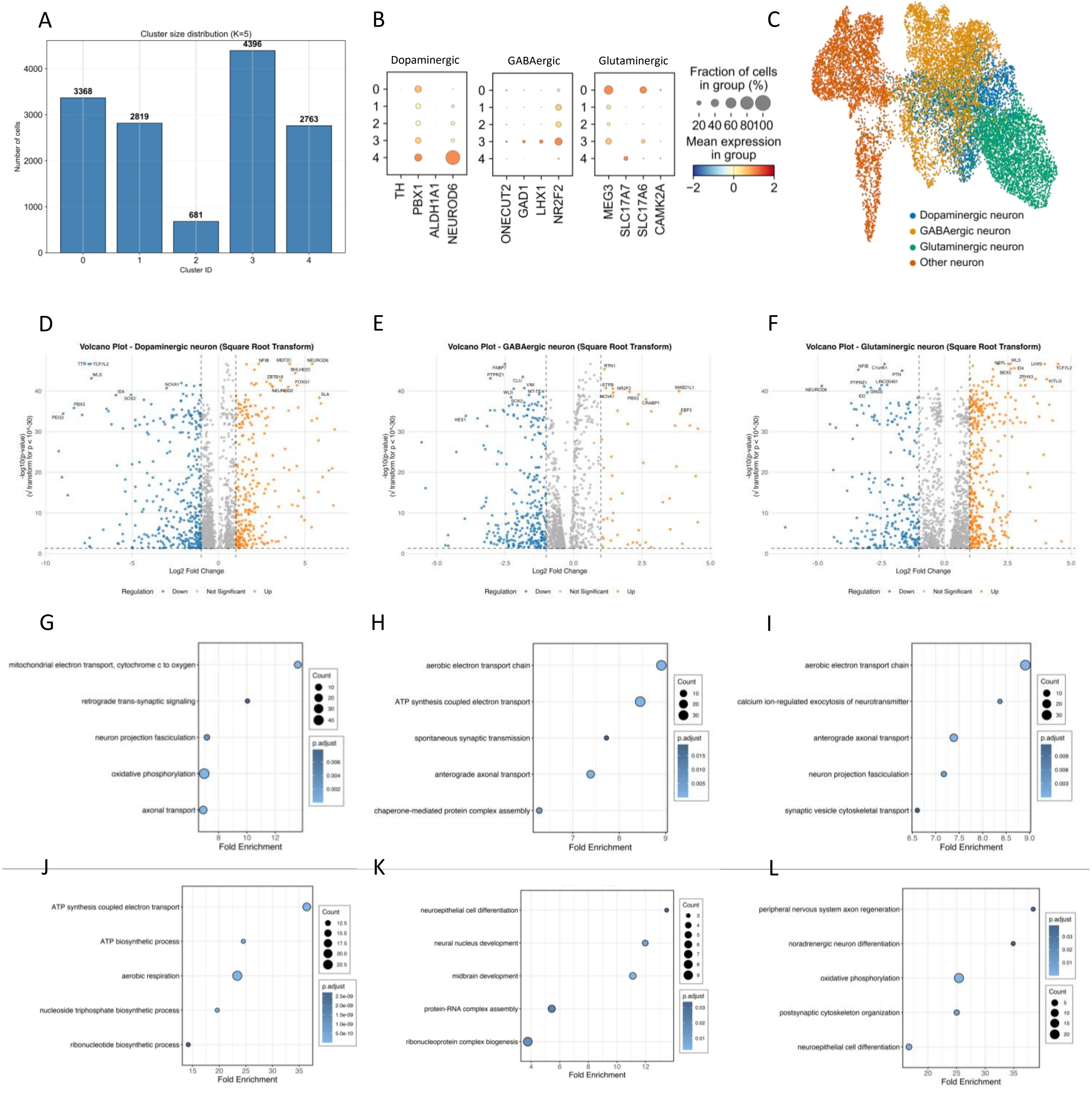

Figure S10. Second-round k-means clustering and transcriptomic analysis of neuronal subtypes. (A) Bar plot showing the distribution of cell numbers across identified neuronal clusters. (B) Dot plots displaying expression of neuron subtype–specific markers: dopaminergic neurons (cluster 4), GABAergic neurons (cluster 3), glutamatergic neurons (cluster 0), and other neuronal populations (clusters 1 and 2). Dot size indicates the percentage of cells expressing each gene within a given cluster, while color intensity reflects z-scaled expression levels (blue-white-red gradient). (C) UMAP visualization of neuronal clusters based on second-round k-means clustering. (D–F) Volcano plots highlighting DEGs in (D) dopaminergic, (E) GABAergic, and (F) glutamatergic neurons. Orange and blue dots represent significantly upregulated and downregulated genes, respectively (log2FC ≥ ±1). (G–I) Bubble plots showing significantly enriched pathways for (G) dopaminergic, (H) GABAergic, and (I) glutamatergic neurons, based on DEG analysis. (J–L) Bubble plots of pathway enrichment based on specific DEG comparisons: (J) downregulated genes in GABAergic neurons (POLG vs. control), (K) upregulated genes in GABAergic neurons (POLG + metformin vs. POLG), and (L) upregulated genes in glutamatergic neurons (POLG + metformin vs. POLG).

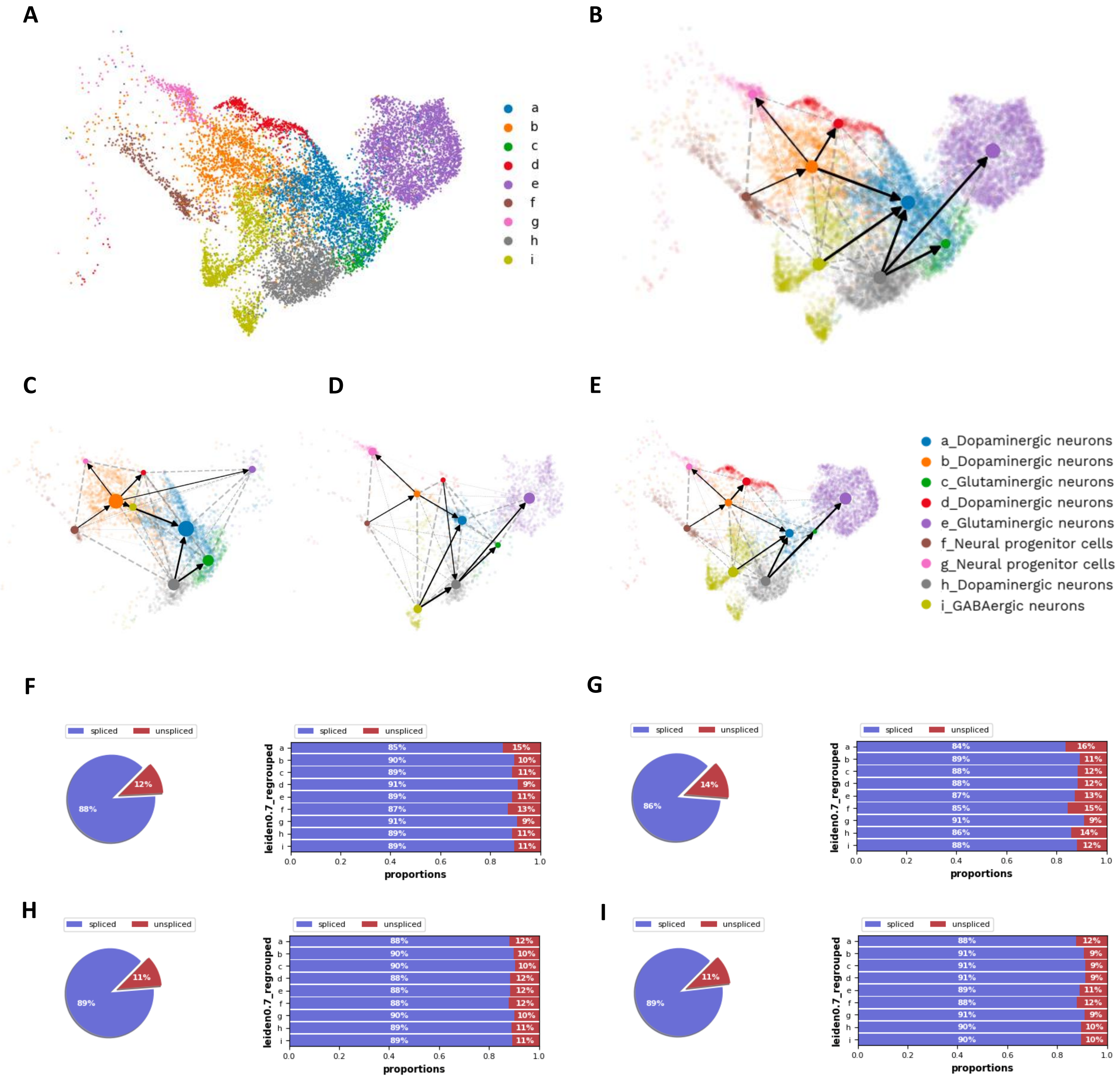

Figure S11. Pseudotime trajectory analysis reveals condition-specific neuronal differentiation dynamics. (A) UMAP visualization of neural organoid cells, colored by nine distinct neuronal subtypes. (B) Partition-based graph abstraction (PAGA) velocity graph displaying the directed connectivity between neuronal clusters. Node size represents cluster abundance, edge width indicates connection strength, and arrows reflect pseudotime progression from early to late developmental stages. (C–E) Condition-specific PAGA velocity graphs for (C) control, (D) POLG, and (E) POLG + Metformin samples, highlighting divergent trajectory patterns and altered inter-cluster connectivity across conditions. (F–I) RNA velocity analysis showing the proportion of spliced and unspliced transcripts across (F) all samples combined and separately by condition: (G) control, (H) POLG, and (I) POLG + Metformin. These distributions reflect transcriptional activity and dynamic gene expression during neuronal differentiation.

A

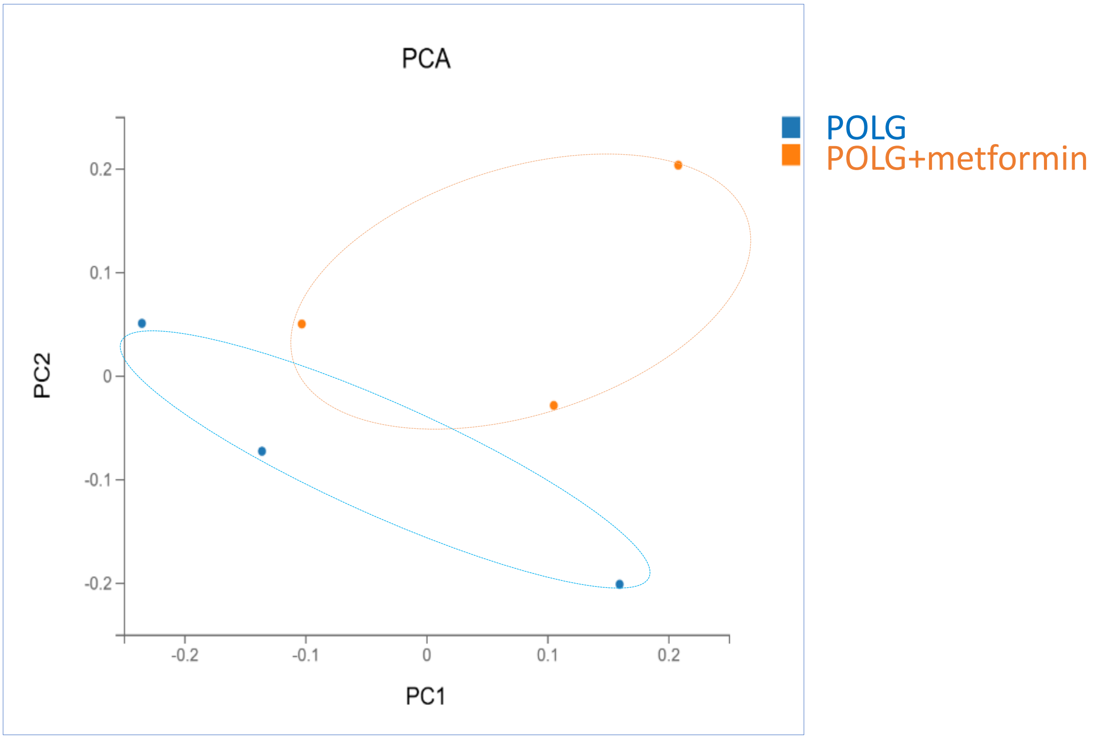

B

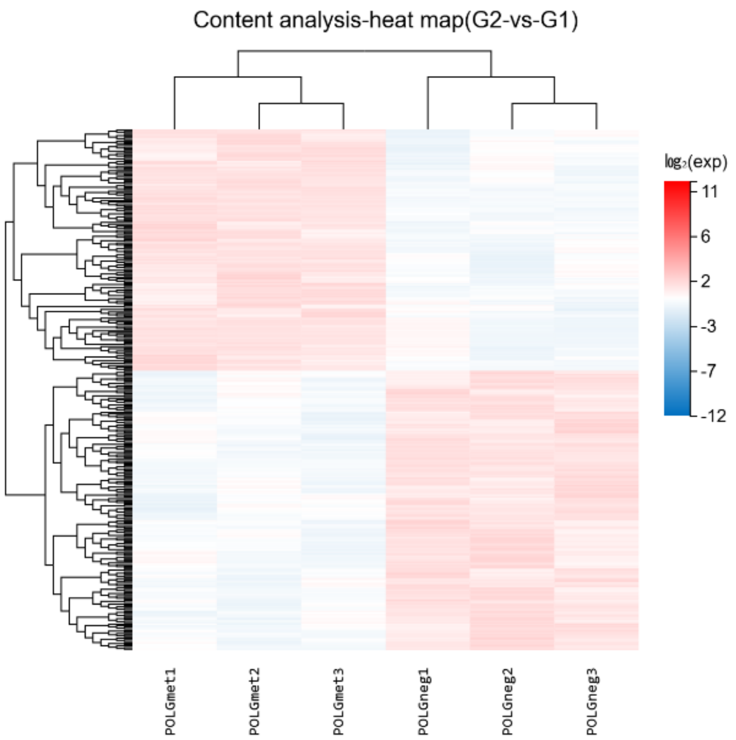

Figure S12. Metabolomic analysis of POLG cortical organoids with and without metformin treatment. (A) Principal component analysis (PCA) of global metabolomic profiles in POLG (blue) and metformin-treated POLG (orange) cortical organoids, showing distinct clustering of the two groups. (B) Heatmap of differentially expressed metabolites comparing POLG + metformin (G2) versus untreated POLG (G1). Rows represent individual metabolites, and columns represent biological replicates. Color scale represents log_2-transformed expression intensity. Metformin treatment induced broad metabolic reprogramming compared to POLG controls.

A

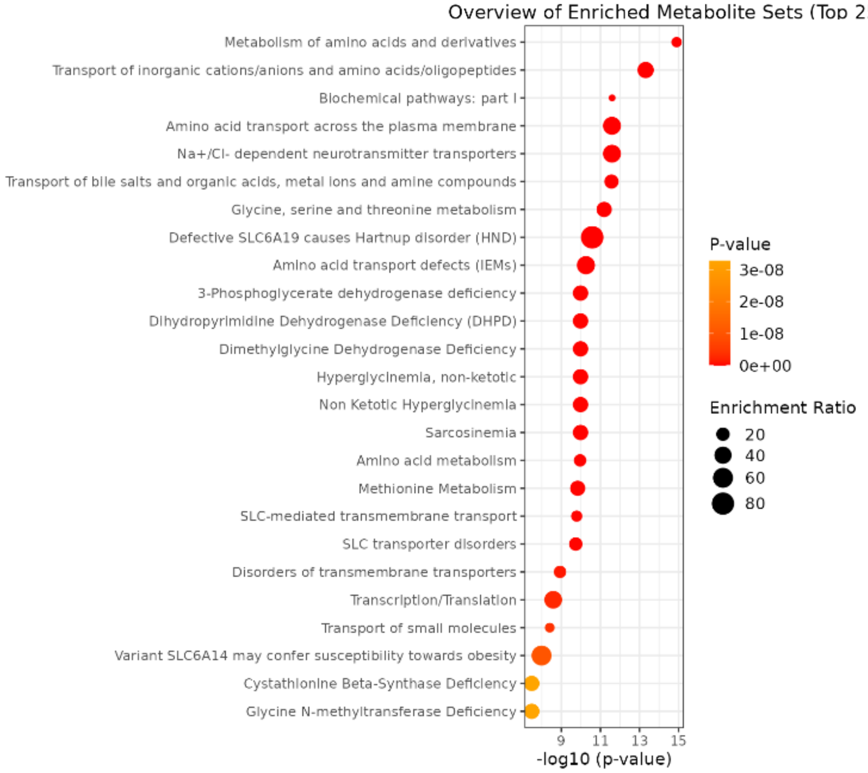

B

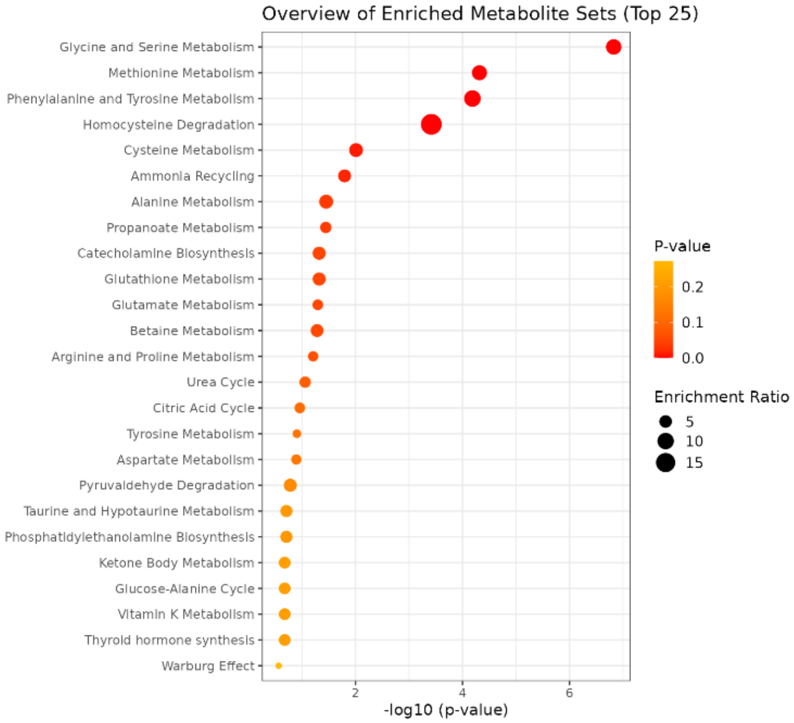

Figure S13. Metabolite set enrichment analysis in metformin-treated POLG cortical organoids. (A) Overview of significantly enriched metabolite sets between POLG and metformin-treated POLG samples. Each dot represents a metabolic pathway, colored by –$\log_{10}$(p-value) and sized by enrichment ratio. Amino acid metabolism and membrane transporter pathways were most significantly affected. (B) Top 25 enriched pathways ranked by statistical significance. Notable pathways include glycine and serine metabolism, methionine metabolism, homocysteine degradation, catecholamine biosynthesis, glutamate metabolism, and mitochondrial-related pathways (e.g., TCA cycle, ketone body metabolism). Color represents p-value, and dot size represents enrichment ratio..

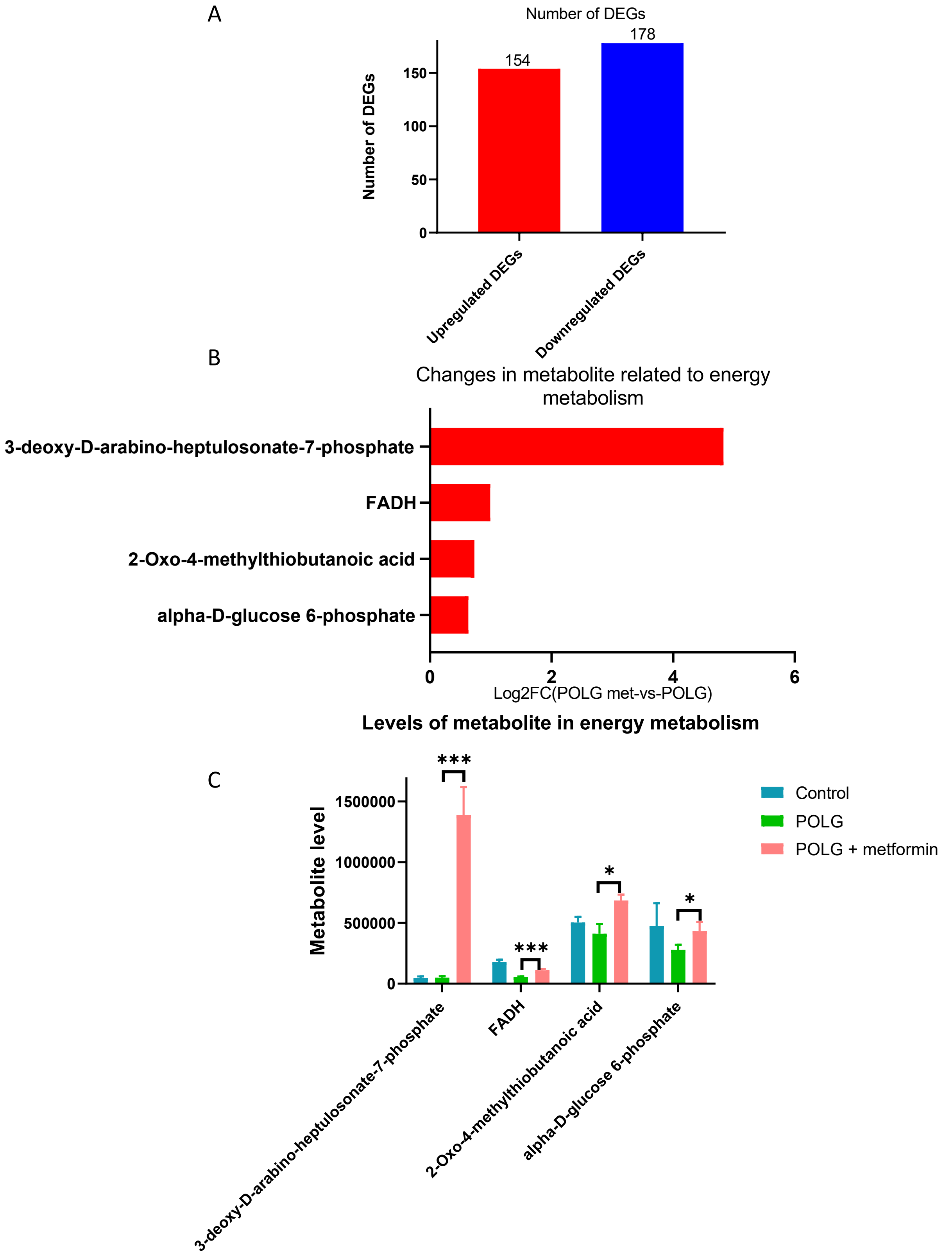

Figure S14. Metformin treatment enhances energy metabolism in POLG patient-derived brain organoids. (A). Bar graph showing the number of significantly altered metabolites in metformin-treated POLG brain organoids compared to untreated controls. (B) Pathway enrichment analysis of significantly upregulatedenergy metabolism related pathways in metformin-treated POLG brain organoids compared to untreated POLG samples. (C) Quantification of selected upregulated metabolites in metformin-treated POLG organoids compared to untreated POLG samples. Data are expressed as mean ± SD from three independent replicates. Statistical significance was determined by one-way ANOVA followed by Tukey's post hoc test; *$p < 0.05$..

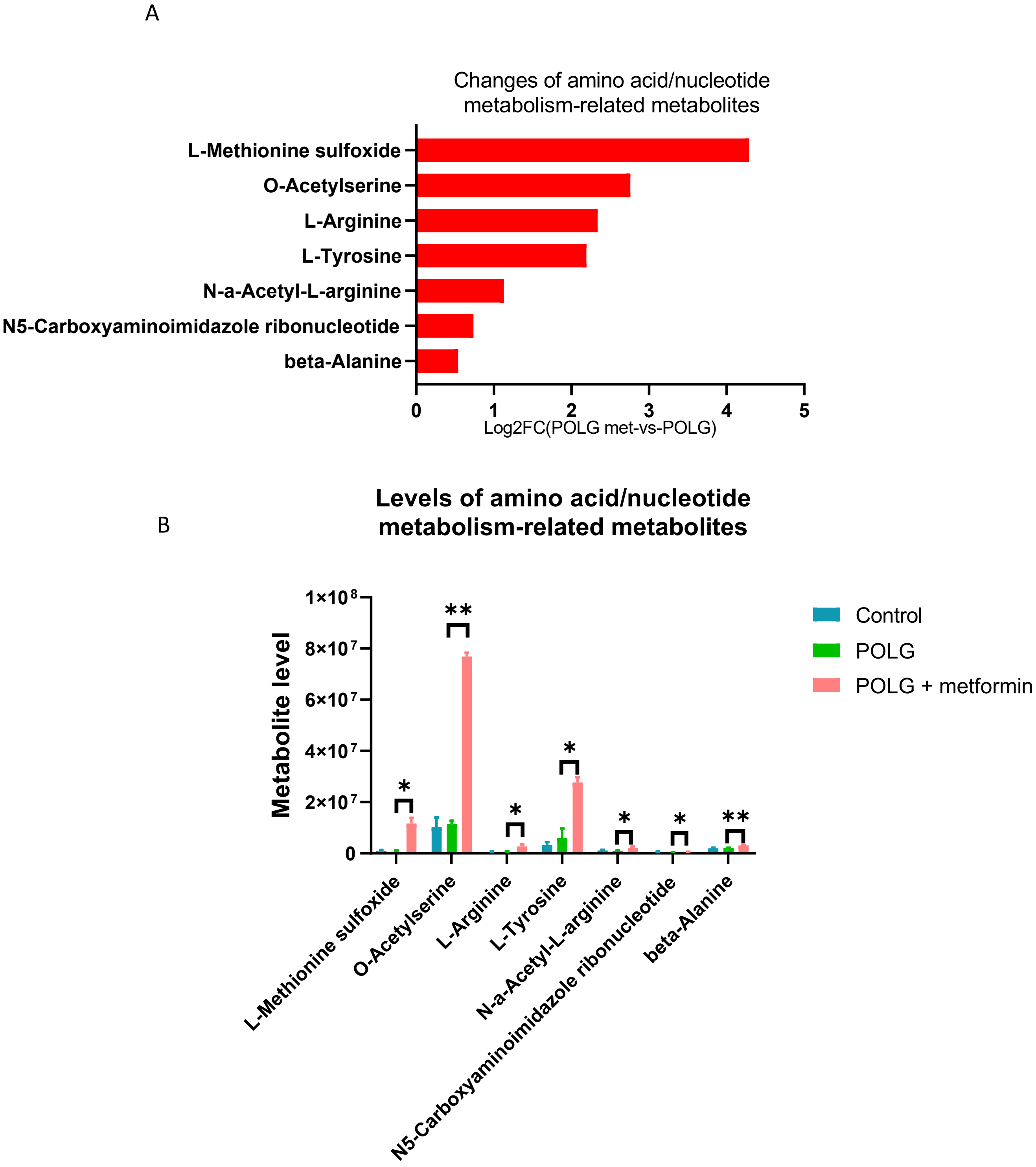

Figure S15. Metformin treatment upregulates amino acid and nucleotide metabolism pathways in POLG brain organoids. (A) Pathway enrichment analysis of significantly upregulated pathways related to amino acid and nucleotide metabolism. (B) Quantification of significantly upregulated amino acid and nucleotide-related metabolites in metformin-treated POLG brain organoids. Data represent mean ± SD from three independent biological replicates. Statistical significance was determined using Mann–Whitney U test; *$p < 0.05$.

A

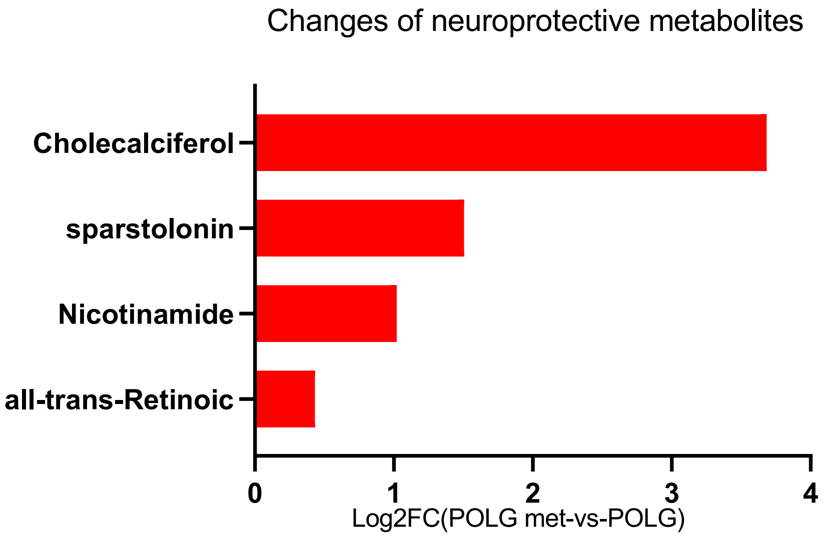

B

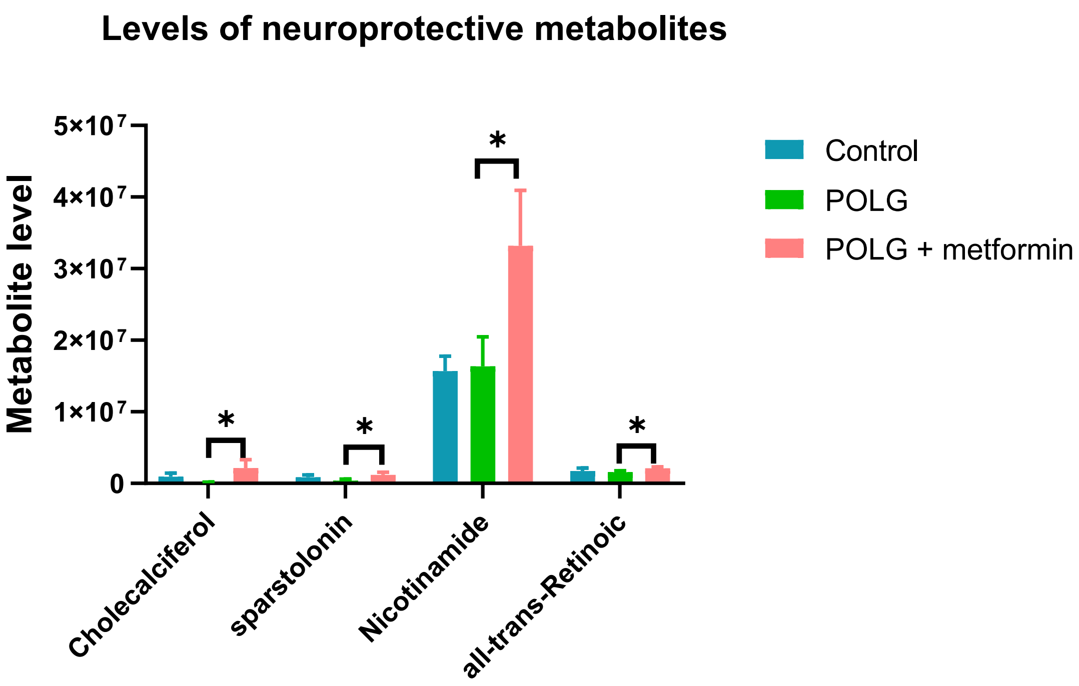

Figure S16. Upregulation of neuroprotective metabolites in metformin-treated POLG corticalorganoids. (A) Pathway enrichment analysis of upregulated neuroprotective metabosium related pathways. (B) Quantification of selected upregulated europrotective metabolites in metformin-treated POLG organoids compared to untreated POLG samples. Data are expressed as mean ± SD from three independent replicates. Statistical significance was determined by one-way ANOVA followed by Tukey's post hoc test; *p < 0.05.

A

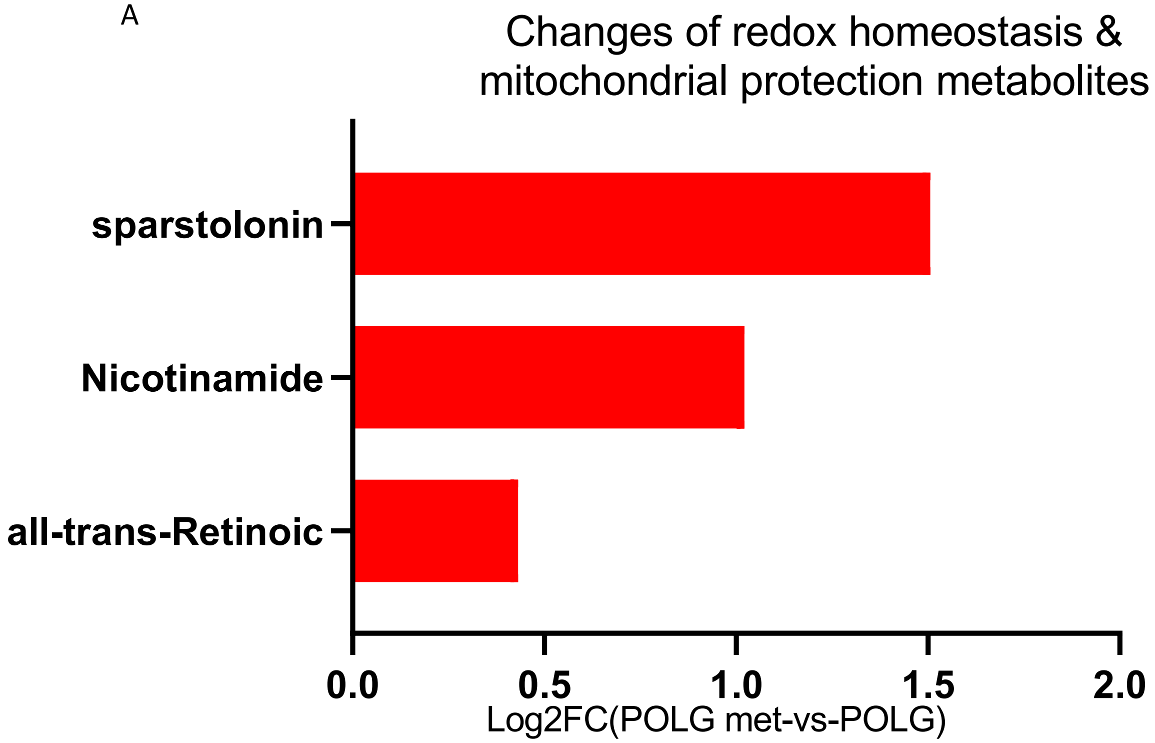

B

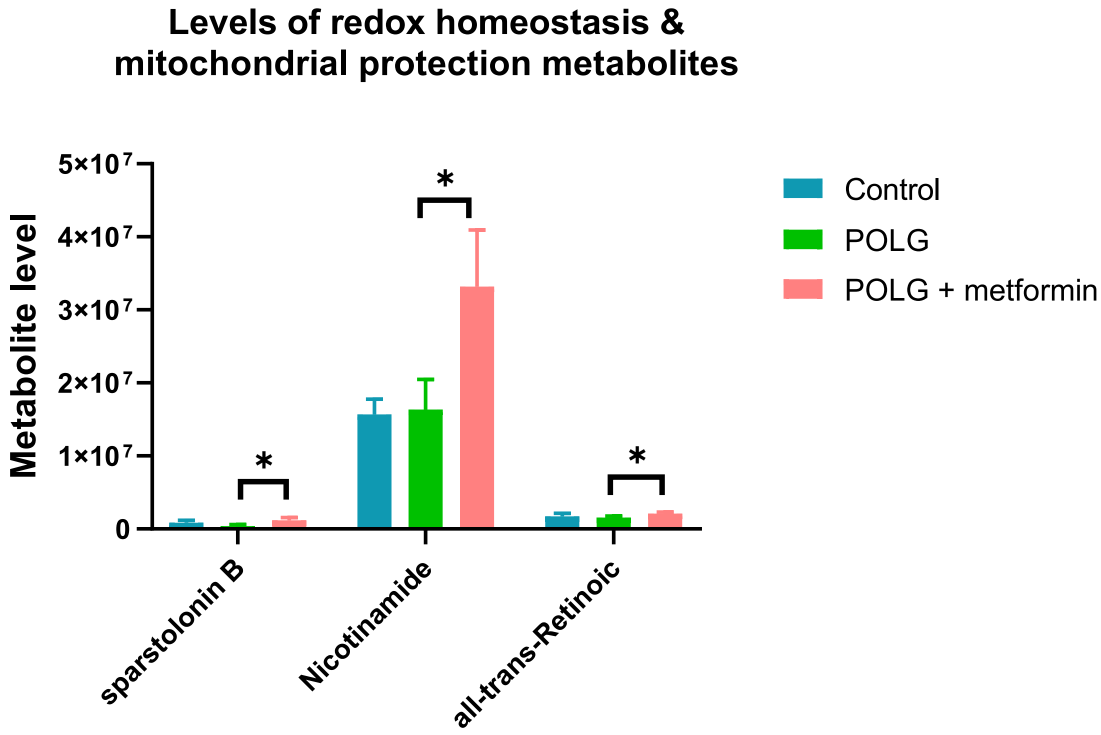

Figure S17. Metformin treatment enhances redox homeostasis & mitochondrial protection metabolites in POLG cortical organoids. (A) Pathway enrichment analysis revealed significant activation of redox homeostasis & mitochondrial protection metabolites following metformin treatment. (B) Quantification of selected upregulated redox homeostasis & mitochondrial protection metabolites in metformin-treated POLG organoids compared to untreated POLG samples. Data are expressed as mean ± SD from three independent replicates. Statistical significance was determined by using Mann–Whitney U test; $*p < 0.05$.

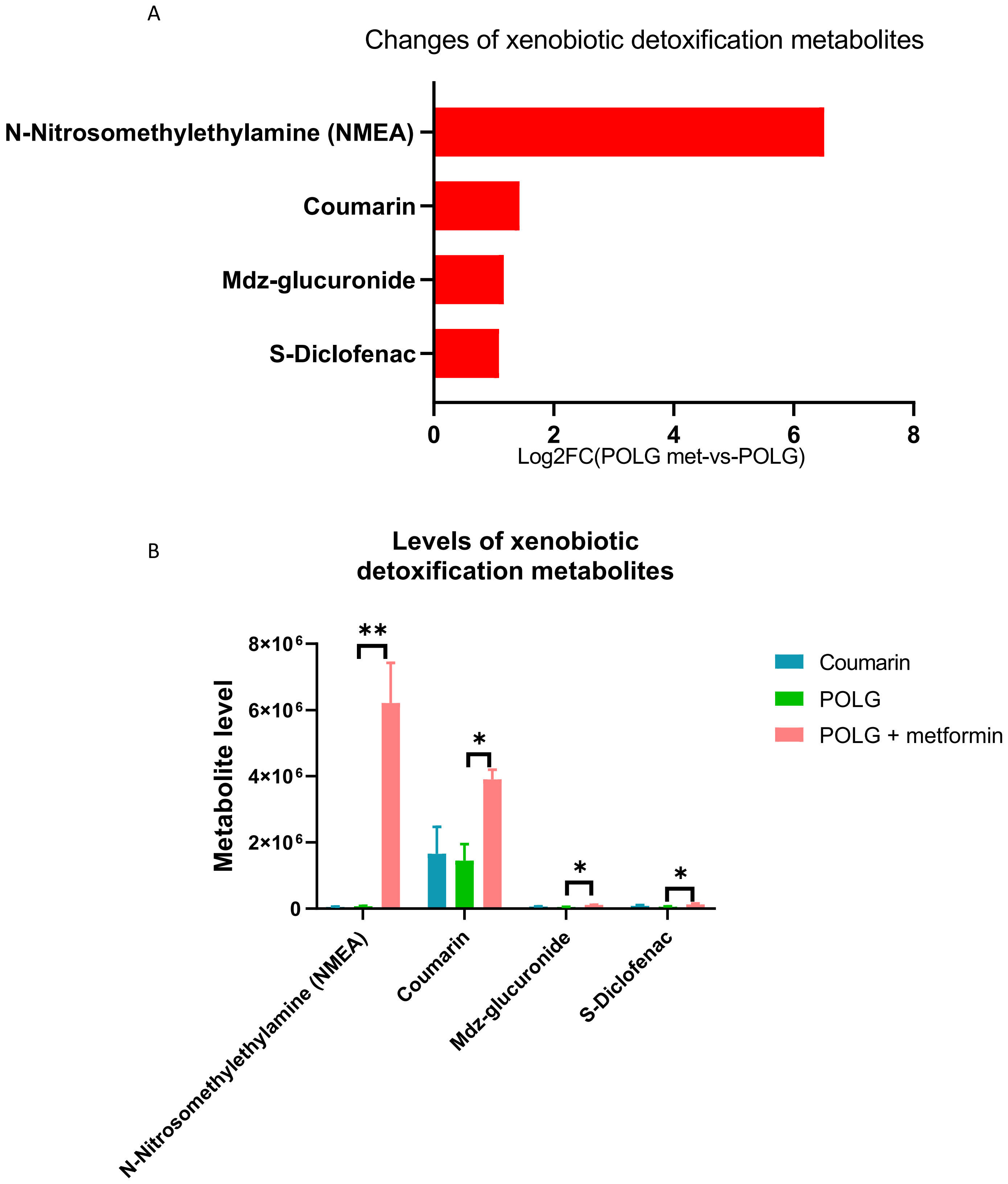

Figure S18. Metformin enhances xenobiotic detoxification pathways in POLG cortical organoids. (A) Pathway enrichment analysis showing significant upregulation of xenobiotic metabolism and detoxification-related pathways in metformin-treated POLG organoids. (B) Quantification of selected metabolites involved in xenobiotic clearance, demonstrating increased abundance in metformin-treated POLG samples compared to untreated controls. Data are presented as mean ± SD from three independent replicates. Statistical significance was assessed using Mann–Whitney U test; *$p < 0.05$.

Supplemental tables

Table S1. The list of cell types and the corresponding number of cells from each dataset, including La Manno et al., Birtele et al., and the present study.

Final_timepoint2	DA	Gabaergic	Glutamatergic	hDA0	hDA1	hDA2	hGaba	hHGgaba
6_7wks_LaManno	0	0	0	7	5	0	0	0
6wks_Britele	716	933	123	0	0	0	0	0
8wks_Britele	1018	347	2185	0	0	0	0	0
8wks_LaManno	0	0	0	4	2	0	0	0
9wks_LaManno	0	0	0	15	13	12	46	16
10wks_LaManno	0	0	0	16	18	21	10	3
11wks_Britele	1283	628	1756	0	0	0	0	0
11wks_LaManno	0	0	0	5	0	0	4	3
Cluster_a	2534	0	0	0	0	0	0	0
Cluster_b	1686	0	0	0	0	0	0	0
Cluster_c	0	0	439	0	0	0	0	0
Cluster_d	491	0	0	0	0	0	0	0
Cluster_e	0	0	3342	0	0	0	0	0
Cluster_h	1963	0	0	0	0	0	0	0
Cluster_i	0	1616	0	0	0	0	0	0

Table S2. Cell proportion of cells in different cell clusters from scRNA seq. related to Figure S6A.

Sample Name	*Dopaminergic a neurons*	*Dopaminergic b neurons*	*Glutaminergic c neurons*	*Dopaminergic d neurons*	*Glutaminergic e neurons*	*Neural progenitor f cells*	*Neural progenitor g cells*	*Dopaminergic h neurons*	*GABAergic i neurons*
Control	41.24294	31.12257	9.211496	0.36846	1.351019	2.407271	0.417588	12.13461	1.744043
CP2A	15.0289	3.757225	1.926782	1.1079	38.48748	1.782274	8.959538	18.25626	10.69364

Table S3. Number of cells in different cell clusters from scRNA seq. related to Figure S6B**.**

Sample Name	*Dopaminergic a neurons*	*Dopaminergic b neurons*	*Glutamatergic c neurons*	*Dopaminergic d neurons*	*Glutaminergic e neurons*	*Neural progenitor f cells*	*Neural progenitor g cells*	*Dopaminergic h neurons*	*GABAergic i neurons*
Control	1679	1267	375	15	55	98	17	494	71
CP2A	312	78	40	23	799	37	186	379	222

Table S4. Number of cells in different cell clusters from scRNA seq.

Sample Name	*Dopaminergic a neurons*	*Dopaminergic b neurons*	*Glutaminergic c neurons*	*Dopaminergic d neurons*	*Glutaminergic e neurons*	*Neural progenitor f cells*	*Neural progenitor g cells*	*Dopaminergic h neurons*	*GABAergic i neurons*
POLG	312	78	40	23	799	37	186	379	222
POLG metfomrin	543	341	24	453	2488	235	171	1090	1323

Table S5. Neuronal related GO enriched pathways downregulated in dopaminergic a neuron cluster. related to Figure 5E.

Gene Set	Term	P-value	Combined Score	Genes	Fold changes
GO_Biological_Process_2021	neuron differentiation (GO:0030182)	1.76E-09	214.9745	EPHA5;MEF2C;TENM3;SOX11;RORB;BHLHE22;PBX1;NR4A2;PTPRD;NEUROD2;LHX2;NEUROD6;ID2	8.755505
GO_Biological_Process_2021	generation of neurons (GO:0048699)	6.04E-06	80.90162	NR4A2;PTPRD;NEUROD2;MEF2C;LHX2;NEUROD6;MAP1B;SEMA3A;SOX11;BHLHE22	5.2193
GO_Biological_Process_2021	synaptic vesicle cycle (GO:0099504)	9.64E-05	178.3763	SNAP25;PCLO;SLC17A7;SNCA	4.015721
GO_Biological_Process_2021	negative regulation of neuron projection development (GO:0010977)	0.000106	108.7831	EFNB2;BCL11A;SEMA3A;PTPRO;RTN4	3.973884
GO_Biological_Process_2021	negative regulation of axon extension (GO:0030517)	0.00011	169.3119	MAP2;SEMA3A;SEMA3E;RTN4	3.958461
GO_Biological_Process_2021	neuron projection morphogenesis (GO:0048812)	0.000146	59.07298	SHTN1;MAP2;MAP1B;EPB41L3;SRGAP2;GAS7;MAP4K4	3.834263
GO_Biological_Process_2021	axonogenesis (GO:0007409)	0.000153	43.86821	EPHA5;EFNB2;SHTN1;MAP1B;SEMA3A;PTPRO;SEMA3E;PIK3R1;SPTBN1	3.815786
GO_Biological_Process_2021	central nervous system development (GO:0007417)	0.000346	35.43523	NR4A2;VCAN;PTPRZ1;NFIB;FOXG1;TBR1;SOX11;MEIS2;PBX1	3.46081
GO_Biological_Process_2021	regulation of neuron projection development (GO:0010975)	0.0004	44.00833	EFNB2;TENM3;ABI2;LRRC7;SEMA3A;PTPRO;TBR1	3.397699
GO_Biological_Process_2021	neurotransmitter secretion (GO:0007269)	0.000437	96.91676	SNAP25;PCLO;LIN7A;SNCA	3.35907
GO_Biological_Process_2021	neuron migration (GO:0001764)	0.000715	78.90276	NR4A2;MEF2C;MAP1B;SEMA3A	3.145877
GO_Biological_Process_2021	positive regulation of neuron differentiation (GO:0045666)	0.0004	100.551	NEUROD2;MEF2C;SOX11;TCF4	3.397733
GO_Biological_Process_2021	cell morphogenesis involved in neuron differentiation (GO:0048667)	0.003384	39.53672	SHTN1;MEF2C;MAP2;MAP1B	2.470562
GO_Biological_Process_2021	regulation of neuron differentiation (GO:0045664)	0.000456	65.44071	NEUROD2;MEF2C;ID2;SOX11;TCF4	3.341326
GO_Biological_Process_2021	positive regulation of neurogenesis (GO:0050769)	0.020702	20.96105	TIAM2;PTPRD;SOX11	1.683982
GO_Biological_Process_2021	chemical synaptic transmission (GO:0007268)	0.003551	19.21355	SDCBP;SNAP25;DLG2;EXOC4;LIN7A;SLC17A7;PRKACB;SNCA	2.449709
GO_Biological_Process_2021	regulation of axon extension (GO:0030516)	0.000247	122.4822	SHTN1;MAP2;SEMA3A;RTN4	3.607285
GO_Biological_Process_2021	neurotransmitter transport (GO:0006836)	0.021464	20.46799	SNAP25;LIN7A;SLC17A7	1.668287
GO_Biological_Process_2021	positive regulation of axonogenesis (GO:0050772)	0.047423	18.40066	TIAM2;SHTN1	1.324013
GO_Biological_Process_2021	positive regulation of neuron migration (GO:2001224)	0.004796	120.311	SHTN1;SEMA3A	2.319077
GO_Biological_Process_2021	neuron apoptotic process (GO:0051402)	0.012378	57.26462	FAM162A;BNIP3	1.907359
GO_Biological_Process_2021	axon development (GO:0061564)	0.026355	17.8393	SHTN1;MAP1B;RTN4	1.579141
GO_Biological_Process_2021	central nervous system neuron differentiation (GO:0021953)	0.03088	26.90557	NR4A2;MAP2	1.510322
GO_Biological_Process_2021	negative regulation of axon extension involved in axon guidance (GO:0048843)	0.011258	61.75382	SEMA3A;SEMA3E	1.948529
GO_Biological_Process_2021	regulation of neuron death (GO:1901214)	0.03275	15.35333	EFNB2;MEF2C;SNCA	1.484786
GO_Biological_Process_2021	negative regulation of neuron migration (GO:2001223)	0.047632	75.002	SRGAP2	1.322101
GO_Biological_Process_2021	regulation of neuron projection development (GO:0050773)	0.039735	21.57718	RAP2A;BCL11A	1.400824
GO_Biological_Process_2021	positive regulation of neuron differentiation (GO:0045666)	0.0004	100.551	NEUROD2;MEF2C;SOX11;TCF4	3.397733
GO_Biological_Process_2021	axon extension (GO:0048675)	0.016012			1.795548

Table S6. Neural related genes downregulated in dopaminergic a neuron cluster. related to Figure 5F.

Gene Name	Scores	p-values	Fold changes
FOSL2	3.203803	0.00135625	4.529042721
ACLY	3.209701	0.00132873	1.049625039
FAT3	3.216510	0.00129760	1.392369747
GNG5	3.219459	0.00128433	1.743123531
DYNC1I1	3.227770	0.00124759	2.4390347
PIK3R1	3.229754	0.00123897	1.207701325
KIF3B	3.241443	0.00118926	0.931640685
SLC24A2	3.246269	0.00116929	3.967341185
LPL	3.247126	0.00116577	5.890600681
FEZF2	3.262139	0.00110575	27.78674126
THRA	3.299672	0.00096798	0.959276199
RPL39	3.303908	0.00095347	0.18341884
RAI14	3.319189	0.00090279	2.818704128
SRGAP2	3.349269	0.00081025	1.362446308
SDCBP	3.354309	0.00079563	1.096875191
SEMA3E	3.366481	0.00076134	3.882170677
ADD2	3.379456	0.00072629	0.93006742
OPCML	3.380368	0.00072389	1.661402702
HDAC9	3.386588	0.00070768	1.864457488
AKT3	3.395220	0.00068573	1.122859001
MAP2	3.413182	0.00064209	0.149326816

Table S7. Mitochondrial related GO enriched pathways downregulated in dopaminergic a neuron cluster. related to Figure 5G.

Gene Set	Term	P-value	Combined Score	Genes	Fold changes
GO_Biological_Process_2021	mitochondrial protein catabolic process (GO:0035694)	0.047632	75.002	BNIP3	1.322101
GO_Biological_Process_2021	regulation of release of cytochrome c from mitochondria (GO:0090199)	0.043515	19.89196	FAM162A;BNIP3	1.361361
GO_Biological_Process_2021	regulation of release of cytochrome c from mitochondria (GO:0090200)	0.017313	43.68229	FAM162A;BNIP3	1.761625
GO_Biological_Process_2021	mitochondrial organization (GO:0007005)	0.045414	12.12399	MEF2C;PTPRZ1;SNCA	1.342815

Table S8. Mitochondrial related genes downregulated in dopaminergic a neuron cluster. related to Figure 5H.

Gene Name	Scores	p-values	Fold changes
MTCO1P12	-3.285410	0.00101834	0.769165695
MT-TL1	-3.414952	0.00063793	1.381238699
MT-ATP8	-4.752836	0.00000201	0.44138357
MT-ND4L	-4.908705	0.00000092	0.330361009
MT-ND3	-5.341834	0.00000009	0.25792563
MT-ATP6	-6.124554	0.00000091	0.317350477
MT-RNR2	-6.347499	0.00000022	0.355067611
MT-ND5	-9.707332	0.00000000	0.667137861
MT-CO3	-9.715427	0.00000000	0.583816469
MT-CO2	-17.564562	0.00000000	1.151174307

Table S9. Neuronal related GO enriched pathways downregulated in dopaminergic e neuron cluster. related to Figure 6E.

Gene_set	Term	Overlap	P-value
GO_Biological_Process _2021	neuroepithelial cell differentiation (GO:0060563)	2/6	0.000127354
GO_Biological_Process _2021	noradrenergic neuron differentiation (GO:0003357)	2/7	0.000177958
GO_Biological_Process _2021	glial cell development (GO:0021782)	3/26	6.04095E-05
GO_Biological_Process _2021	neural tube development (GO:0021915)	2/16	0.000999686
GO_Biological_Process _2021	autonomic nervous system development (GO:0048483)	2/21	0.001732951
GO_Biological_Process _2021	neuron differentiation (GO:0030182)	4/174	0.001735213
GO_Biological_Process _2021	substantia nigra development (GO:0021762)	3/43	0.000276703
GO_Biological_Process _2021	sympathetic nervous system development (GO:0048485)	2/16	0.000999686
GO_Biological_Process _2021	glial cell differentiation (GO:0010001)	2/29	0.003300019
GO_Biological_Process _2021	generation of neurons (GO:0048699)	4/202	0.002979561
GO_Biological_Process _2021	spinal cord development (GO:0021510)	2/13	0.000653503
GO_Biological_Process _2021	central nervous system neuron differentiation (GO:0021953)	2/34	0.004516979
GO_Biological_Process _2021	maintenance of blood-brain barrier (GO:0035633)	2/30	0.003529054
GO_Biological_Process _2021	central nervous system neuron development (GO:0021954)	1/15	0.043362459
GO_Biological_Process _2021	positive regulation of neuron migration (GO:2001224)	1/13	0.037689393
GO_Biological_Process _2021	spinal cord motor neuron differentiation (GO:0021522)	1/6	0.017572014
GO_Biological_Process _2021	positive regulation of oligodendrocyte differentiation (GO:0048714)	1/8	0.023361644

Table S10. Neural related genes downregulated in dopaminergic e neuron cluster. related to Figure 6F.

names	scores	pvals	Fold changes
SATB2	4.28966	1.79E-05	30.03789
DOK5	4.56997	4.88E-06	3.625031
NEFM	5.89069	3.85E-09	1.779695
BHLHE22	6.00994	1.86E-09	7.184215
GPR22	6.18456	6.23E-10	5.010109
TCF4	6.20717	5.39E-10	4.305514
ARPP21	6.31709	2.67E-10	3.220764
FABP7	6.84295	7.76E-12	1.661374
NELL2	6.98706	2.81E-12	2.549326
NFIB	7.02266	2.18E-12	4.973473
MEF2C	8.61323	7.1E-18	4.086001
NEUROD6	11.7136	1.09E-31	10.08239

Table S11. Neuronal related GO enriched pathways downregulated in dopaminergic e neuron cluster. related to Figure 6G.

Term	P-value	Combined Score	Genes	Fold changes
mitochondrial electron transport. NADH to ubiquinone (GO:0006120)	0.001557	248.776	NDUFS5;NDUFC2	2.807694
mitochondrial respiratory chain complex I assembly (GO:0032981)	0.003413	144.2821	NDUFS5;NDUFC2	2.466915
NADH dehydrogenase complex assembly (GO:0010257)	0.003413	144.2821	NDUFS5;NDUFC2	2.466915
aerobic electron transport chain (GO:0019646)	0.00493	111.0566	NDUFS5;NDUFC2	2.307113
mitochondrial ATP synthesis coupled electron transport (GO:0042775)	0.005069	108.872	NDUFS5;NDUFC2	2.295107
mitochondrial respiratory chain complex assembly (GO:0033108)	0.008026	77.86639	NDUFS5;NDUFC2	2.095487
NADH dehydrogenase (quinone) activity (GO:0050136)	0.001255	288.2955	NDUFS5;NDUFC2	2.901378
NADH dehydrogenase (ubiquinone) activity (GO:0008137)	0.001255	288.2955	NDUFS5;NDUFC2	2.901378
oxidoreduction-driven active transmembrane transporter activity (GO:0015453)	0.003413	144.2821	NDUFS5;NDUFC2	2.466915
mitochondrial respiratory chain complex I (GO:0005747)	0.001804	224.8408	NDUFS5;NDUFC2	2.743722
respiratory chain complex I (GO:0045271)	0.001804	224.8408	NDUFS5;NDUFC2	2.743722

Table S12. Neural related genes downregulated in dopaminergic e neuron cluster. related to Figure 6H.

names	scores	Fold changes	pvals	pvals_adj	Fold changes
NDUFC2	3.84773	0.80779	0.000119	0.031655	0.807786
MT-ATP6	4.10571	0.54972	4.03E-05	0.012932	0.549723
MT-RNR1	4.13256	0.77898	3.59E-05	0.011631	0.778979
NDUFS5	4.5663	0.80232	4.96E-06	0.002184	0.802316
MT-CYB	5.19021	0.79468	2.1E-07	0.000132	0.794679
MT-CO2	5.83079	0.91899	5.52E-09	4.47E-06	0.918993

Table S13. Part 1 of the Neuronal related GO enriched pathways downregulated in GABAergic I neuron cluster. related to Figure 7E.

Gene_set	Term	P-value	Combined Score	Genes	Fold changes
GO_Biological_Process_2021	regulation of neuron differentiation (GO:0045664)	6.54E-06	469.1031335	NEUROD2;ID2;SOX11;TCF4	5.184422252
GO_Biological_Process_2021	neuron differentiation (GO:0030182)	6.66E-06	268.5679519	NEUROD2;NEUROD6;ID2;SOX11;BHLHE22	5.176525771
GO_Biological_Process_2021	positive regulation of neuron differentiation (GO:0045666)	3.99E-05	540.7095225	NEUROD2;SOX11;TCF4	4.399027104
GO_Biological_Process_2021	negative regulation of developmental process (GO:0051093)	0.000228205	241.5417588	NEUROD2;BCL11A;NFIB	3.641674844
GO_Biological_Process_2021	generation of neurons (GO:0048699)	0.000256659	122.3059723	NEUROD2;NEUROD6;SOX11;BHLHE22	3.590643502
GO_Biological_Process_2021	positive regulation of cell differentiation (GO:0045597)	0.000647154	84.4361552	NEUROD2;ID2;SOX11;TCF4	3.18899236
GO_Biological_Process_2021	glial cell differentiation (GO:0010001)	0.000919668	356.1288723	NFIB;SOX11	3.036368925
GO_Biological_Process_2021	central nervous system development (GO:0007417)	0.008106288	38.35998548	NFIB;FOXG1;SOX11	2.091177971
GO_Biological_Process_2021	regulation of neural precursor cell proliferation (GO:2000177)	0.035067473	101.2606815	ID2	1.455095529
GO_Biological_Process_2021	neuron fate commitment (GO:0048663)	0.021489479	196.4996601	ID2	1.667774114
GO_Biological_Process_2021	regulation of synapse maturation (GO:0090128)	0.016922535	271.3831304	NEUROD2	1.771534579
GO_Biological_Process_2021	regulation of synapse maturation (GO:0090129)	0.013866454	355.8283387	NEUROD2	1.858034585
GO_Biological_Process_2021	neuroepithelial cell differentiation (GO:0060563)	0.009265092	623.0766346	SOX11	2.033150264
GO_Biological_Process_2021	autonomic nervous system development (GO:0048483)	0.032065989	114.3729154	SOX11	1.493955361
GO_Biological_Process_2021	sympathetic nervous system development (GO:0048485)	0.024522697	164.4278859	SOX11	1.610431768
GO_Biological_Process_2021	gliogenesis (GO:0042063)	0.026035892	151.6544079	NFIB	1.584427539
GO_Biological_Process_2021	glial cell development (GO:0021782)	0.03955282	85.89530575	SOX11	1.402822547
GO_Biological_Process_2021	regulation of astrocyte differentiation (GO:0048710)	0.018447136	241.4824938	ID2	1.73407105
GO_Biological_Process_2021	regulation of B cell differentiation (GO:0045577)	0.027546815	140.5195246	ID2	1.559928608
GO_Biological_Process_2021	positive regulation of glial cell differentiation (GO:0045687)	0.027546815	140.5195246	ID2	1.559928608

Table S14. Part 2 of the Neuronal related GO enriched pathways downregulated in GABAergic I neuron cluster. related to Figure 7E.

Gene_set	Term	P-value	Combined Score	Genes	Fold changes
GO_Biological_Process_2021	regulation of glial cell proliferation (GO:0060251)	0.013866454	355.8283387	SOX11	1.858034585
GO_Biological_Process_2021	regulation of nervous system development (GO:0007399)	0.02905547	130.736262	SOX11	1.536772095
GO_Biological_Process_2021	positive regulation of synaptic plasticity (GO:0031915)	0.010801183	502.1914056	NEUROD2	1.966528676
GO_Biological_Process_2021	regulation of neuron projection development (GO:0010975)	0.03056186	122.0800857	SOX11	1.514820218
GO_Biological_Process_2021	regulation of synapse organization (GO:0050807)	0.016922535	271.3831304	NEUROD2	1.771534579
GO_Biological_Process_2021	central nervous system projection neuron axonogenesis (GO:0021952)	0.01996945	216.9520847	NFIB	1.699633896
GO_Biological_Process_2021	spinal cord development (GO:0021510)	0.01996945	216.9520847	SOX11	1.699633896
GO_Biological_Process_2021	brain development (GO:0007420)	0.022537739	35.02908343	NFIB;FOXG1	1.647089655
GO_Biological_Process_2021	mammary gland epithelium development (GO:0061180)	0.023007227	179.2123814	ID2	1.638135723
GO_Biological_Process_2021	neural tube development (GO:0021915)	0.024522697	164.4278859	SOX11	1.610431768
GO_Biological_Process_2021	neuron projection guidance (GO:1902285)	0.016922535	271.3831304	NEUROD2	1.771534579
GO_Molecular_Function_2021	sequence-specific double-stranded DNA binding (GO:1990837)	0.000685154	47.71024896	NEUROD2;NFIB;FOXG1;SOX11;TCF4;BHLHE22	3.164211802
GO_Molecular_Function_2021	RNA polymerase II cis-regulatory region sequence-specific DNA binding (GO:0000978)	0.001578895	31.01922145	NEUROD2;BCL11A;NFIB;NEUROD6;SOX11;TCF4;BHLHE22	2.801646751
GO_Molecular_Function_2021	cis-regulatory region sequence-specific DNA binding (GO:0000987)	0.001578895	31.01922145	NEUROD2;BCL11A;NFIB;NEUROD6;SOX11;TCF4;BHLHE22	2.801646751
GO_Molecular_Function_2021	RNA polymerase II transcription regulatory region sequence-specific DNA binding (GO:0000977)	0.004083433	22.09260938	NEUROD2;BCL11A;NFIB;NEUROD6;SOX11;TCF4;BHLHE22	2.388974566
GO_Molecular_Function_2021	DNA-binding transcription activator activity. RNA polymerase II-specific (GO:0001228)	0.014566216	26.96568873	NFIB;SOX11;TCF4	1.836653254
GO_Molecular_Function_2021	protein heterodimerization activity (GO:0046982)	0.034190207	24.76224266	NEUROD2;TCF4	1.46609827
Reactome_2016	Cholesterol biosynthesis Homo sapiens R-HSA-191273	5.85E-06	1287.737575	SQLE;MSMO1;FDFT1	5.233079533

Table S15. Neuronal related DEGs upregulated in GABAergic I neuron cluster in patient organoid with treatment compared to untreated samples. related to Figure 7F.

names	scores	p valus	Fold changes
SOX11	3.867723465	0.000109856	1.109950542
ID2	3.874964952	0.00010664	2.300649643
STMN4	3.955425262	7.64E-05	1.112474203
NEUROD2	4.086575508	4.38E-05	3.698160172
TCF4	4.093012333	4.26E-05	1.693955898
BCL11A	4.125196457	3.70E-05	2.220225096
FOXG1	4.46554327	7.99E-06	30.0363121
NELL2	4.513014793	6.39E-06	2.407956362
BHLHE22	7.561653137	3.98E-14	4.091328144
NEUROD6	10.81466007	2.93E-27	6.7858181

Table S16. Mitochondrial related GO enriched pathways downregulated in GABAergic I neuron cluster. related to Figure 7G.

**Term**	**P-value**	**Combined Score**	**Genes**	**Fold changes**
regulation of ATP biosynthetic process (GO:2001169)	0.024522697	164.4278859	TMSB4X	1.610431768
positive regulation of ATP biosynthetic process (GO:2001171)	0.013866454	355.8283387	TMSB4X	1.858034585
positive regulation of ATP metabolic process (GO:1903580)	0.049940305	62.23930367	TMSB4X	1.30154881
mitochondrial organization (GO:0007005)	0.03056186	122.0800857	TMSB4X	1.514820218

Table S17. Neuronal related DEGs upregulated in GABAergic I neuron cluster in patient organoid with treatment compared to untreated samples. related to Figure 7H.

names	scores	p valus	Fold changes
MT-ND3	3.86933279	0.000109134	0.796117604
MT-RNR1	4.350484848	1.36E-05	0.842556238
MT-ND5	4.397151947	1.10E-05	0.875798345
MT-ND4	4.992557526	5.96E-07	0.700720549
MT-ATP6	5.427042961	5.73E-08	0.879056573
MT-CO1	6.130265236	8.77E-10	1.14920187
MT-CO2	6.133483887	8.60E-10	1.235617995
MT-CO3	6.969465733	3.18E-12	1.244438529
MT-CYB	-7.760389805	8.47E-15	1.271402478

Table S18. Neuronal related GO enriched pathways upregulated in GABAergic i neuronsin patient organoid with treatment compared to untreated samples. related to Figure 8D.

Gene_set	Term	P-value	Combined Score	Genes	Fold changes
GO_Biological_Process_2021	cerebellar Purkinje cell differentiation (GO:0021702)	0.004741	1484.797	LHX1	2.324094
GO_Biological_Process_2021	anterograde dendritic transport (GO:0098937)	0.004741	1484.797	KIF5C	2.324094
GO_Biological_Process_2021	anterograde dendritic transport of neurotransmitter receptor complex (GO:0098971)	0.004741	1484.797	KIF5C	2.324094
GO_Biological_Process_2021	forebrain regionalization (GO:0021871)	0.004741	1484.797	LHX1	2.324094
GO_Biological_Process_2021	trigeminal nerve development (GO:0021559)	0.005687	1147.411	TFAP2A	2.245108
GO_Biological_Process_2021	synaptic vesicle transport (GO:0048489)	0.010403	506.5602	KIF5C	1.982842
GO_Biological_Process_2021	cell differentiation in spinal cord (GO:0021515)	0.010403	506.5602	LHX1	1.982842
GO_Biological_Process_2021	synaptic vesicle localization (GO:0097479)	0.012283	406.7303	KIF5C	1.910681
GO_Biological_Process_2021	cranial nerve development (GO:0021545)	0.012283	406.7303	TFAP2A	1.910681
GO_Biological_Process_2021	axon guidance (GO:0007411)	0.015649	48.1313	KIF5C;LHX1	1.805513
GO_Biological_Process_2021	positive regulation of neuron apoptotic process (GO:0043525)	0.022565	182.772	TFAP2A	1.646559
GO_Biological_Process_2021	regulation of spindle assembly (GO:0090169)	0.023495	173.2826	CHMP2B	1.629025
GO_Biological_Process_2021	regulation of transport (GO:0051049)	0.034586	103.5511	ACTB	1.4611
GO_Biological_Process_2021	positive regulation of neuron differentiation (GO:0045666)	0.040087	84.8387	PCP4	1.397001
GO_Biological_Process_2021	substantia nigra development (GO:0021762)	0.040087	84.8387	ACTB	1.397001
GO_Biological_Process_2021	positive regulation of reactive oxygen species metabolic process (GO:2000379)	0.045558	71.26001	ROMO1	1.34144

Table S19. Neuronal related DEGs upregulated in Glutaminergic e neuron cluster in patient organoid with treatment compared to untreated samples. related to Figure 8E.

Gene	Scores	P-value	Fold changes
ACTB	14.363254	8.80E-47	0.417558
TUBA1A	14.149615	1.88E-45	0.372790
RPS27	9.394007	5.78E-21	0.424174
PFN1	7.764236	8.21E-15	0.497129
TBCB	7.238196	4.55E-13	0.182682
RPL18A	5.891133	3.84E-09	0.195498
RPL15	5.645402	1.65E-08	0.105016
TUBB	5.614123	1.98E-08	0.169272
TUBB2B	4.648511	3.34E-06	0.121281
NEFL	3.760795	0.000169	0.237623

Table S20. Mitochondrial related GO enriched pathways upregulated in GABAergic i neurons in patient organoid with treatment compared to untreated samples. related to Figure 8F.

Gene_set	Term	P-value	Combined Score	Genes	Fold changes
GO_Biological_Process_2021	aerobic electron transport chain (GO:0019646)	0.001987	214.3295	NDUFB2;COX6C	2.701826
GO_Biological_Process_2021	mitochondrial ATP synthesis coupled electron transport (GO:0042775)	0.002043	210.2617	NDUFB2;COX6C	2.689662
GO_Biological_Process_2021	protein insertion into mitochondrial inner membrane (GO:0045039)	0.011344	451.7557	ROMO1	1.945248
GO_Biological_Process_2021	mitochondrial electron transport. cytochrome c to oxygen (GO:0006123)	0.016034	286.5143	COX6C	1.794956
GO_Biological_Process_2021	protein import into mitochondrial matrix (GO:0030150)	0.017904	247.8562	ROMO1	1.747042
GO_Biological_Process_2021	protein insertion into mitochondrial membrane (GO:0051204)	0.022565	182.772	ROMO1	1.646559
GO_Biological_Process_2021	intracellular protein transmembrane transport (GO:0065002)	0.041	82.28088	ROMO1	1.387211
GO_Biological_Process_2021	establishment of protein localization to mitochondrion (GO:0072655)	0.044648	73.25513	ROMO1	1.3502
GO_Biological_Process_2021	inner mitochondrial membrane organization (GO:0007007)	0.045558	71.26001	ROMO1	1.34144
GO_Molecular_Function_2021	NADH dehydrogenase (quinone) activity (GO:0050136)	0.032746	111.4353	NDUFB2	1.484845
GO_Molecular_Function_2021	NADH dehydrogenase (ubiquinone) activity (GO:0008137)	0.032746	111.4353	NDUFB2	1.484845

Table S21. Mitochondrial related DEGs upregulated in GABAergic i neurons in patient organoid with treatment compared to untreated samples. related to Figure 8G.

Gene	Scores	P-value	Fold changes
PSMA7	19.524607	6.78E-85	0.883459
HMGCS1	7.063742	1.62E-12	0.628155
ACAT2	5.677174	1.37E-08	0.626757
ATP5PF	5.035424	4.77E-07	0.191826
COX6C	4.183743	2.87E-05	0.142184
ATP5MF	3.449015	0.000563	0.080810
ATP5F1D	3.323000	0.000891	1.111357
NDUFA4	3.782026	0.000156	0.119858
ROMO1	3.003721	0.002667	0.148930
TMEM160	3.103342	0.001913	0.638078

Table S22. Neuronal related GO enriched pathways upregulated in Glutaminergic e neuron cluster in patient organoid with treatment compared to untreated samples. related to Figure 8H.

Gene_set	Term	P-value	Combined Score	Genes	Fold changes
GO_Biological_Process_2021	neuroepithelial cell differentiation (GO:0060563)	0.000127	1568.472	SOX11;SOX4	3.894987
GO_Biological_Process_2021	noradrenergic neuron differentiation (GO:0003357)	0.000178	1207.907	SOX11;SOX4	3.749683
GO_Biological_Process_2021	neural tube development (GO:0021915)	0.001	345.0053	SOX11;SOX4	3.000136
GO_Biological_Process_2021	sympathetic nervous system development (GO:0048485)	0.001	345.0053	SOX11;SOX4	3.000136
GO_Biological_Process_2021	neuron differentiation (GO:0030182)	0.001735	53.76549	SOX11;LHX9;SOX4;ITM2C	2.760647
GO_Biological_Process_2021	generation of neurons (GO:0048699)	0.00298	42.17627	TUBB2B;SOX11;LHX9;ITM2C	2.525848
GO_Biological_Process_2021	central nervous system neuron differentiation (GO:0021953)	0.004517	117.8803	NPY;SOX4	2.345152
GO_Biological_Process_2021	spinal cord motor neuron differentiation (GO:0021522)	0.017572	277.8286	SOX4	1.755178
GO_Biological_Process_2021	positive regulation of neuron migration (GO:2001224)	0.037689	93.87179	MDK	1.423781
GO_Biological_Process_2021	central nervous system neuron development (GO:0021954)	0.043362	77.01249	NPY	1.362886
GO_Biological_Process_2021	regulation of neuron projection development (GO:0010975)	0.085341	10.47801	MDK;ITM2C	1.068841
GO_Biological_Process_2021	negative regulation of neuron projection development (GO:0010977)	0.157682	11.10988	ITM2C	0.802218
GO_Biological_Process_2021	cell morphogenesis involved in neuron differentiation (GO:0048667)	0.201447	7.317222	ACTB	0.69584
GO_Biological_Process_2021	axon development (GO:0061564)	0.20852	6.883204	ACTB	0.680852
GO_Biological_Process_2021	regulation of neuron differentiation (GO:0045664)	0.20852	6.883204	SOX11	0.680852
GO_Biological_Process_2021	positive regulation of neuron projection development (GO:0010976)	0.229373	5.793327	MDK	0.639457
GO_Biological_Process_2021	negative regulation of neuron death (GO:1901215)	0.25191	4.862885	MDK	0.598754
GO_Biological_Process_2021	regulation of neuron apoptotic process (GO:0043523)	0.25191	4.862885	MDK	0.598754
GO_Biological_Process_2021	neuron projection development (GO:0031175)	0.397915	1.847799	NPY	0.400209
GO_Cellular_Component_2021	neuronal dense core vesicle (GO:0098992)	0.014665	362.8467	NPY	1.833731
GO_Cellular_Component_2021	neuron projection (GO:0043005)	0.225575	2.796828	NEFL;NEFM;ACTB	0.646709
Reactome_2016	Neuronal System Homo sapiens R-HSA-112316	0.591804	0.592142	NEFL	0.227822

Table S23. Neuronal related DEGs upregulated in Glutaminergic e neuron cluster in patient organoid with treatment compared to untreated samples. related to Figure 8I.

Name	Score	P-value	Fold changes
BLCAP	8.650375	5.13302E-18	1.128448
TFAP2A	4.556397	5.20385E-06	1.750401
CHMP2B	4.450404	8.57088E-06	1.625380
LHX1	4.170953	3.03328E-05	1.267747
KIF5C	4.117469	3.83056E-05	0.824310
PCP4	3.881342	0.000103882	1.886388

Table S24. Mitochondrial related GO enriched pathways upregulated in Glutaminergic e neuron cluster in patient organoid with treatment compared to untreated samples. related to Figure 8G.

Term	P-value	Combined Score	Genes	Fold changes
inner mitochondrial membrane organization (GO:0007007)	1.31E-05	361.5072	ATP5PF;ROMO1;ATP5F1D;ATP5MF	4.882595
mitochondrial ATP synthesis coupled proton transport (GO:0042776)	1.61E-05	841.5596	ATP5PF;ATP5F1D;ATP5MF	4.793162
mitochondrial electron transport. cytochrome c to oxygen (GO:0006123)	0.001131	316.2432	NDUFA4;COX6C	2.946602
positive regulation of mitochondrial depolarization (GO:0051901)	0.014665	362.8467	MLLT11	1.833731
mitochondrial proton-transporting ATP synthase complex assembly (GO:0033615)	0.017572	277.8286	ATP5F1D	1.755178
mitochondrial ATP synthesis coupled electron transport (GO:0042775)	0.018662	40.23163	NDUFA4;COX6C	1.729037
axonal transport of mitochondrion (GO:0019896)	0.031983	118.2988	NEFL	1.495077
regulation of mitochondrial depolarization (GO:0051900)	0.031983	118.2988	MLLT11	1.495077
protein insertion into mitochondrial inner membrane (GO:0045039)	0.03484	104.866	ROMO1	1.457916
mitochondrion transport along microtubule (GO:0047497)	0.037689	93.87179	NEFL	1.423781
positive regulation of mitochondrial translation (GO:0070131)	0.04053	84.72602	C1QBP	1.392223
protein import into mitochondrial matrix (GO:0030150)	0.05461	55.48547	ROMO1	1.262727
regulation of mitochondrial translation (GO:0070129)	0.060185	48.26257	C1QBP	1.220511
protein insertion into mitochondrial membrane (GO:0051204)	0.068487	40.03175	ROMO1	1.164391
positive regulation of release of cytochrome c from mitochondria (GO:0090200)	0.071238	37.79829	MLLT11	1.147286
mitochondrial electron transport. NADH to ubiquinone (GO:0006120)	0.108927	20.02106	NDUFA4	0.962863
regulation of release of cytochrome c from mitochondria (GO:0090199)	0.114187	18.61356	MLLT11	0.942382
establishment of protein localization to mitochondrion (GO:0072655)	0.132358	14.75809	ROMO1	0.87825
protein targeting to mitochondrion (GO:0006626)	0.150162	12.03913	ROMO1	0.82344
positive regulation of mitochondrion organization (GO:0010822)	0.157682	11.10988	MLLT11	0.802218
mitochondrion organization (GO:0007005)	0.40505	1.770148	ATP5F1D	0.392492
mitochondrial proton-transporting ATP synthase complex (GO:0005753)	1.93E-05	772.5907	ATP5PF;ATP5F1D;ATP5MF	4.714892
mitochondrial inner membrane (GO:0005743)	0.002792	33.07337	ATP5PF;NDUFA4;COX6C;ATP5F1D;ATP5MF	2.554079
mitochondrial membrane (GO:0031966)	0.012305	17.09255	ATP5PF;NDUFA4;COX6C;ATP5F1D;ATP5MF	1.909901
mitochondrial proton-transporting ATP synthase complex. catalytic sector F(1) (GO:0000275)	0.014665	362.8467	ATP5F1D	1.833731
mitochondrial respiratory chain complex IV (GO:0005751)	0.029118	135.0339	NDUFA4	1.535842
respiratory chain complex I (GO:0045271)	0.116806	17.96893	NDUFA4	0.932535
mitochondrial respiratory chain complex I (GO:0005747)	0.116806	17.96893	NDUFA4	0.932535
mitochondrial matrix (GO:0005759)	0.274107	2.571807	C1QBP;ATP5F1D	0.56208

Table S25. Mitochondrial related DEGs upregulated in Glutaminergic e neuron cluster in patient organoid with treatment compared to untreated samples. related to Figure 8K.

**Name**	**Score**	**P-value**	**Fold changes**
PSMA7	9.008671	2.08569E-19	0.665129
TUBA1A	7.942889	1.97526E-15	0.433231
COX6C	4.125516	3.69904E-05	0.344791
NDUFB2	3.816316	0.000135459	0.499353
ROMO1	3.682362	0.000231083	0.593688

Table S26. List of the metabolites related to energy metabolism (TCA cycle & electron transport chain) in patient organoid with treatment compared to untreated samples, related to Fig. S14.

Name	HMDB ID	Log2FC(G2-vs-G1)	Pvalue(G2-vs-G1)	Pathway
3-deoxy-D-arabino-heptulosonate-7-phosphate	HMDB0304124	4.828060975831878	1.3319137761549E-4	Shikimate pathway (ROS modulation)
FADH	HMDB0001197	0.9912032059912164	8.5093127945136E-4	ETC substrate (Complex II)
2-Oxo-4-methylthiobutanoic acid	HMDB0001553	0.732344081159056	0.0342819801644299	TCA cycle (methionine branch)
alpha-D-glucose 6-phosphate	HMDB0304534	0.6325249932162227	0.0265448413506918	Glycolysis → pyruvate for mitochondria
3-deoxy-D-arabino-heptulosonate-7-phosphate	HMDB0304124	4.828060975831878	1.3319137761549E-4	Shikimate pathway (ROS modulation)

Table S27. List of the metabolites related to amino acid/nucleotidemetabolism metabolites in patient organoid with treatment compared to untreated samples, related to Fig. S15.

Name	HMDB ID	Log2FC(G2-vs-G1)	Pvalue(G2-vs-G1)	Pathway
L-Methionine sulfoxide	HMDB0002005	4.291167243697032	0.018895577173434	1-carbon metabolism (folate cycle)
O-Acetylserine	HMDB0003011	2.7609127906943	0.0010886832668507	Cysteine synthesis precursor
L-Arginine	HMDB0000517	2.3371885365462206	0.0255867314767439	Urea cycle (ornithine transport)
L-Tyrosine	HMDB0000158	2.1944300679164632	0.0341115370029525	Aromatic amino acid metabolism
N-a-Acetyl-L-arginine	HMDB0004620	1.1285711772322298	0.0384520589285175	Urea cycle (N-acetylglutamate synthesis)
N5-Carboxyaminoimidazole ribonucleotide	HMDB0012268	0.7385052080367023	0.0204940917218998	Purine synthesis intermediate
beta-Alanine	HMDB0002005	0.5410542900559638	0.018895577173434	1-carbon metabolism (folate cycle)

Table S28. List of the metabolites related to neuroprotective metabolites in patient organoid with treatment compared to untreated samples, related to Fig. S16.

Name	HMDB ID	Log2FC(G2-vs-G1)	Pvalue(G2-vs-G1)	Pathway
Cholecalciferol	HMDB0000876	3.683496015958201	0.0214109764555771	Neurotrophic factor regulation
sparstolonin B	HMDB0258408	1.5062919391014788	0.031991797920373	Anti-inflammatory/Neuroprotection
Nicotinamide	HMDB0001406	1.0219800359538034	0.024622302120967	NAD+ precursor (antioxidant)
all-trans-Retinoic acid	HMDB0001852	0.4321649546011857	0.0412130749002353	Neuronal differentiation signaling

Table S29. List of the metabolites related to redox homeostasis & mitochondrial protection in patient organoid with treatment compared to untreated samples, related to Fig. S17.

Name	HMDB ID	Log2FC(G2-vs-G1)	Pvalue(G2-vs-G1)	Pathway
sparstolonin B	HMDB0258408	1.50629193910147<br>88	0.03199179792037<br>3	Inhibits mitochondrial ROS
Nicotinamide	HMDB0001406	1.02198003595380<br>34	0.02462230212096<br>7	NAD+ precursor (antioxidant defense)
all-trans-Retinoic acid	HMDB0001852	0.43216495460118<br>57	0.04121307490023<br>53	PPARγ-mediated biogenesis
all-trans-Retinoic acid	HMDB0258408	1.50629193910147<br>88	0.03199179792037<br>3	Inhibits mitochondrial ROS

Table S30. List of the metabolites related to xenobiotic detoxification in patient organoid with treatment compared to untreated samples, related to Fig. S18.

Name	HMDB ID	Log2FC(G2-vs-G1)	Pvalue(G2-vs-G1)	Pathway
N-Nitrosomethylethylamine (NMEA)		6.508106603129823	2.64513430474634E-5	Carcinogen detoxification target
Coumarin	HMDB0001218	1.430876681917851	0.0413494872324914	Hepatic enzyme CYP2A6 substrate
Mdz-glucuronide	HMDB0247486	1.165288454625675	0.0167820476745049	Cytochrome P450 metabolite
S-Diclofenac	HMDB0247974	1.0869474081172643	0.0150957547283399	Phase II drug metabolism (glucuronidation)

Table S31. Summary of the results in this study.

Cluster / Cell Type	POLG Organoid Alteration	Response to Metformin	Key GO Terms Affected
Dopaminergic a neurons	↓ cell number, ↓ mitochondrial genes (e.g., MT-CO2, MT-ND5), ↓ neuronal genes	↑ cell number, partial restoration observed	Oxidative phosphorylation, synaptic transmission, axonogenesis
Glutamatergic e neurons	↑ cell number, ↓ synaptic and mitochondrial pathways (e.g., MT-CO2, MT-ATP6)	↑ neuronal differentiation (e.g., TFAP2A, LHX1), ↑ mitochondrial function	Neuron projection, synaptic plasticity, mitochondrial organization
GABAergic i neurons	↑ cell number, ↓ cholesterol biosynthesis, ↓ synaptic signaling, ↓ mitochondrial function	↑ axonal structure genes (e.g., NEFL, TUBB3), ↑ mitochondrial efficiency genes	Cholesterol biosynthesis, neuron migration, ATP metabolic process

Table S32. Sample size and P value in individual figures.

Figure	Sample size	p value
2A, d	POLG:5, POLG+metformin:5	0.0317
2A, e	POLG:5, POLG+metformin:6	0.0022
2A, f	POLG:6, POLG+metformin:6	0.0043
2A, g	POLG:4, POLG+metformin:4	0.0286
2A, h	POLG:9, POLG+metformin:7	0.0002
2A, h	POLG:4, POLG+metformin:4	0.0286
2B	Control:4, Control+metformin:4<br>POLG:4, POLG+metformin:4	0.0286; 0.0286
2C	Control:4, Control+metformin:4<br>POLG:4, POLG+metformin:4	0.0286; 0.0286
2D	Control:4, Control+metformin:4<br>POLG:4, POLG+metformin:4	0.8857; 0.0286
2E	Control:4, Control+metformin:4<br>POLG:4, POLG+metformin:4	0.0286; 0.0286
2F	Control:4, Control+metformin:4<br>POLG:4, POLG+metformin:4	0.0286; 0.0286
